# Psychometric properties of measures of substance use: a systematic review and meta-analysis of reliability, validity and diagnostic test accuracy

**DOI:** 10.1186/s12874-020-00963-7

**Published:** 2020-05-07

**Authors:** Glenn-Milo Santos, Steffanie A. Strathdee, Nabila El-Bassel, Poonam Patel, Divya Subramanian, Danielle Horyniak, Ryan R. Cook, Charlotte McCullagh, Phillip Marotta, Foram Choksi, Brian Kang, Isabel Allen, Steven Shoptaw

**Affiliations:** 1 Department of Community Health Systems, University of California San Francisco, 25 Van Ness Avenue, Suite 500, San Francisco, CA 94102 USA; 2 Center for Public Health Research, San Francisco Department of Public Health, San Francisco, CA, USA; 3 Division of Global Public Health, University of California San Diego, San Diego, CA, USA; 4 School of Social Work, Columbia University, New York, NY, USA; 5 Burnet Institute, Melbourne, VIC Australia; 6 Monash University, School of Public Health and Preventive Medicine, Melbourne, VIC Australia; 7 Department of Family Medicine and Psychiatry and Biobehavioral Sciences, University of California Los Angeles, Los Angeles, CA, USA; 8 Department of Epidemiology and Biostatistics, University of California San Francisco, San Francisco, CA, USA

**Keywords:** Substance use, Alcohol, Drugs, Psychometric properties, Meta-analysis

## Abstract

**Background:**

Synthesis of psychometric properties of substance use measures to identify patterns of use and substance use disorders remains limited. To address this gap, we sought to systematically evaluate the psychometric properties of measures to detect substance use and misuse.

**Methods:**

We conducted a systematic review and meta-analysis of literature on measures of substance classes associated with HIV risk (heroin, methamphetamine, cocaine, ecstasy, alcohol) that were published in English before June 2016 that reported at least one of the following psychometric outcomes of interest: internal consistency (alpha), test-retest/inter-rater reliability (kappa), sensitivity, specificity, positive predictive value, and negative predictive value. We used meta-analytic techniques to generate pooled summary estimates for these outcomes using random effects and hierarchical logistic regression models.

**Results:**

Findings across 387 paper revealed that overall, 65% of pooled estimates for alpha were in the range of fair-to-excellent; 44% of estimates for kappa were in the range of fair-to-excellent. In addition, 69, 97, 37 and 96% of pooled estimates for sensitivity, specificity, positive predictive value, and negative predictive value, respectively, were in the range of moderate-to-excellent.

**Conclusion:**

We conclude that many substance use measures had pooled summary estimates that were at the fair/moderate-to-excellent range across different psychometric outcomes. Most scales were conducted in English, within the United States, highlighting the need to test and validate these measures in more diverse settings. Additionally, the majority of studies had high risk of bias, indicating a need for more studies with higher methodological quality.

## Background

Substance use, including illicit drug use and alcohol, is prevalent worldwide with about 5% of adults using illicit substances [1] and 40% of adults consuming alcohol, in the past year [2]. Moreover, the number of people with drug use disorders was estimated at 62 million, while the number of individuals with alcohol use disorders was estimated at 100.4 million in 2016 [3]. Substance use disorders are associated with substantial morbidity and mortality globally. Illicit drug use disorders were attributed to 20 million disability-adjusted life years (DALYs) lost [4] while alcohol use disorders were attributed to 85 million DALYs lost in 2012 [5]. Specific classes of substances also play an important role in HIV risk, including needle sharing, and sexual risk behaviors, and have been linked to HIV incidence [6–8] [6, 9–11] [12–15]. Among people living with HIV (PLWH), substance use disorders may lead to less optimal HIV care outcomes because of their associations with lower likelihood of being linked to HIV care, retained in care, receiving antiretroviral therapy (ART), having high ART adherence and lower likelihood of having an undetectable HIV viral load [9, 10, 16–18].

Given the role of substance use in the global burden of disease and the overlap between use of specific substances and HIV, it is important for clinicians and researchers to have tools with high reliability, validity, and diagnostic accuracy [19]. Yet too few use measures with known psychometric properties when assessing substance use. Currently, there are a myriad of standardized questionnaires used to screen substance use and misuse that require patients to self-report patterns of use and substance-related problems. Examples such as the Alcohol Use Disorders Identification Test and the Drug Use Disorders Identification test [20, 21] provide scores that correspond with severity of substance use and related problems. It remains that there are no biological measures that define a substance use disorder; existing biological measures are considered to be indirect correlates of use disorders [22]. Examples include alcohol biomarkers like Carbohydrate-Deficient Transferrin (CDT), and Gamma Glutamyl Transferase (GGT), which are used to screen for alcohol dependence and heavy drinking, respectively [22]. There is a great need to evaluate the psychometric performance of these measures and markers across studies in settings of HIV to elucidate the overall validity, reliability, and diagnostic accuracy.

One approach to informing the use of psychometric measures in research and clinical care is pooling the psychometric characteristics of measures across studies involves the use of meta-analytic techniques, which generates summary estimates of the validity, reliability, and diagnostic accuracy of different questionnaires [23–27]. However, synthesis of psychometric properties of substance use measures to identify patterns of use and substance use disorders remains limited, with few exceptions [21, 28, 29]. One meta-analysis focused on the accuracy of self-reported assessments to diagnose alcohol and cannabis use disorders found that instruments had a pooled sensitivity of 0.88 and a pooled specificity of 0.90 among emergency room department pediatric patients [28]. Another meta-analysis observed that studies with single questions to identify alcohol use disorders in primary care had pooled sensitivity of 0.54 and pooled specificity of 0.87 while two-question measures had a pooled sensitivity of 0.87 and a pooled specificity of 0.80 [29]. More commonly, however, reviews on substance use measures present psychometric data in a descriptive fashion [19, 30, 31]. Therefore, more rigorous efforts to systematically pool the psychometric properties of substance use measures are needed to establish the overall performance and accuracy of these tools and point toward their utility in future research.

To address these gaps, we conducted a systematic review and meta-analysis of literature to identify studies that have reported validity and reliability of substance use measures and pooled these measure using meta-analytic techniques. For the purposes of this review, we targeted our search for measures of substance classes previously associated with HIV risk. Specifically, we focused our review on measures for the following: alcohol, methamphetamine and amphetamine, cocaine, heroin, and ecstasy, regardless of whether the study was conducted among a population at high risk for HIV. Additionally, we included measures that evaluated substance use in general (i.e., measures that did not differentiate between classes of substances) as long as those measures were inclusive of our targeted substance classes. This study’s review questions are: What are the summary reliability, validity--as measured by alpha and kappa coefficients—and diagnostic accuracy—as measured by sensitivity, specificity, positive predictive value, and negative predictive value—of various substance and alcohol measures to screen for use and use disorders?

## Methods

### Search strategy

We conducted a systematic review of studies published prior to June 2016 on substance use measures indexed in electronic databases including PubMed, PsycINFO, and EMBASE. We developed Boolean search terms to capture substance use measures that have been previously associated with HIV risk, in consultation with the reference librarian from the University of California San Francisco with a master’s degree in library and information science (MLIS). The following substance classes were included: alcohol, methamphetamine and amphetamine, cocaine, heroin, and 3,4-methylenedioxy-methamphetamine (MDMA; “ecstasy”). Because the focus of this study was to pool psychometric properties of measures, we also included search terms related to validity, reliability, and diagnostic accuracy (i.e., alpha, kappa, sensitivity, specificity, positive predictive value, negative predictive value). Search terms included MeSH headings related to our research question, general terms related to substance use and psychometric properties or interest, as well as specific terms referencing the names of well-known substance use measures. The search terms used are provided in the appendix. This review was registered in Prospero, the International prospective register of systematic reviews (study number: CRD42017058813).

### Primary outcomes

We aimed to estimate the pooled summary estimates for the following psychometric outcomes: Cronbach’s alpha, kappa, sensitivity, specificity, positive predictive value, and negative predictive value. We recognize that there are a number of measure characteristics that relate to validity [32]. However, to focus our review and facilitate the feasibility of completing this study, we have decided to restrict the scope of our validity measures to Cronbach’s alpha. Descriptions for these outcomes are provided below:

**Table:** 

Psychometric Outcome	Description
Cronbach’s alpha	measure of internal consistency, that is, how closely correlated a set of scale items are, as a group.
Kappa	measure of inter-rater agreement or inter-rater reliability for qualitative (categorical) items which takes into account the possibility of the agreement occurring by chance.
Sensitivity	measure of a test/scales’ ability to correctly detect patients who do truly have the condition (i.e., proportion of people who screen positive for substance use disorders according to the scale, among those who truly have substance use disorders based on an established standard (“gold standard”) such as meeting diagnostic criteria for a disorder).
Specificity	measure of the test/scales’ ability to correctly detect patients without a condition (i.e., proportion of people who screen negative for substance use disorders according to the scale, among those who truly do not have substance use disorders based on an established standard such as meeting diagnostic criteria for a disorder).
Positive predictive value (PPV)	the probability that persons with a positive screening result actually has the disorder. (i.e., proportion of people who meet diagnostic criteria for a substance use disorder among those who screened positive for the disorder on a scale).
Negative predictive value (NPV)	the probability that people with a negative screening test actually do not have the disease. (i.e., proportion of people who meet diagnostic criteria for a substance use disorder among those who screened negative for a substance use disorder in a scale).

### Eligibility criteria

We searched for relevant publications that met all of the following inclusion criteria: 1) studies that reported one or more of the psychometric outcomes of interest; 2) studies that examined on one or more substance use measures related to our substance classes of interest (i.e., alcohol, methamphetamine and amphetamine, cocaine, heroin, and ecstasy) or for substance use in general (i.e., some measures do not differentiate between multiple substances or assess classes of substances all together); 3) publication written in English (note: studies that administered measures that were not in English were eligible as long as the publication was written in English) .

We excluded publications using the following exclusion criteria: 1) reporting insufficient information on reliability, validity and diagnostic accuracy for substance use measures/assessments (i.e., no numeric information on our psychometric outcomes, sample size); 2) articles that provide psychometric data for a measure/assessment that is not related to substance use (e.g., a study on internal consistency data on a depression scale among substance users); 3) articles and/or secondary data analyses that report reliability and validity data from a primary outcome paper that was already included in the review; 4) reviews, commentaries, case report studies and other publications with insufficient reporting of data; 5) substance use measures/assessments that focus on aspects other than actual substance consumption, dependence or substance use disorder (e.g., a study reporting validity of a self-efficacy scale for resisting substance use; a study that examines the underlying mechanisms of substance use among those who already have a substance use disorder); and 6. studies with psychometric properties that focus on substance classes outside the scope of our review (e.g. marijuana or tobacco).

### Screening procedures

All citations (including their titles and abstracts) captured by the search strategy were imported into Covidence.org (Melbourne Victoria), which allowed research team members to independently review and screen citations using a centralized, online database. Each title/abstract was screened by two members of a team comprising master-, doctoral-, and post-doctoral-level researchers trained in the study protocol (co-authors PP, DH, RC, DS, CM, PM, and FC) and citations that were coded as eligible by both reviewers were moved to the full-text review phase. The same process was then repeated for full-text articles. In the event of discrepancies between reviewers in both the title and abstract phase and the full-text phase, a third team member (GMS) reviewed the relevant documents and helped reconcile the differences. Articles that were deemed eligible in the full-text review stage were included in the data extraction phase described below.

### Data extraction

Team members extracted data on the psychometric properties, scale and study characteristics, sample size, study sample characteristics/co-factors of interest (country where study was conducted, number of sites, language that the scale was administered, gender of participants included), cut-offs used, comparison measure/gold-standard used, and other information relevant to study, including information on study quality [33]. Some papers reported multiple data points for psychometric outcomes from different study populations (e.g., disaggregated data by sex or different research sites). These data points were extracted as separate records only if the paper did not provide a single overall measure for the psychometric outcomes for the entire study sample, consistent with other analyses [24].

### Assessment of bias risk

For studies reporting diagnostic measures (e.g., sensitivity and specificity), reviewers rated study quality using the Revised Tool for the Quality Assessment of Diagnostic Accuracy Studies, QUADAS-2, guidelines [33], which includes quality rating questions on the study’s patient selection, index test, reference standard, and flow and timing. For studies that did not include diagnostic accuracy measures, only relevant domains of QUADAS-2 were assessed, as appropriate (i.e., rating regarding the reference standard was not conducted). All extracted data were entered into an electronic questionnaire programmed in Qualtrics, and checked by another researcher (conducted by the same co-authors who screened citations, as well as co-author BK) to verify accuracy.

### Data analyses

We calculated separate pooled summary estimates for each of the 37 substance use measures and also fitted separate models for each of the six psychometric outcomes for validity, reliability, and accuracy. For alpha, kappa, PPV and NPV, we pooled data across studies using DerSimonian-Laird random effects models, implemented in STATA version 13 (Colleges Station, TX) [34]. Random effects meta-analyses models, as opposed to fixed-effects models, are preferred for pooling data from diagnostic accuracy tests since heterogeneity is presumed to exists across these studies [35]. Random effects models, which are considered the default models used in meta-analyses for diagnostic accuracy tests, synthesize the psychometric outcomes from separate studies into a weighted average effect size (pooled summary estimate), using inverse variance weighting, based on sample size, while taking into account the extent of the variability of the effect sizes observed in separate studies [35]. Additionally, for sensitivity and specificity, we used hierarchical logistic regression models, implemented using the metandi command in STATA, to account for the correlation between the two measures (i.e., trade-off between sensitivity and specificity) [36–38]. Since metandi requires a minimum of four observations to conduct a meta-analysis, we pooled measures with less than four records for sensitivity and specificity outcomes using the random effects models described for other outcomes, and noted this alternate approach in the results, as appropriate.

### Classification and evaluation of pooled estimates

Qualitatively, pooled summary estimates for alpha and kappa were classified as “excellent” for estimates that were > 0.89, “good” for estimates that were between 0.85–0.89, “moderate” for estimates that were between 0.80–0.84, “fair” for estimates that were between 0.75–0.79, or “unsatisfactory” for estimates below 0.75, consistent with other studies [24, 39].

Pooled summary estimates for sensitivity, specificity, positive predictive value and negative predictive value were classified as “excellent” for estimates that were > 0.89, “good” for estimates that were between 0.8–0.89, “moderate” for estimates that were between 0.6–0.79, and “low” for estimates that were < 0.6 [24, 40].

For each pooled psychometric summary estimate, we calculated I^2^ statistics, which represents the percentage of total variation across studies, to assess heterogeneity. We considered pooled estimates as having low heterogeneity if I^2^ 25%, moderate heterogeneity if I^2^ 50%, and high heterogeneity if I^2^ 75% [41]. We did not use standard meta-analyses tests for publication bias given the limitations of these tests for diagnostic test accuracy studies and due to the characteristics of our psychometric outcomes (e.g., truncated measures cannot fall below zero) [42]. As indicated in the Cochrane Handbook for Systematic Reviews of Diagnostic Test Accuracy, using these tests are inappropriate because they will likely lead to a high false-positive rate for publication bias [35].

## Results

### Screening and study inclusion

**Fig. 1:**
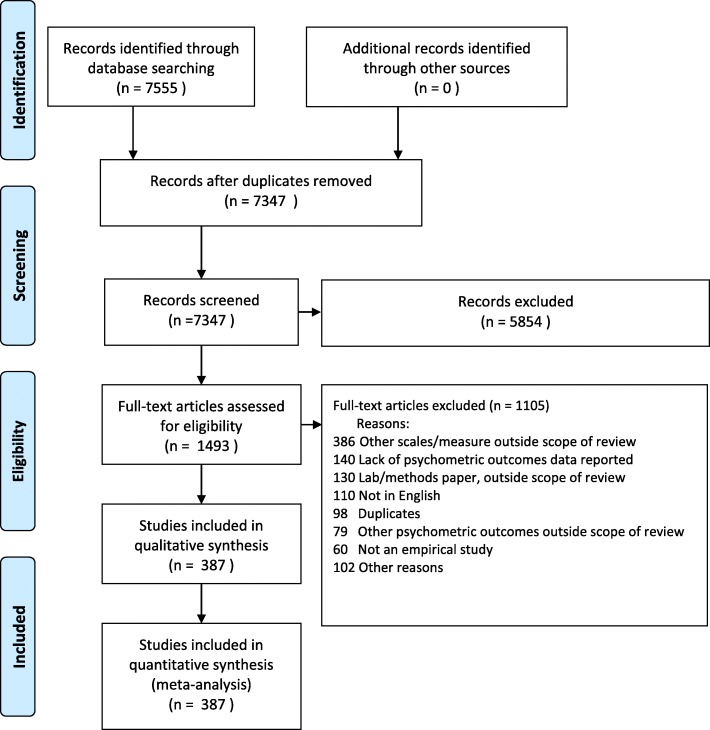
Study Identification, Screening, Eligibility, and Inclusion for Meta-Analysis

Study screening and inclusion is summarized in Fig. 1. In brief, in the identification stage, we initially identified 7555 references in the initial search, of which, 208 were excluded for being duplicates. In the title and abstract review phase, reviewers excluded 5854 studies that were deemed ineligible. Full-text reviews were conducted for 1493 articles that were deemed eligible from title and abstract review. Of the full-text reviewed articles, 1105 studies were excluded for not meeting eligibility criteria. The most common reasons for exclusion were: scales or measures that were outside the scope of review (*n* = 386), lack of psychometric data on scales of interests (*n* = 140), lab or methods papers that were outside the scope of the review (*n* = 130), non-English language publications (*n* = 110), duplicate study (*n* = 98), psychometric outcomes that were outside the scope of review (*n* = 79). In total, there were 387 unique studies included in the data extraction phase containing sufficient data on the outcomes for 37 scales (**Table **1).

**Table 1:** Substance use Measures/Scales identified in Systematic Review and Meta-analyzed

Scale Name Full	Scale Abbreviation	No. of Unique Studies^**a**^	Description
**SELF-REPORTED MEASURES**			
Alcohol Dependence Scale	ADS	3	The Alcohol Dependence Scale (ADS) is an alcohol screening and assessment tool that provides a quantitative index for the severity of alcohol dependence. Developed with respect to the concept of alcohol dependence syndrome, the ADS is comprised of 25 items that assess withdrawal symptoms, alcohol tolerance, awareness of dependence, ability to control drinking, and the salience of drink-seeking behavior.
Addiction Severity Index	ASI	4	The Addiction Severity Index (ASI) is a structured interview for assessing alcohol and drug dependence. The ASI comprises 200 items across seven scales assessing past 30-day and lifetime alcohol use, drug use, medical problems, employment/support problems, legal problems, family/social problems, and psychological problems.
Addiction Severity Index-Alcohol (alcohol sub-scale)	ASI-A	22	The Addiction Severity Index – Alcohol (ASI-A) is the alcohol sub-scale of the Addiction Severity Index. It assesses frequency of past 30-day and lifetime alcohol use and intoxication, alcohol-related problems including withdrawal symptoms, and treatment experiences.
Addiction Severity Index-Drugs (drugs sub-scale)	ASI-D	20	The Addiction Severity Index – Drugs (ASI-D) is the drug sub-scale of the Addiction Severity Index. It assesses frequency of past 30-day and lifetime use of 10 drug classes (heroin, methadone, other opiates/analgesics, barbiturates, other sedatives/hypnotics/tranquilizers, cocaine, amphetamines, cannabis, hallucinogens, and inhalants), drug-related problems including overdose, and treatment experiences.
The Alcohol, Smoking, and Substance Involvement Screening Test	ASSIST	8	The Alcohol, Smoking and Substance Involvement Screening Test (ASSIST) is a tool developed by the World Health Organization to screen for risky use of alcohol, tobacco and other drugs. It comprises items measuring past three-month and lifetime frequency of use of tobacco products, alcohol and illicit drugs (cannabis, cocaine, amphetamine-type stimulants, inhalants, sedatives/sleeping pills, hallucinogens, opioids, other drugs) and related health, social, legal and financial problems.
Alcohol Use Disorders Identification Test	AUDIT	127	The Alcohol Use Disorders Identification Test (AUDIT) is a ten-question test developed by a World Health Organization-sponsored collaborative project to identify persons with hazardous and harmful patterns of alcohol consumption or alcohol dependence. It comprises questions on amount and frequency of alcohol consumed, alcohol dependence, and alcohol-related problems.
Alcohol Use Disorders Identification Test - Question 3	AUDIT-3	16	The Alcohol Use Disorders Identification Test – 3 (AUDIT-3) is a brief alcohol screening instrument. Derived from the third question of the ten-item AUDIT developed by the World Health Organization, it consists of a single-item measure assessing heavy episodic drinking.
Alcohol Use Disorders Identification Test - C	AUDIT-C	42	The Alcohol Use Disorders Identification Test - Concise (AUDIT-C) is a brief alcohol screening instrument derived from the first three questions of the ten-item AUDIT developed by the World Health Organization. It assesses frequency of alcohol consumption, number of standard drinks consumed on a typical drinking day, and frequency of consumption of six or more drinks on one occasion.
Brief Michigan Alcoholism Screening Test	B-MAST	12	Adapted from the original Michigan Alcohol Screening Test (MAST), the Brief Michigan Alcohol Screening Test (B-MAST) is a shortened 10-item alcohol use questionnaire that aims to identify alcohol dependence. The 10 items assess the presence or absence of negative consequences as a result of drinking through yes or no self-reported responses.
Cut down, Annoyed, Guilty, Eye-opener	CAGE	98	CAGE is a four-item measure designed to identify problem drinking. The four items assess whether an individual has ever felt the need to cut down on their drinking, been annoyed by others’ criticism of their drinking, felt guilty about drinking, or felt the need to have a drink first-thing in the morning to steady their nerves or get rid of a hangover.
Composite International Diagnostic Interview	CIDI	Original version: 2; Version 2.1: 2; Version 3: 2	The Composite International Diagnostic Interview (CIDI) is a structure interview developed by the World Health Organization to assess psychiatric disorders based on International Statistical Classification of Diseases and Related Health Problems (ICD) definitions and Diagnostic and Statistical Manual of Mental Disorders (DSM) criteria.
Car, Relax, Alone, Forget, Friends, Trouble	CRAFFT	11	The Car, Relax, Alone, Forget, Friends, Trouble (CRAFFT) questionnaire is screening tool to identify substance use problems among adolescents.
Drug Abuse Screen Test	DAST	10	The Drug Abuse Screen Test (DAST) is a 28-item questionnaire parallel to those of the Michigan Alcoholism Screening Test (MSM) to screen for drug use problems and consequences.
Drug Abuse Screen Test – 10 item	DAST-10	8	The Drug Abuse Screen Test – 10 item (DAST-10) is a shorten version of the DAST screening test. It is used to assess problems and consequences related to substance use.
Drug Use Disorders Identification Test	DUDIT	12	The Drug Use Disorders Identification Test (DUDIT), designed as a parallel instrument to the AUDIT, is an 11-item self-administered screening instrument for drug-related problems. It assesses use patterns of use and various drug-related problems.
Michigan Alcohol Screening Test	MAST	22	The Michigan Alcohol Screening Test (MAST) is a 25-item scale designed to assess lifetime symptoms of alcoholism with a focus on late-stage symptoms.
Problem Oriented Screening Instrument for Teenagers	POSIT	3	The Problem Oriented Screening Instrument for Teenagers (POSIT) is a self-administer screening questionnaire comprised of 139 items which screen for potential problems in 10 domains including Substance use and abuse; Physical health; Mental health; Family relations; Peer relations; Educational status; Vocational status; Social skills; Leisure/recreation; and Aggressive behavior.
Self-Administered Alcoholism Screening Test	SAAST	4	The Self-Administered Alcoholism Screening Test (SAAST) is a 35-item questionnaire to screen for alcohol dependence. It assesses problem related to alcohol in the following domains: loss of control, occupational and social disruption, physical consequences, emotional consequences, concern on the part of others, and family members with alcohol problems.
Semi-Structured Assessment for Drug Dependence and Alcoholism	SSADDA	1	The Semi-structured Assessment for Drug Dependence and Alcoholism (SSADDA) is a screening instrument that assesses alcohol/drug abuse and dependence as well as other DSM-IV disorders throughout the lifetime. It was developed from the Semi-Structured Assessment for the Genetics of Alcoholism, and therefore includes questions on the onset and recency of individual alcohol/drug abuse and dependence symptoms, allowing temporal assessment of symptom clusters. Its format as a semi-structured interview lists questions to be read verbatim, but also allows the interviewer to add follow-up questions.
Severity of Dependence	SDS	9	The Severity of Dependence Scale (SDS) is a 5-item questionnaire used to measure the degree of dependence on different classes of drugs, with a focus on the psychological components of dependence.
Tolerance-Annoyance Cut Down Eye Opener	TACE	9	Tolerance-Annoyance Cut Down Eye Opener (TACE) is a 4-item screening tool to identify maternal prenatal problematic alcohol use.
Timeline Followback	TLFB	5	The Timeline Followback (TLFB) is a method that involves the use of a timeline (e.g., calendar) to ask individuals to estimate their daily alcohol and/or drug use consumption retrospectively (e.g., 7 days, 2 years).
Tolerance, Worried, Eye-Opener, Amnesia, Cut down	TWEAK	17	Originally developed as an alcohol screening tool for periconceptional risk in obstetric outpatients, TWEAK, is a 5-item questionnaire that seeks to identify harmful drinking. The 5-items are comprised of questions from the MAST, CAGE, and T-ACE screening tools and cover tolerance, worry, eye-opener, amnesia, k/cut-down (which make up the acronym TWEAK). TWEAK is primarily used as an efficient method to determine whether or not the risk or presence of harmful drinking should be further assessed and/or treated.
The Chemical Use, Abuse, and Dependence	CUAD	1	The Chemical Use, Abuse, and Dependence (CUAD) is a semi-structure interview that can measure substance use severity and substance use disorders.
**BIOMARKERS**			
% Carbohydrate deficient transferrin	%CDT	35	Carbohydrate-deficient transferrin (CDT) is an alcohol biomarker that is used as a clinical screening and monitoring tool to identify heavy drinking. Transferrin is glycoprotein produced in the liver that normally has 3–5 carbohydrate side chains. Heavy alcohol use, however, inhibits the enzymes involved to appropriately regulate these side chains; causing the transferrin to be carbohydrate deficient. A %CDT reading ≥2.6 indicates that a participant may have had on average at least 5 alcoholic drinks daily for ≥2 weeks. Laboratory blood test can detect elevated levels of CDT (%CDT), which are indicative of heavy alcohol consumption and often used to detect relapses.
Alanine transaminase	ALT	26	Alanine aminotransferase (ALT) is a biomarker, which indicates liver damage from different types of disease and conditions. It that is used as a clinical screening and monitoring tool to check for chronic alcohol use. ALT is an enzyme found mostly in the cells of the liver and kidney. When the liver is damaged, ALT is released into the blood. Elevated ALT in laboratory tests is indicative of heavy alcohol consumption and often used to detect relapses.
Aspartate transaminase	AST	31	Aspartate transaminase (AST) is a biomarker, which indicates liver damage from different types of disease and conditions. It that is used as a clinical screening and monitoring tool to check for chronic alcohol use. The concentrations of AST in the serum are normally low. However, if the liver is damaged, the liver cell (hepatocyte) membrane becomes more permeable and some of the enzymes leak out into the blood circulation. Elevated AST in laboratory tests are indicative of chronic alcohol abuse
Aspartate transaminase, Alanine transaminase ratio	AST/ALT	5	AST and ALT are considered to be two of the most important tests to detect liver injury. The ALT: AST ratio is normally and in other condition is less than 1, but becomes greater than unity during liver injury. Elevated AST/ALT in laboratory tests are indicative of chronic alcohol abuse.
Blood alcohol concentration	BAC	5	Blood Alcohol Concentration (BAC) levels represent the percent of your blood that is concentrated with alcohol. It is most commonly used as a metric of alcohol intoxication for legal or medical purposes. Its primary goal is to determine if alcohol has been consumed.
Carbohydrate deficient transferrin	CDT	9	Carbohydrate-deficient transferrin (CDT) is an alcohol biomarker that is used as a clinical screening and monitoring tool to identify heavy drinking. Transferrin is glycoprotein produced in the liver that normally has 3–5 carbohydrate sidechains. Heavy alcohol use, however, inhibits the enzymes involved to appropriately regulate these sidechains; causing the transferrin to be carbohydrate deficient. Laboratory blood test can detect elevated levels of CDT (%CDT), which are indicative of heavy alcohol consumption and often used to detect relapses.
CDTech	CDTech	35	Description: CDTect is a common method of using carbohydrate-deficient transferrin (CDT) to screen for heavy alcohol use.
Carbohydrate deficient transferrin + Mean corpuscular volume	CDT + MCV	5	Carbohydrate-deficient transferrin (CDT) and Mean corpuscular volume (MCV) are two biomarkers commonly used to screen for heavy drinking. MCV is the average volume of blood cells, which increase in size after 4 to 8 weeks of excessive alcohol intake. CDT is transferrin, a glycoprotein produced in the liver that has become carbohydrate deficient. Heavy alcohol use prevents enzymes from properly regulating the carbohydrate side chains in transferrin, thus increasing the value of carbohydrate-deficient transferrin. Using the combined biomarkers of CDT and MCV, a patient must exceed the cut-off of both biomarkers to be screened positive.
Gamma-Glutamyl Transferase	GGT	68	Gamma-Glutamyl Transferase (GGT) is an enzyme that when elevated in serum is reflective of liver damage. Subsequently, clinical laboratory GGT tests are commonly used to detect and monitor excessive alcohol consumption. Elevated GGT levels typically correspond with continuous and chronic alcohol abuse as opposed to episodic heavy drinking.
Gamma-Glutamyl Transferase + Mean corpuscular volume	GGT + MCV	10	Gamma-Glutamyl Transferase (GGT) and Mean corpuscular volume (MCV) are two biomarkers commonly used in screening heavy alcohol intake. GGT is a type of enzyme that, when elevated in serum, is reflective of liver damage. MCV is the average volume of red blood cells, which increases after 4 to 8 weeks of excessive drinking. Used in combination, a patient must exceed the cut-offs for both GGT and MCV in order to be screened positive.
Ethyl glucuronide	EtG	5	Ethyl glucuronide (EtG) is a byproduct of the body’s metabolization of alcohol, and can be detected in the hair for up to 90 days. Compared to a blood or urine analysis, a hair analysis for EtG provides a much longer window of detection for heavy alcohol consumption.
Mean corpuscular volume	MCV	51	Mean corpuscular volume (MCV) is the average volume of red blood cells, measured by multiplying a volume of blood by the proportion of the blood that is cellular, and then dividing the product by the number of red blood cells within that sample. The size of red blood cells increase after 4 to 8 weeks of excessive alcohol intake, making MCV effective as an alcohol biomarker. MCV is not very sensitive as a standalone measure or specific in detecting alcohol relapse, as it is slow to return to a normal value. It is, however, an easy and affordable method of testing.
Phosphatidylethanol	PEth	8	Phosphatidylethanol (PEth), a commonly used alcohol biomarker, is an abnormal group of phospholipids that are formed in red blood cells only in the presence of alcohol. Clinical laboratory tests can identify the presence of PEth in blood, which is indicative of alcohol abuse. PEth testing is a popular detection tool for heavy alcohol consumption because it is considered a direct biomarker for ethanol and has 99% sensitivity.

Note:^**a**^Some studies contributed more than one data point/were comprised of more than on study populations. References for studies, by scale/measure, are presented in Supplementary Table 2

### Study characteristics

Table 2 presents characteristics of the studies included in this meta-analysis. As mentioned, studies published in English were included in this review, regardless of the language in which the scales were administered. Among the 387 studies included, the most those common language in which the scale/measure was conducted in was English (63%), followed by Spanish (9%), French (5%), Portuguese (3%), and Chinese (2%). A large proportion of studies were conducted in the United States (40%). The median sample size was 286 [Range = 9–50,049]. The vast majority of studies (83%) included men and women (*n* = 323). Additionally, 11% (*n* = 42) of the studies included study sample comprised only of men, while 5% (*n* = 20) studies included study samples comprised only of women. Most studies were published after 1999 (66%), with studies published between 2000 and 2009 accounting for 38% (*n* = 148) of the studies meta-analyzed, and studies published between 2010 and 2017 accounting for 28% (*n* = 110). Most studies involved a single study site 61%, while 39% were multi-site studies. Additionally, 72% of the studies involved convenience samples, 20% included random or probability based samples, and 7% had other or unclear sampling strategies.

**Table 2:** Pooled Summary Estimates

**Self-Reported** Scale/Measure	Scale Abbreviation	Pooled Alpha; 95%CI				Pooled Kappa; 95%CI				Pooled Sensitivity; 95%CI				Pooled Specificity; 95%CI				Pooled PPV; 95%CI				Pooled NPV; 95%CI			
Alcohol Dependence Scale	ADS	0.897	0.800	–	0.993	ID		–		0.908	0.822	–	0.955	0.794	0.671	–	0.879	ID		–		ID		–	
Addiction Severity Index	ASI	0.837	0.809	–	0.866	ID		–		ID		–		ID		–		ID		–		ID		–	
Addiction Severity Index-Alcohol (alcohol sub-scale)	ASI-A	0.773	0.732	–	0.814	ID		–		0.833	0.670	–	0.924	0.792	0.670	–	0.877	ID		–		ID		–	
Addiction Severity Index-Drugs (drugs sub-scale)	ASI-D	0.684	0.627	–	0.742	ID		–		0.839	0.753	–	0.899	0.854	0.766	–	0.913	ID		–		ID		–	
The Alcohol, Smoking, and Substance Involvement Screening Test	ASSIST	0.854	0.804	–	0.905	ID		–		0.834	0.799	–	0.868	0.726	0.570	–	0.882	ID		–		ID		–	
Alcohol Use Disorders Identification Test	AUDIT	0.847	0.829	–	0.865	0.461	0.253	–	0.669	0.860	0.838	–	0.879	0.872	0.854	–	0.888	0.610	0.510	–	0.710	0.940	0.927	–	0.953
Alcohol Use Disorders Identification Test-3	AUDIT-3	NA		–		ID		–		0.844	0.800	–	0.879	0.835	0.750	–	0.895	0.631	0.491	–	0.771	0.940	0.902	–	0.979
Alcohol Use Disorders Identification Test-C	AUDIT-C	0.748	0.695	–	0.802	0.410	0.386	–	0.433	0.874	0.843	–	0.900	0.842	0.811	–	0.870	0.496	0.390	–	0.602	0.878	0.834	–	0.923
Brief Michigan Alcoholism Screening Test	B-MAST	ID		–		ID		–		0.502	0.381	–	0.622	0.971	0.956	–	0.981	0.653	0.376	–	0.930	0.902	0.866	–	0.937
Cut down, Annoyed, Guilty, Eye-opener	CAGE	0.698	0.650	–	0.747	0.574	0.337	–	0.811	0.705	0.664	–	0.742	0.898	0.882	–	0.913	0.513	0.446	–	0.581	0.907	0.881	–	0.932
Composite International Diagnostic Interview, original	CIDI	NA		–		0.815	0.610	–	1.020	ID				ID				ID				ID			
Composite International Diagnostic Interview,version 2.1	CIDI, version 2.1	NA				ID				0.749	0.686	–	0.812	0.842	0.689	–	0.995	ID				ID			
Composite International Diagnostic Interview, version 3	CIDI, version 3	NA				ID				0.913	0.816	–	1.010	0.990	0.985	–	0.995	0.914	0.871	–	0.958	0.990	0.985	–	0.995
Car, Relax, Alone, Forget, Friends, Trouble	CRAFFT	0.687	0.639	–	0.735	ID		–		0.897	0.837	–	0.937	0.764	0.675	–	0.834	0.571	0.343	–	0.799	0.857	0.449	–	1.00
Drug Abuse Screen Test	DAST	0.937	0.927	–	0.947	0.832	0.577	–	1.0	0.847	0.740	–	0.915	0.844	0.679	–	0.932	0.510	0.323	–	0.698	0.946	0.892	–	0.999
Drug Abuse Screen Test - 10 item	DAST-10	0.785	0.678	–	0.892	ID		–		0.902	0.748	–	0.966	0.81 8	0.716	–	0.889	0.803	0.695	–	0.910	0.864	0.813	–	0.914
Drug Use Disorders Identification Test	DUDIT	0.922	0.895	–	0.950	ID		–		0.932	0.887	–	0.960	0.789	0.672	–	0.872	0.606	0.342	–	0.869	0.915	0.822	–	1.00
Michigan Alcohol Screening Test	MAST	0.822	0.780	–	0.863	0.693	0.578	–	0.808	0.705	0.583	–	0.804	0.853	0.774	–	0.908	0.507	0.302	–	0.713	0.878	0.816	–	0.940
Problem Oriented Screening Instrument for Teenagers	POSIT	0.857	0.730	–	0.984	0.881	0.817	–	0.945	0.842	0.722	–	0.962	0.823	0.745	–	0.902	ID		–		ID		–	
Self-Administered Alcoholism Screening Test	SAAST	0.889	0.791	–	0.987	ID		–		0.522	0.331	–	0.714	0.825	0.755	–	0.896	0.320	0.219	–	0.422	0.917	0.890	–	0.945
Semi-Structured Assessment for Drug Dependence and Alcoholism	SSADDA	ID		–		0.837	0.767	–	0.907	ID		–		ID		–		ID		–		ID		–	
Severity of Dependence	SDS	0.855	0.780	–	0.931	ID		–		0.830	0.759	–	0.901	0.837	0.782	–	0.892	0.897	0.856	–	0.937	0.826	0.761	–	0.891
Tolerance-Annoyance Cut Down Eye Opener	TACE	0.495	0.466	–	0.524	ID		–		0.827	0.737	–	0.918	0.722	0.651	–	0.793	0.349	0.245	–	0.453	0.867	0.622	–	1.0
Timeline Followback	TLFB	NA		–		0.857	0.807	–	0.907	0.798	0.725	–	0.872	0.965	0.945	–	0.986	ID		–		ID		–	
Tolerance, Worried, Eye-Opener, Amnesia, Cut down	TWEAK	0.619	0.551	–	0.688	ID		–		0.850	0.797	–	0.890	0.864	0.820	–	0.898	0.434	0.263	–	0.606	0.880	0.704	–	1.0
The Chemical Use, Abuse, and Dependence	CUAD	0.962	0.940	–	0.983	ID		–		ID		–		ID		–		ID		–		ID		–	
**Biomarkers**	Abbreviation	Pooled **Alpha**; 95%CI				Pooled **Kappa**; 95%CI				Pooled **Sensitivity**; 95%CI				Pooled **Specificity**; 95%CI				Pooled **PPV**; 95%CI				Pooled **NPV**; 95%CI			
% Carbohydrate deficient transferrin	%CDT	NA				NA		–		0.565	0.471	–	0.654	0.913	0.884	–	0.936	0.580	0.377	–	0.783	0.850	0.782	–	0.918
Alanine transaminase	ALT	NA		–		NA		–		0.316	0.239	–	0.403	0.882	0.830	–	0.920	0.372	0.183	–	0.560	0.632	0.417	–	0.846
Aspartate transaminase	AST	NA		–		NA		–		0.476	0.400	–	0.552	0.865	0.814	–	0.903	0.420	0.273	–	0.567	0.693	0.554	–	0.833
Aspartate transaminase, Alanine transaminase ratio	AST/ALT	NA		–		NA		–		0.340	0.220	–	0.460	0.728	0.519	–	0.937	ID		–		ID		–	
Blood alcohol concentration	BAC	NA		–		NA		–		0.638	0.589	–	0.686	0.795	0.719	–	0.871	0.596	0.145	–	1.0	0.687	0.518	–	0.856
Carbohydrate deficient transferrin	CDT	NA		–		NA		–		0.585	0.425	–	0.728	0.959	0.932	–	0.976	0.853	0.740	–	0.966	0.790	0.727	–	0.854
CDTech	CDTech	NA		–		NA		–		0.537	0.450	–	0.622	0.895	0.876	–	0.911	0.518	0.367	–	0.669	0.795	0.614	–	0.975
Carbohydrate deficient transferrin + Mean corpuscular volume	CDT + MCV	NA		–		NA		–		0.742	0.599	–	0.884	0.928	0.908	–	0.947	0.741	0.509	–	0.974	0.917	0.826	–	1.00
Gamma-Glutamyl Transferase	GGT	NA		–		NA		–		0.571	0.502	–	0.638	0.827	0.782	–	0.865	0.430	0.351	–	0.509	0.819	0.701	–	0.937
Gamma-Glutamyl Transferase + Mean corpuscular volume	GGT + MCV	NA		–		NA		–		0.641	0.377	–	0.840	0.870	0.759	–	0.935	0.470	0.275	–	0.664	0.880	0.813	–	0.948
Ethyl glucuronide	EtG	NA		–		NA		–		0.825	0.606	–	0.936	0.953	0.896	–	0.979	0.614	0.389	–	0.840	0.857	0.779	–	0.935
Mean corpuscular volume	MCV	NA		–		NA		–		0.390	0.330	–	0.453	0.905	0.878	–	0.927	0.476	0.362	–	0.589	0.794	0.728	–	0.860
Phosphatidylethanol	PEth	NA		–		NA		–		0.871	0.787	–	0.956	0.939	0.905	–	0.973	ID		–		ID		–	

Note: For biomarker measures, alpha is not calculated, therefore this estimate is not applicable (NA). Measures with less than 2 studies have ID for meta-analyses (ID)

### Assessment of bias in study quality

**Fig. 2:**
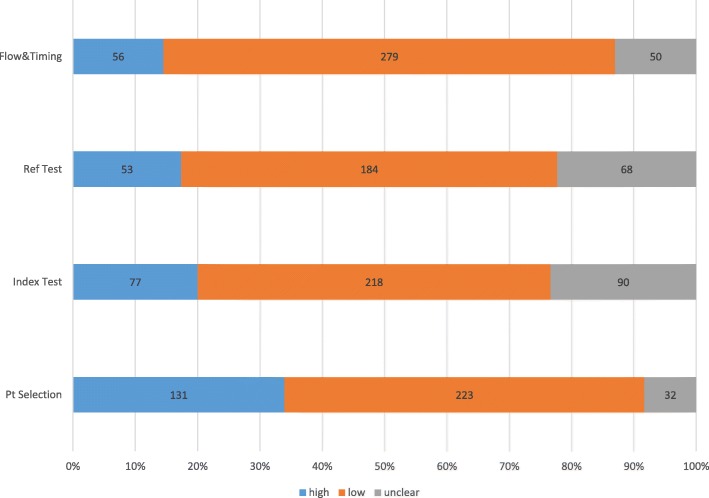
Overall Summary of study quality ratings from the Revised Tool for the Quality Assessment of Diagnostic Accuracy Studies, QUADAS-2

The risk of bias in the four QUADAS 2 domains for each study included in this meta-analysis is presented in **Supplementary Table** **1****.** The distribution of the QUADAS 2 domains for the entire study is summarized in Fig. 2. Of the studies included, 58% of studies had a low risk of bias with respect to the patient population; 57% has low risk of bias in the index test domain, 48% has low risk of bias in the reference standard test domain, and 72% had low risk for the flow and timing. Overall, only 16% of studies had low risk of bias across all four of these QUADAS 2 domains.

### Pooled summary estimates: overall findings

**Fig. 3:**
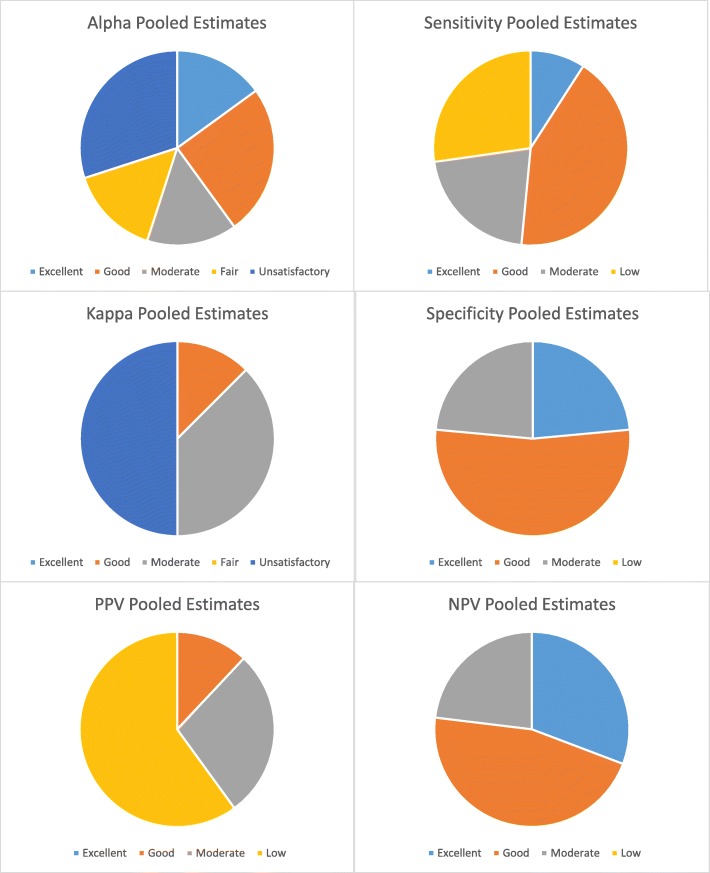
Distribution of Pooled Summary Estimates of Psychometric Outcomes

The pooled summary estimates of psychometric properties of substance use measures (which are described in Table 1) are quantitatively and qualitatively summarized in Tables 2 and 3, respectively. Overall, 65% of pooled estimates for alpha were in the range of fair-to-excellent; 44% of estimates for kappa were in the range of fair-to-excellent. In addition, 69, 97, 37 and 96% of pooled estimates for sensitivity, specificity, positive predictive value, and negative predictive value, respectively, were in the range of moderate-to-excellent **(**Fig. 3**)**.

**Table 3:**
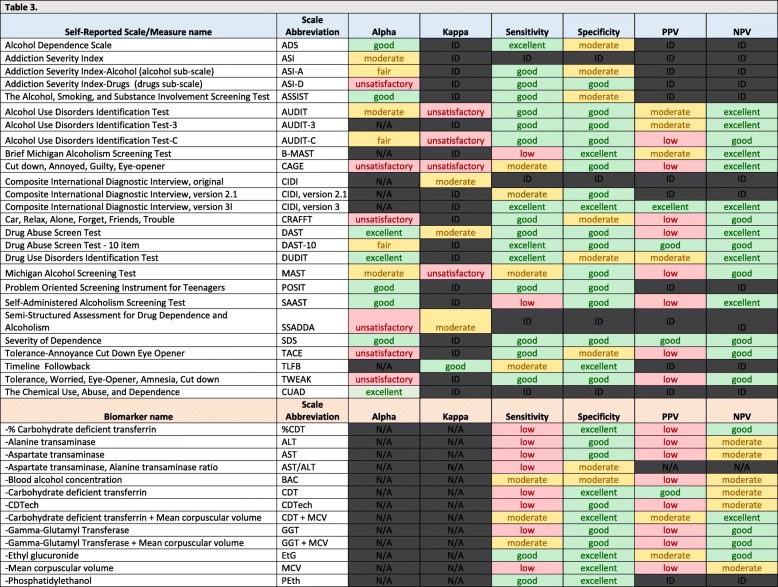
Qualitative Interpretation of Pooled Estimates

Notes: Pooled summary estimates for alpha and kappa were classified as “excellent” for estimates that were > 0.89, “good” for estimates that were between 0.85–0.89, “moderate” for estimates that were between 0.80–0.84, “fair” for estimates that were between 0.75–0.79, or “unsatisfactory” for estimates below 0.75, consistent with other studies [24, 39]. Pooled summary estimates for sensitivity, specificity, positive predictive value and negative predictive value were classified as “low” for estimates that were < 0.6, “moderate” for estimates that were between 0.6–0.79, “good” for estimates that were between 0.8–0.89 and “excellent” for estimates that were > 0.89 [24, 40]. N/A = not applicable. ID = Insufficient Data

Self-reported measures that had all pooled estimates that were fair/moderate or better include the following: Alcohol Dependence Scale; Addiction Severity Index (ASI); ASI subscale for Alcohol; ASSIST; the Composite International Diagnostic Interview, including the original version, as well as version 2.1 and version 3; Drug Abuse Screen Test - 10 item scale; Drug Use Disorders Identification Test; Problem Oriented Screening Instrument for Teenagers; Severity of Dependence scale; Timeline Followback; and Chemical Use, Abuse, and Dependence. Biomarkers that had all pooled estimates that were fair/moderate or better include the following: Ethyl glucuronide; Phosphatidylethanol test; and the combined used of Carbohydrate deficient transferrin and Mean corpuscular volume. In general, we also observed high heterogeneity between studies for most pooled estimates.

### Pooled summary estimates, by substance use measure

The pooled estimates and 95% confidence intervals for alpha, kappa, sensitivity, specificity, positive predictive value, and negative predictive value are shown in Table 2**,** respectively. Below we summarize the results of the pooled summary estimates alphabetically for each of the 37 substance use measures, grouping self-reported measures and biomarkers separately. The list of references for the studies meta-analyzed for each scale/measure is presented in **Supplementary Table****2**.

#### Self-reported measures

##### Alcohol dependence scale (ADS)

The pooled alpha estimate for ADS (3 data points) was good: 0.90 (95%CI = 0.80–0.99) and there was high heterogeneity between studies (I^2^ 98.9%). The pooled sensitivity estimate for ADS (2 data points) was excellent: 0.95 (95%CI = 0.90–1.00) and there was low heterogeneity between studies (I^2^ 0%). The pooled specificity estimate (2 data points) was moderate: 0.64 (95%CI = 0.52–0.77) and there was moderate heterogeneity between studies (I^2^ 60.1%). There was insufficient data to calculate the pooled PPV and NPV estimates for ADS.

##### Addiction Severity Index (ASI)

The pooled alpha estimate for ASI (3 data points) was good: 0.84 (95%CI = 0.81–0.87) and there was moderate heterogeneity between studies (I^2^ 38.5%). There was insufficient data to calculate pooled kappa, sensitivity, specificity, PPV, and NPV estimates.

##### Addiction severity index-alcohol (alcohol sub-scale; ASI-A)

The pooled alpha estimate (18 data points) was moderate: 0.77 (95%CI = 0.73–0.81) and there was high heterogeneity between studies (I^2^ 94.3%). The pooled sensitivity estimate for ASI-A (6 data points) was good: 0.83 (95%CI = 0.67–0.92) and there was high heterogeneity between studies (I^2^ 87.6%). The pooled specificity estimate for ASI-A (6 data points) was moderate: 0.79 (95%CI = 0.67–0.88) and there was high heterogeneity between studies (I^2^ 91.2%). There was insufficient data to calculate pooled kappa, PPV and NPV estimates for ASI-A.

##### Addiction severity index-drugs (drugs sub-scale; ASI-D)

The pooled alpha estimate for ASI-D (16 data points) was unsatisfactory: 0.68 (95%CI = 0.63–0.74) and there was high heterogeneity between studies (I^2^ 95.6%). The pooled sensitivity estimate (5 data points) was good: 0.86 (95%CI = 0.83–0.89) and there was moderate heterogeneity between studies (I^2^ 62.5%). The pooled specificity estimate (5 data points) was good: 0.85 (95%CI = 0.77–0.91) and there was high heterogeneity between studies (I^2^ 86%). There was insufficient data to calculate the pooled kappa, PPV and NPV estimates.

##### The alcohol, smoking, and substance involvement screening test (ASSIST)

The pooled alpha estimate (7 data points) was good: 0.85 (95%CI = 0.80–0.91) and there was high heterogeneity between studies (I^2^ 94%). The pooled sensitivity estimate (2 data points) was good: 0.83 (95%CI = 0.80–0.87) and there was low heterogeneity between studies (I^2^ 0%). The pooled specificity estimate (2 data points) was moderate: 0.73 (95%CI = 0.57–0.88) and there was high heterogeneity between studies (I^2^ 91%). There was insufficient data to calculate the pooled estimate for kappa, PPV, and NPV.

##### Alcohol use disorders identification test (AUDIT)

The pooled alpha estimate for AUDIT (80 data points) was moderate: 0.85 (95%CI = 0.83–0.87) and there was high heterogeneity between studies (I^2^ 98%). The pooled kappa estimate for AUDIT (4 data points) was unsatisfactory: 0.46 (95%CI = 0.25–0.67) and there was high heterogeneity between studies (I^2^ 0.99). The pooled sensitivity estimate for AUDIT (135 data points) was good: 0.86 (95%CI = 0.84–0.88) and there was high heterogeneity between studies (I^2^ 97%). The pooled specificity estimate for AUDIT (135 data points) was good: 0.87 (95%CI = 0.85–0.89) and there was high heterogeneity between studies (I^2^ 99%). The pooled PPV estimate for AUDIT (65 data points) was moderate: 0.61 (95%CI = 0.51–0.71) and there was high heterogeneity between studies (I^2^ 99%). The pooled NPV estimate for AUDIT (54 data points) was excellent: 0.94 (95%CI = 0.93–0.95) and there was high heterogeneity between studies (I^2^ 96%).

##### Alcohol use disorders identification Test-3 (AUDIT-3)

Alpha cannot be calculated for AUDIT-3 because it is a single-item measure. There was insufficient data to calculate the pooled estimate for kappa. The pooled sensitivity estimate for AUDIT-3 (22 data points) was good: 0.84 (95%CI = 0.80–0.88) and there was high heterogeneity between studies (I^2^ 90%). The pooled specificity estimate for AUDIT-3 (22 data points) was good: 0.84 (95%CI = 0.75–0.90) and there was high heterogeneity between studies (I^2^ 99%). The pooled PPV estimate for AUDIT-3 (9 data points) was moderate: 0.63 (95%CI = 0.49–0.77) and there was high heterogeneity between studies (I^2^ 99%). The pooled NPV estimate (7 data points) was excellent: 0.94 (95%CI = 0.90–0.98) and there was high heterogeneity between studies (I^2^ 95%).

##### Alcohol use disorders identification test-C (AUDIT-C)

The pooled alpha estimate for AUDIT-C (20 data points) was fair: 0.75 (95%CI = 0.70–0.80) and there was high heterogeneity between studies (I^2^ 99%). The pooled kappa estimate for AUDIT-C (2 data points) was unsatisfactory: 0.41 (95%CI = 0.39–0.43) and there was low heterogeneity between studies (I^2^ 0%). The pooled sensitivity estimate for AUDIT-C (45 data points) was good: 0.87 (95%CI = 0.84–0.90) and there was high heterogeneity between studies (I^2^ 99%). The pooled specificity estimate for AUDIT-C (45 data points) was good: 0.84 (95%CI = 0.81–0.87) and there was high heterogeneity between studies (I^2^ 99%). The pooled PPV estimate for AUDIT-C (22 data points) was low: 0.50 (95%CI = 0.39–0.60) and there was high heterogeneity between studies (I^2^ 99%). The pooled NPV estimate for AUDIT-C (19 data points) was good: 0.88 (95%CI = 0.83–0.92) and there was high heterogeneity between studies (I^2^ 99%).

##### Brief Michigan alcoholism screening test (B-MAST)

There was insufficient data to calculate the pooled estimate for B-MAST’s alpha and kappa. The pooled sensitivity estimate for B-MAST (21 data points) was low: 0.50 (95%CI = 0.38–0.62) and there was high heterogeneity between studies (I^2^ 99%). The pooled specificity estimate for B-MAST (21 data points) was excellent: 0.97 (95%CI = 0.96–0.98) and there was high heterogeneity between studies (I^2^ 97%). The pooled PPV estimate for B-MAST (3 data points) was moderate: 0.65 (95%CI = 0.38–0.93) and there was high heterogeneity between studies (I^2^ 99%). The pooled NPV estimate for B-MAST (2 data points) was excellent: 0.90 (95%CI = 0.87–0.94) and there was moderate heterogeneity between studies (I^2^ 33%).

##### Cut down, annoyed, guilty, eye-opener (CAGE)

The pooled alpha estimate for CAGE (22 data points) was unsatisfactory: 0.70 (95%CI = 0.65–0.75) and there was high heterogeneity between studies (I^2^ 98%). The pooled kappa estimate for CAGE (3 data points) was unsatisfactory: 0.57 (95%CI = 0.34–0.81) and there was high heterogeneity between studies (I^2^ 0.97). The pooled sensitivity estimate for CAGE (139 data points) was moderate: 0.70 (95%CI = 0.66–0.74) and there was high heterogeneity between studies (I^2^ 98%). The pooled specificity estimate for CAGE (139 data points) was good: 0.90 (95%CI = 0.88–0.91) and there was high heterogeneity between studies (I^2^ 99%). The pooled PPV estimate for CAGE (61 data points) was low: 0.51 (95%CI = 0.45–0.58) and there was high heterogeneity between studies (I^2^ 99%). The pooled NPV estimate for CAGE (39 data points) was excellent: 0.91 (95%CI = 0.88–0.93) and there was high heterogeneity between studies (I^2^ 97%).

##### Composite international diagnostic interview (CIDI), original version, version 2.1 and version 3

Alpha coefficients are not calculated for CIDI. The pooled kappa estimate for the original version of CIDI (2 data points) was moderate: 0.82 (95%CI = 0.61–1.02) and there was high heterogeneity between studies (I^2^ 0.78). There was insufficient data to calculate the pooled estimate for sensitivity, specificity, PPV, and NPV for the original CIDI.

The pooled sensitivity estimate for CIDI version 2.1 (3 data points) was fair: 0.75 (95%CI = 0.69–0.81) and there was low heterogeneity between studies (I^2^ 0.0%). The pooled specificity estimate for CIDI version 2.1 (3 data points) was good: 0.84 (95%CI = 0.69–1.00) and there was high heterogeneity between studies (I^2^ 98.7%). There was insufficient data to calculate the pooled estimate for kappa, PPV, and NPV for CIDI version 2.1.

The pooled sensitivity estimate for CIDI version 3 (4 data points) was excellent: 0.91 (95%CI = 0.82–1.00) and there was moderate heterogeneity between studies (I^2^ 48.1%). The pooled specificity estimate for CIDI version 3 (4 data points) was excellent: 0.99 (95%CI = 0.98–1.00) and there was low heterogeneity between studies (I^2^ 0.0%). The pooled PPV estimate for CIDI version 3 (4 data points) was excellent: 0.91 (95%CI = 0.87–0.96) and there was low heterogeneity between studies (I^2^ 0.0%). The pooled NPV estimate for CIDI version 3 (4 data points) was excellent: 0.99 (95%CI = 0.98–1.00) and there was low heterogeneity between studies (I^2^ 0.0%). There was insufficient data to calculate the pooled estimate for kappa CIDI version 3.

##### Car, relax, alone, forget, friends, trouble (CRAFFT)

The pooled alpha estimate for CRAFFT (6 data points) was unsatisfactory: 0.69 (95%CI = 0.64–0.74) and there was high heterogeneity between studies (I^2^ 83%). There was insufficient data to calculate the pooled estimate for kappa for CRAFFT. The pooled sensitivity estimate for CRAFFT (10 data points) was good: 0.90 (95%CI = 0.84–0.94) and there was high heterogeneity between studies (I^2^ 97%). The pooled specificity estimate for CRAFFT (10 data points) was moderate: 0.76 (95%CI = 0.68–0.83) and there was high heterogeneity between studies (I^2^ 97%). The pooled PPV estimate for CRAFFT (8 data points) was low: 0.57 (95%CI = 0.34–0.80) and there was high heterogeneity between studies (I^2^ 99%). The pooled NPV estimate for CRAFFT (8 data points) was good: 0.86 (95%CI = 0.45–1.00) and there was high heterogeneity between studies (I^2^ 99%).

##### Drug Abuse screen test (DAST)

The pooled alpha estimate for DAST (6 data points) was excellent: 0.94 (95%CI = 0.93–0.95) and there was low heterogeneity between studies (I^2^ 0%). The pooled kappa estimate for DAST (2 data points) was moderate: 0.83 (95%CI = 0.58–1.00) and there was high heterogeneity between studies (I^2^ 0.98). The pooled sensitivity estimate for DAST (7 data points) was good: 0.85 (95%CI = 0.74–0.92) and there was high heterogeneity between studies (I^2^ 89%). The pooled specificity estimate for DAST (7 data points) was good: 0.84 (95%CI = 0.68–0.93) and there was high heterogeneity between studies (I^2^ 97%). The pooled PPV estimate for DAST (5 data points) was low: 0.51 (95%CI = 0.32–0.70) and there was high heterogeneity between studies (I^2^ 98%). The pooled NPV estimate for DAST (4 data points) was excellent: 0.95 (95%CI = 0.89–1.00) and there was high heterogeneity between studies (I^2^ 81%).

##### Drug Abuse screen test - 10-item version (DAST-10)

The pooled alpha estimate DAST-10 (6 data points) was fair: 0.79 (95%CI = 0.68–0.89) and there was high heterogeneity between studies (I^2^ 98%). There was insufficient data to calculate the pooled estimate for kappa for DAST-10. The pooled sensitivity estimate for DAST-10 (6 data points) was excellent: 0.90 (95%CI = 0.75–0.97) and there was high heterogeneity between studies (I^2^ 95%). The pooled specificity estimate for DAST-10 (6 data points) was good: 0.82 (95%CI = 0.72–0.89) and there was high heterogeneity between studies (I^2^ 92%). The pooled PPV estimate for DAST-10 (4 data points) was good: 0.80 (95%CI = 0.70–0.91) and there was high heterogeneity between studies (I^2^ 99%). The pooled NPV estimate for DAST-10 (4 data points) was good: 0.86 (95%CI = 0.81–0.91) and there was moderate heterogeneity between studies (I^2^ 40%).

##### Drug use disorders identification test (DUDIT)

The pooled alpha estimate for DUDIT (15 data points) was excellent: 0.92 (95%CI = 0.90–0.95) and there was high heterogeneity between studies (I^2^ 96%). There was insufficient data to calculate the pooled kappa estimate for DUDIT. The pooled sensitivity estimate for DUDIT (12 data points) was excellent: 0.93 (95%CI = 0.89–0.96) and there was high heterogeneity between studies (I^2^ 76%). The pooled specificity estimate for DUDIT (12 data points) was moderate: 0.79 (95%CI = 0.67–0.87) and there was high heterogeneity between studies (I^2^ 96%). The pooled PPV estimate (5 data points) was moderate: 0.61 (95%CI = 0.34–0.87) and there was high heterogeneity between studies (I^2^ 99%). The pooled NPV estimate (5 data points) was excellent: 0.92 (95%CI = 0.82–1.00) and there was high heterogeneity between studies (I^2^ 78%).

##### Michigan alcohol screening test (MAST)

The pooled alpha estimate for MAST (8 data points) was moderate: 0.82 (95%CI = 0.78–0.86) and there was high heterogeneity between studies (I^2^ 83%). The pooled kappa estimate for MAST (4 data points) was unsatisfactory: 0.69 (95%CI = 0.58–0.81) and there was high heterogeneity between studies (I^2^ 0.88). The pooled sensitivity estimate for MAST (12 data points) was moderate: 0.70 (95%CI = 0.58–0.80) and there was high heterogeneity between studies (I^2^ 95%). The pooled specificity estimate for MAST (12 data points) was good: 0.85 (95%CI = 0.77–0.91) and there was high heterogeneity between studies (I^2^ 97%). The pooled PPV estimate for MAST (9 data points) was low: 0.51 (95%CI = 0.30–0.71) and there was high heterogeneity between studies (I^2^ 98%). The pooled NPV estimate for MAST (6 data points) was good: 0.88 (95%CI = 0.82–0.94) and there was high heterogeneity between studies (I^2^ 92%).

##### Problem oriented screening instrument for teenagers (POSIT)

The pooled alpha estimate for POSIT (2 data points) was good: 0.86 (95%CI = 0.73–0.98) and there was high heterogeneity between studies (I^2^ 94%). The pooled sensitivity estimate for POSIT (3 data points) was good: 0.84 (95%CI = 0.72–0.96) and there was high heterogeneity between studies (I^2^ 90%). The pooled specificity estimate for POSIT (3 data points) was good: 0.82 (95%CI = 0.75–0.90) and there was high heterogeneity between studies (I^2^ 88%). There was insufficient data to calculate the pooled kappa, PPV, and NPV estimates for POSIT.

##### Self-administered alcoholism screening test (SAAST)

The pooled alpha estimate for SAAST (2 data points) was good: 0.89 (95%CI = 0.79–0.99) and there was high heterogeneity between studies (I^2^ 95%). The pooled sensitivity estimate for SAAST (7 data points) was low: 0.52 (95%CI = 0.33–0.71) and there was high heterogeneity between studies (I^2^ 98%). The pooled specificity estimate (7 data points) was good: 0.83 (95%CI = 0.76–0.90) and there was high heterogeneity between studies (I^2^ 98%). The pooled PPV estimate for SAAST (6 data points) was low: 0.32 (95%CI = 0.22–0.42) and there was high heterogeneity between studies (I^2^ 95%). The pooled NPV estimate for SAAST (6 data points) was excellent: 0.92 (95%CI = 0.89–0.95) and there was high heterogeneity between studies (I^2^ 92%). There was insufficient data to calculate the pooled kappa estimates for SAAST.

##### Semi-structured assessment for drug dependence and alcoholism (SSADDA)

There are no alpha coefficients associated with semi-structures assessments such as SSADDA. The pooled kappa estimate for SSADDA (8 data points) was moderate: 0.84 (95%CI = 0.77–0.91) and there was high heterogeneity between studies (I^2^ 0.97). There was insufficient data to calculate the pooled sensitivity, specificity, PPV and NPV estimates for SSADDA.

##### Severity of dependence (SDS)

The pooled alpha estimate for SDS (6 data points) was good: 0.86 (95%CI = 0.78–0.93) and there was high heterogeneity between studies (I^2^ 95%). The pooled sensitivity estimate for SDS (6 data points) was good: 0.83 (95%CI = 0.76–0.90) and there was high heterogeneity between studies (I^2^ 77%). The pooled specificity estimate (6 data points) was good: 0.84 (95%CI = 0.78–0.89) and there was moderate heterogeneity between studies (I^2^ 44%). The pooled PPV estimate for SDS (3 data points) was good: 0.90 (95%CI = 0.86–0.94) and there was low heterogeneity between studies (I^2^ 0%). The pooled NPV estimate for SDS (3 data points) was good: 0.83 (95%CI = 0.76–0.89) and there was low heterogeneity between studies (I^2^ 3.5%). There was insufficient data to calculate the pooled kappa estimate for SDS.

##### Tolerance-annoyance cut down eye opener (T-ACE)

The pooled alpha estimate for T-ACE (2 data points) was unsatisfactory: 0.50 (95%CI = 0.47–0.52) and there was high heterogeneity between studies (I^2^ 29%). The pooled sensitivity estimate for T-ACE (8 data points) was good: 0.83 (95%CI = 0.74–0.92) and there was high heterogeneity between studies (I^2^ 96%). The pooled specificity estimate for T-ACE (8 data points) was moderate: 0.72 (95%CI = 0.65–0.79) and there was high heterogeneity between studies (I^2^ 98%). The pooled PPV estimate for T-ACE (6 data points) was low: 0.35 (95%CI = 0.25–0.45) and there was high heterogeneity between studies (I^2^ 99%). The pooled NPV estimate for T-ACE (2 data points) was good: 0.87 (95%CI = 0.62–1.00) and there was high heterogeneity between studies (I^2^ 97%). There was insufficient data to calculate the pooled estimate for kappa for T-ACE.

##### Timeline Followback (TLFB)

There are no alpha coefficients associated with TLFB. The pooled kappa estimate for TLFB (3 data points) was good: 0.86 (95%CI = 0.81–0.91) and there was high heterogeneity between studies (I^2^ 0.88). The pooled sensitivity estimate for TLFB (4 data points) was moderate: 0.80 (95%CI = 0.73–0.87) and there was moderate heterogeneity between studies (I^2^ 63%). The pooled specificity estimate for TLFB (3 data points) was excellent: 0.97 (95%CI = 0.95–0.99) and there was low heterogeneity between studies (I^2^ 0%). There was insufficient data to calculate the pooled estimate for PPV and NPV for TLFB.

##### Tolerance, worried, eye-opener, amnesia, cut down (TWEAK)

The pooled alpha estimate for TWEAK (3 data points) was unsatisfactory: 0.62 (95%CI = 0.55–0.69) and there was high heterogeneity between studies (I^2^ 86%). The pooled sensitivity estimate for TWEAK (36 data points) was good: 0.85 (95%CI = 0.80–0.89) and there was high heterogeneity between studies (I^2^ 96%). The pooled specificity estimate for TWEAK (36 data points) was good: 0.86 (95%CI = 0.82–0.90) and there was high heterogeneity between studies (I^2^ 99%). The pooled PPV estimate for TWEAK (5 data points) was low: 0.43 (95%CI = 0.26–0.61) and there was high heterogeneity between studies (I^2^ 99%). The pooled NPV estimate for TWEAK (2 data points) was good: 0.88 (95%CI = 0.70–1.00) and there was high heterogeneity between studies (I^2^ 95%). There was insufficient data to calculate the pooled estimate for kappa for TWEAK.

##### The chemical use, Abuse, and dependence (CUAD)

The pooled alpha estimate for CUAD (3 data points) was excellent: 0.96 (95%CI = 0.94–0.98) and there was high heterogeneity between studies (I^2^ 95%). There was insufficient data to calculate the pooled estimate for kappa, sensitivity, specificity, PPV, and NPV for CUAD.

### Biomarkers

#### Alanine transaminase (ALT)

The pooled sensitivity estimate for ALT (32 data points) was low: 0.32 (95%CI = 0.24–0.40) and there was high heterogeneity between studies (I^2^ 96.1%). The pooled specificity estimate for ALT (32 data points) was good: 0.88 (95%CI = 0.83–0.92) and there was high heterogeneity between studies (I^2^ 95.8%). The pooled PPV estimate for ALT (7 data points) was low 0.37 (95%CI = 0.18–0.56) and there was high heterogeneity between studies (I^2^ 96.1%). The pooled NPV estimate for ALT (4 data points) was moderate: 0.63 (95%CI = 0.42–0.85) and there was high heterogeneity between studies (I^2^ 97.5%).

#### Aspartate transaminase (AST)

The pooled sensitivity estimate for AST (33 data points) was low: 0.48 (95%CI = 0.40–0.55) and there was high heterogeneity between studies (I^2^ 97%). The pooled specificity estimate for AST (33 data points) was good: 0.86 (95%CI = 0.81–0.90) and there was high heterogeneity between studies (I^2^ 97%). The pooled PPV estimate for AST (8 data points) was low: 0.42 (95%CI = 0.27–0.57) and there was high heterogeneity between studies (I^2^ 93%). The pooled NPV estimate for AST (6 data points) was moderate: 0.69 (95%CI = 0.55–0.83) and there was high heterogeneity between studies (I^2^ 95%).

#### Aspartate transaminase, alanine transaminase ratio (AST/ALT ratio)

The pooled sensitivity estimate for AST/ALT ratio (6 data points) was low: 0.34 (95%CI = 0.22–0.46) and there was high heterogeneity between studies (I^2^ 96%). The pooled specificity estimate (4 data points) was moderate: 0.73 (95%CI = 0.52–0.94) and there was high heterogeneity between studies (I^2^ 98%). There was insufficient data to calculate the pooled estimate for PPV and NPV.

#### Blood alcohol concentration (BAC)

The pooled sensitivity estimate for BAC (5 data points) was moderate: 0.64 (95%CI = 0.59–0.69) and there was moderate heterogeneity between studies (I^2^ 44%). The pooled specificity estimate for BAC (5 data points) was moderate: 0.80 (95%CI = 0.72–0.87) and there was high heterogeneity between studies (I^2^ 93%). The pooled PPV estimate for BAC (3 data points) was low: 0.60 (95%CI = 0.15–1.00) and there was high heterogeneity between studies (I^2^ 98%). The pooled NPV estimate for BAC (3 data points) was moderate: 0.69 (95%CI = 0.52–0.86) and there was high heterogeneity between studies (I^2^ 93%).

#### Carbohydrate deficient transferrin (CDT)

There are no alpha and kappa coefficients associated with biomarkers such as CDT. The pooled sensitivity estimate for CDT (8 data points) was low: 0.59 (95%CI = 0.43–0.73) and there was high heterogeneity between studies (I^2^ 97%). The pooled specificity estimate for CDT (8 data points) was excellent: 0.96 (95%CI = 0.93–0.98) and there was moderate heterogeneity between studies (I^2^ 72%). The pooled PPV estimate for CDT (6 data points) was good: 0.85 (95%CI = 0.74–0.97) and there was high heterogeneity between studies (I^2^ 76%). The pooled NPV estimate for CDT (6 data points) was moderate: 0.79 (95%CI = 0.73–0.85) and there was high heterogeneity between studies (I^2^ 96%).

#### Carbohydrate deficient transferrin-tech (CDTech)

There are no alpha and kappa coefficients associated with biomarkers such as CDTech. The pooled sensitivity estimate for CDTech (41 data points) was low: 0.54 (95%CI = 0.45–0.62) and there was high heterogeneity between studies (I^2^ 99%). The pooled specificity estimate for CDTech (41 data points) was good: 0.89 (95%CI = 0.88–0.91) and there was high heterogeneity between studies (I^2^ 88%). The pooled PPV estimate for CDTech (12 data points) was low: 0.52 (95%CI = 0.37–0.67) and there was high heterogeneity between studies (I^2^ 95%). The pooled NPV estimate for CDTech (8 data points) was moderate: 0.80 (95%CI = 0.61–0.98) and there was high heterogeneity between studies (I^2^ 99%).

#### Carbohydrate deficient transferrin with mean corpuscular volume (CDT with MCV)

There are no alpha and kappa coefficients associated with biomarkers such as CDT and MCV. The pooled sensitivity estimate for CDT with MCV (8 data points) was moderate: 0.74 (95%CI = 0.60–0.88) and there was high heterogeneity between studies (I^2^ 98%). The pooled specificity estimate for CDT with MCV (4 data points) was excellent: 0.93 (95%CI = 0.91–0.95) and there was low heterogeneity between studies (I^2^ 0%). The pooled PPV estimate for CDT with MCV (4 data points) was moderate: 0.74 (95%CI = 0.51–0.97) and there was high heterogeneity between studies (I^2^ 98%). The pooled NPV estimate for CDT with MCV (4 data points) was excellent: 0.92 (95%CI = 0.83–1.00) and there was high heterogeneity between studies (I^2^ 95%).

#### Gamma-Glutamyl Transferase (GGT)

There are no alpha and kappa coefficients associated with biomarkers such as GGT. The pooled sensitivity estimate for GGT (76 data points) was low: 0.57 (95%CI = 0.50–0.64) and there was high heterogeneity between studies (I^2^ 99%). The pooled specificity estimate for GGT (76 data points) was good: 0.83 (95%CI = 0.78–0.86) and there was high heterogeneity between studies (I^2^ 98%). The pooled PPV estimate for GGT (30 data points) was low: 0.43 (95%CI = 0.35–0.51) and there was high heterogeneity between studies (I^2^ 97%). The pooled NPV estimate for GGT (23 data points) was good: 0.82 (95%CI = 0.70–0.94) and there was high heterogeneity between studies (I^2^ 99%).

#### Gamma-Glutamyl Transferase with mean corpuscular volume (GGT with MCV)

There are no alpha and kappa coefficients associated with biomarkers such as GGT and MCV. The pooled sensitivity estimate for GGT with MCV (10 data points) was moderate: 0.64 (95%CI = 0.38–0.84) and there was high heterogeneity between studies (I^2^ 99%). The pooled specificity estimate for GGT with MCV (10 data points) was good: 0.87 (95%CI = 0.76–0.93) and there was high heterogeneity between studies (I^2^ 97%). The pooled PPV estimate for GGT with MCV (6 data points) was low: 0.47 (95%CI = 0.28–0.66) and there was high heterogeneity between studies (I^2^ 98%). The pooled NPV estimate for GGT with MCV (6 data points) was good: 0.88 (95%CI = 0.81–0.95) and there was high heterogeneity between studies (I^2^ 94%).

#### Ethyl glucuronide (EtG)

There are no alpha and kappa coefficients associated with biomarkers such as EtG. The pooled sensitivity estimate for EtG (6 data points) was good: 0.83 (95%CI = 0.61–0.94) and there was high heterogeneity between studies (I^2^ 91%). The pooled specificity estimate for EtG (6 data points) was excellent: 0.95 (95%CI = 0.90–0.98) and there was high heterogeneity between studies (I^2^ 66%). The pooled PPV estimate for EtG (2 data points) was moderate: 0.61 (95%CI = 0.39–0.84) and there was moderate heterogeneity between studies (I^2^ 58%). The pooled NPV estimate for EtG (2 data points) was good: 0.86 (95%CI = 0.78–0.94) and there was moderate heterogeneity between studies (I^2^ 60%).

#### Mean corpuscular volume (MCV)

There are no alpha and kappa coefficients associated with biomarkers such as MCV. The pooled sensitivity estimate for MCV (55 data points) was low: 0.39 (95%CI = 0.33–0.45) and there was high heterogeneity between studies (I^2^ 97%). The pooled specificity estimate for MCV (55 data points) was excellent: 0.91 (95%CI = 0.88–0.93) and there was high heterogeneity between studies (I^2^ 98%). The pooled PPV estimate for MCV (28 data points) was low: 0.48 (95%CI = 0.36–0.59) and there was high heterogeneity between studies (I^2^ 98%). The pooled NPV estimate for MCV (22 data points) was moderate: 0.79 (95%CI = 0.73–0.86) and there was high heterogeneity between studies (I^2^ 99%).

#### Percent carbohydrate deficient transferrin (%CDT)

The pooled sensitivity estimate for %CDT (40 data points) was low: 0.56 (95%CI = 0.47–0.65) and there was high heterogeneity between studies (I^2^ 98.2%). The pooled specificity estimate for %CDT (40 data points) was 0.91, which is considered as excellent (95%CI = 0.88–0.94) and there was high heterogeneity between studies (I^2^ 97%). The pooled PPV estimate for %CDT (13 data points) was low: 0.58 (95%CI = 0.38–0.78) and there was high heterogeneity between studies (I^2^ 98.5%). The pooled NPV estimate for %CDT (13 data points) was good: 0.85 (95%CI = 0.78–0.92) and there was high heterogeneity between studies (I^2^ 97.6%).

#### Phosphatidylethanol (PEth)

There are no alpha and kappa coefficients associated with biomarkers such as PEth. The pooled sensitivity estimate for PEth (7 data points) was good: 0.87 (95%CI = 0.79–0.96) and there was high heterogeneity between studies (I^2^ 94%). The pooled specificity estimate for PEth (4 data points) was excellent: 0.94 (95%CI = 0.91–0.97) and there was moderate heterogeneity between studies (I^2^ 31%). There was insufficient data to calculate the pooled estimate for PPV and NPV for PEth.

## Discussion

In this systematic review and meta-analysis, we identified 387 unique papers that have published data on the validity, reliability and diagnostic accuracy of 37 scales for substance classes that are associated with HIV risk. We observed based on meta-analyzable data available, that fourteen of the thirty-seven measures/scales (38%) that had all pooled estimates consistently meet criteria for acceptability (e.g., ranging between fair/moderate-to-excellent), which included the following self-reported measures:

- Alcohol Dependence Scale
- Addiction Severity Index (ASI)
- ASI subscale for Alcohol; ASSIST
- Composite International Diagnostic Interview (version original, version 2.1, and version 3)
- Drug Abuse Screen Test - 10 item scale
- Drug Use Disorders Identification Test
- Problem Oriented Screening Instrument for Teenagers
- Severity of Dependence scale
- Timeline Followback
- Chemical Use, Abuse, and Dependence

Biomarkers that had all pooled estimates that were fair/moderate or better include the following:

- Ethyl glucuronide
- Phosphatidylethanol test
- The combined used of Carbohydrate deficient transferrin and Mean corpuscular volume.

Taken together, our findings highlight the availability of a promising range of tools for researchers and practitioners when assessing substance use, particularly those working with classes of substances associated with HIV risk, such as heroin, methamphetamine, cocaine, ecstasy, and alcohol. Nevertheless, further research is needed to determine why some substance use measures do not consistently have acceptable psychometric properties across different studies.

Overall, while most of the self-reported scales had acceptable validity, most did not have acceptable reliability: 65% of pooled estimates for alpha were in the range of fair-to-excellent though only 44% of estimates for kappa were in the range of fair-to-excellent. Moreover, a greater proportion of the scales we identified and meta-analyzed were better at correctly identifying individuals who are truly not using substances/not problematic users among those truly without these conditions (specificity: 97% of summary estimates) and among those who were deemed as not having this condition in the scale (negative predictive value: 96%). In contrast to specificity and negative predictive value estimates, fewer scales had pooled estimates on sensitivity and positive predictive value that were in the fair-to-excellent range (69 and 37%, respectively). These may have implications in the application of these measures in different settings. For example, in the criminal justice system, it may be better to utilize measures that have high specificity and negative predictive properties if the priority is to avoid false-positive results. However, in health settings, it may be more ideal to use measures with better sensitivity and positivity to better capture individuals who may require further assessment for substance use disorder assessments and treatment referrals, as appropriate.

Overall, the studies identified in this review had administered scales in English, were conducted within in the United States, and were less commonly tested among exclusively-women samples (there were twice as many exclusively-men samples in comparison). These findings highlight the general lack of diversity in terms of language, setting, and study population for the studies reporting validity, reliability, and diagnostic accuracy on substance use measures. Given the high morbidity and mortality associated with substance use globally and for different risk populations, greater effort is needed to further evaluate the psychometric properties of substance use measures in such samples. This study also found that few papers on substance use psychometric properties are “low risk” across all QUADAS 2 domains (16%). This finding highlights the need to further study the validity, reliability, and diagnostic accuracy of substance use measures using studies designed with better methodological rigor to reduce risk of bias.

This present study has several limitations. First, our inclusion criteria may have excluded some potentially relevant studies on the psychometric properties of substance use measures that were not published in English. Hence, although we included measures that were not administered in English as long as they were published in English, our findings may not necessarily be generalizable to the psychometric properties of non-English measures that were not published in English. It should also be noted that our eligibility criteria likely favored the inclusion of studies that were conducted in settings where English proficiency was higher, which is correlated with countries with higher gross national income per capita [43]. Moreover, while our search strategy was developed to try and identify all the relevant studies, many publications that have calculated our psychometric properties of interest may not have language referencing the specific key words/terms in our strategy in their titles and/or abstracts. In particular, this may occur because the psychometric data of scales may not be considered a “primary outcome” of a study, and thus not be highlighted in the title or abstract (i.e., the relevant data are imbedded within the full-text only). Additionally, while we did not specifically seek out studies only among HIV-risk populations, per se, our study did focus on substance classes that have been associated with HIV risk, namely alcohol, stimulants (methamphetamine, amphetamine, cocaine, ecstasy), and heroin. Hence, our search may have missed studies on more general substance use measures that did not explicitly name our targeted substance classes. Furthermore, we were unable to calculate pooled estimates for some psychometric outcomes of several measures due to lack of published data or insufficient data, including for some widely used assessments previously shown to be valid and reliable, such as the DSM-IV diagnostic modules used in the US National Surveys of Drug Use and Health, the Diagnostic Interview schedule, and the AUDADIS [44–46]. Another limitation in our meta-analysis is related to our narrow definition of validity, which focused on internal validity as measured by Cronbach’s alpha values. We acknowledge that there are a range of other characteristics that examine validity that we did not include in our analysis such as criterion validity, predictive validity, and other psychometric properties [32]. Further research is needed to fill our gaps in knowledge on the psychometric properties of these substance use measures to enable pooled summary estimate calculations. In addition, we recognize the limitation from pooling alpha and kappa statistics from clinical and epidemiologic/community samples given how these statistical measures are margin-sensitive. Moreover, with respect to the synthesis of data on sensitivity and specificity, we acknowledge that some studies may have used imperfect gold-standards, which may lead to distorted values for the individual estimates for sensitivity and specificity. Therefore, it may be appropriate to refer to results as co-positivity and co-negativity, as suggested by Buck and Gart [47]. Finally, we also recognized that disease spectrum severity and prevalence can affect test performance for sensitivity and specificity [48, 49]. Our results should be interpreted with these limitations in mind.

To our knowledge, this is the first systematic review and meta-analysis involving the synthesis of psychometric data across different measures of substances that are associated with HIV risk. As mentioned, limited research has been conducted with respect with quantitatively pooling the psychometric characteristics of substance use measures. Our findings highlight the general strengths of many substance use measures with respect to their validity, reliability, and diagnostic accuracy across multiple studies/samples. To facilitate the dissemination of these findings, and provide researchers with a resource to identify validated, reliable, and accurate measures for substance use, we collaborated with members of the HIV Prevention Trials Network (HPTN) Substance Use Scientific Committee to develop a web-based tool, with the results of the pooled summary estimates presented in this study. The tool, named “Substance Use Measure Identification (SUMI) Tool” is available as a free resource in the HPTN's website (URL: https://www.hptn.org/researchtools).

## Conclusion

In summary, researchers in the field of substance use should endeavor to conduct more validity, reliability, and diagnostic accuracy studies on measures to identify substance use and use disorders among more diverse settings and populations, and with more rigorous study designs. Ultimately, accurate identification of substance users and problematic substance use is a critical step in identifying individuals for substance use treatment and evaluating the effectiveness of treatment strategies. Hence, further evaluation of substance use measures is of great importance not only to the field of substance use research, but also substance use treatment. Given the substantial contribution of substance use to the global burden of disease [5], having robust data on the.

psychometric properties of substance use measure can help researchers identify the best tools to use in research studies, further enhancing the collection of more valid, reliable, accurate data to inform evidence-based responses to substance use.

## Supplementary information

**Additional file 1.**

**Additional file 2: Table S1.** Characteristics and Risk of Bias Studies Included in Meta-Analyses. **Table S2.** References of Studies Meta-Analyzed, by Scale.

## Data Availability

All data used in this meta-analyses have been previously published and accessible in the literature.

## Abbreviations

%CDT: % Carbohydrate deficient transferrin
ADS: Alcohol Dependence Scale
ALT: Alanine transaminase
ART: Antiretroviral therapy
ASI: Addiction Severity Index
ASI-A: Addiction Severity Index-Alcohol (alcohol sub-scale)
ASI-D: Addiction Severity Index-Drugs (drugs sub-scale)
ASSIST: The Alcohol, Smoking, and Substance Involvement Screening Test
AST: Aspartate transaminase
AST/ALT: Aspartate transaminase, Alanine transaminase ratio
AUDADIS: Alcohol Use Disorder and Associated Disabilities Interview Schedule
AUDIT: Alcohol Use Disorders Identification Test
AUDIT-3: Alcohol Use Disorders Identification Test - Question 3
AUDIT-C: Alcohol Use Disorders Identification Test - C
B-MAST: Brief Michigan Alcoholism Screening Test
BAC: Blood alcohol concentration
CAGE: Cut down, Annoyed, Guilty, Eye-opener
CDT: Carbohydrate deficient transferrin
CDTech: CDTech
CDT + MCV: Carbohydrate deficient transferrin + Mean corpuscular volume
CIDI: Composite International Diagnostic Interview
CRAFFT: Car, Relax, Alone, Forget, Friends, Trouble
CUAD: The Chemical Use, Abuse, and Dependence
DALY: Disability-adjusted life year
DAST: Drug Abuse Screen Test
DAST-10: Drug Abuse Screen Test – 10 item
DSM: Diagnostic and Statistical Manual of Mental Disorders
DUDIT: Drug Use Disorders Identification Test
GGT: Gamma-Glutamyl Transferase
GGT + MCV: Gamma-Glutamyl Transferase + Mean corpuscular volume
HIV: Human immunodeficiency virus
EtG: Ethyl glucuronide
MAST: Michigan Alcohol Screening Test
MCV: Mean corpuscular volume
MDMA: 3,4-methylenedioxy-methamphetamine
MeSH: Medical Subject Headings
MLIS: Master’s degree in library and information science
NPV: Negative predictive value
PEth: Phosphatidylethanol
PLWH: People living with HIV
POSIT: Problem Oriented Screening Instrument for Teenagers
PPV: Positive predictive value
QUADAS-2: Revised Tool for the Quality Assessment of Diagnostic Accuracy Studies
SAAST: Self-Administered Alcoholism Screening Test
SSADDA: Semi-Structured Assessment for Drug Dependence and Alcoholism
SDS: Severity of Dependence
TACE: Tolerance-Annoyance Cut Down Eye Opener
TLFB: Timeline Followback
TWEAK: Tolerance, Worried, Eye-Opener, Amnesia, Cut down
