# Supplementary material for: Psychometric properties of measures of substance use: a systematic review and meta-analysis of reliability, validity and diagnostic test accuracy
Source: BMC Med Res Methodol. 2020 May 7;20:106. doi: 10.1186/s12874-020-00963-7 (PMC7203822; doi:10.1186/s12874-020-00963-7)
Supplement: Supplementary file 1 — Additional file 1. [file 12874_2020_963_MOESM1_ESM.docx]

**((((((((“substance related disorders”[majr] OR “substance use” [tiab] OR “substance abuse” [tiab] OR “substance use disorders” [tiab] OR “substance addiction” [tiab] OR “drug use” [tiab] OR “drug abuse” [tiab] OR “drug use disorders” [tiab] OR “drug addiction” [tiab] OR “drug dependence” [tiab] OR alcoholism [tiab] OR “alcohol abuse” [tiab] OR “alcohol use disorder” [tiab]) AND (survey* [tiab] OR “surveys and questionnaires”[mh])) OR (DAST[tiab] AND (substance*[tiab] OR abus*[tiab])) OR “Drug Abuse Screening Test” [tiab] OR (DSM[tiab] AND Checklist*[tiab] and substance*[tiab]) OR (Form-90[tiab] AND (substance*[tiab] OR abus*[tiab])) OR (CIDI[tiab] AND (substance*[tiab] OR abus*[tiab])) OR "composite international diagnostic interview"[tiab] OR "Mini-International Neuropsychiatric Interview"[tiab] OR ("MINI PLUS"[tiab] AND (substance*[tiab] OR abus*[tiab])) OR “Risk Behavior Assessment” [tiab] OR “Alcohol Use Disorder Identification Test”[tiab] OR (“AUDIT” [tiab] AND alcohol*[tiab]) OR ("Addiction Severity Index" [tiab] OR (ASI[tiab] AND (substance[tiab] OR abuse[tiab]))) OR (CAGE[tiab] AND alcohol*[tiab]) OR (“Alcohol, Smoking and Substance Involvement Screening Test” [tiab] OR (ASSIST[tiab] AND (substance*[tiab] OR alcohol*[tiab]))) OR (“Time-Line Follow-Back” [tiab] OR (TLFB[tiab] AND (alcohol*[tiab]))) OR “Substance Use Calendar” [tiab] OR (“Diagnostic Interview Schedule for Children” [tiab] OR (DISC-P[tiab] OR DISC-Y [tiab] AND (substance* [tiab] OR abus*[tiab]))) OR “Global Appraisal of Individual Needs” [tiab] OR (GAIN[tiab] AND (substance*[tiab] OR abus*[tiab])) OR ((“Texas Christian University Drug Screen” [tiab] OR TCUDS[tiab]) AND (substance*[tiab] OR abus*[tiab])) OR (("Substance Abuse Detection"[Mesh])) OR (“rapid urine drug”[tiab] OR “carbohydrate deficient transferrin” OR gamma glutamyl transferase OR “Mean corpuscular volume” OR “Phosphatidylethanol” OR “Blood alcohol concentration” OR “Blood alcohol level” OR “alanine aminotransferase” OR “aspartate aminotransferase” OR “Ethyl glucuronide” OR “Ethyl sulfate” OR “Fatty acid ethyl esters” OR “Breath alcohol concentration” OR Breathalyzer OR “Breath test”)) OR ((("Sweat screen" OR "Sweat screens" OR "Sweat screening" OR "Sweat test" OR "Sweat tests" OR "Sweat patch" OR "Sweat patches" OR "Sweat toxicology" OR "Sweat analysis" OR "Sweat analyses" OR "Blood screen" OR "Blood screens" OR "Blood screening" OR "Blood test" OR "Blood tests" OR "Blood testing" OR Blood patch* OR "Blood toxicology" OR "Blood analysis" OR "Blood analyses" OR "Plasma screen" OR "Plasma screens" OR "Plasma screening" OR "Plasma test" OR "Plasma tests" OR "Plasma tests" OR "Plasma testing" OR "Plasma patch" OR "Plasma patches" OR "Plasma toxicology" OR "Plasma analysis" OR "Plasma analyses" OR "Serum screen" OR "Serum screens" OR "Serum screening" OR "Serum test" OR "Serum tests" OR "Serum testing" OR "Serum patch" OR "Serum patches" OR "Serum toxicology" OR "Serum analysis" OR "Serum analyses" OR "Urine screen" OR "Urine screens" OR "Urine screening" OR "Urine test" OR "Urine tests" OR "Urine testing" OR "Urine toxicology" OR "Urine analysis" OR "Urine analyses" OR Urinalysis OR Urinalyses OR "Oral fluid screen" OR "Oral fluid screens" OR "Oral fluid screening" OR "Oral fluid test" OR "Oral fluid tests" OR "Oral fluid testing" OR "Oral fluid toxicology" OR "Oral fluid analysis" OR "Oral fluid analyses" OR "Saliva screen" OR "Saliva screens" OR "Saliva screening" OR "Saliva test" OR "Saliva tests" OR "Saliva testing" OR "Saliva toxicology" OR "Saliva analysis" OR "Saliva analyses" OR "Hair screen" OR "Hair screens" OR "Hair screening" OR "Hair test" OR "Hair tests" OR "Hair testing" OR "Hair toxicology" OR "Hair analysis" OR "Hair analyses" OR "Nail screen" OR "Nail screens" OR "Nail screening" OR "Nail test" OR "Nail tests" OR "Nail testing"OR "Nail toxicology" OR "Nail analysis" OR "Nail analyses") AND (alcohol [tiab] OR ethanol [tiab] OR “drug use” [tiab] OR “substance use”[tiab]))))))))**

**AND**

**((“Psychometrics”[majr] OR psychometric* [tiab] OR “internal consistency” [tiab] OR “test re-test” [tiab] OR “Intra-class Correlation Coefficient” [tiab] OR “cronbach* alpha” [tiab] OR “cronbach* Î±” [tiab] OR (sensitivity[tiab] AND specificity[tiab]) OR "Sensitivity and Specificity"[Mesh] OR "Reproducibility of Results"[Mesh] OR (reliability[tiab] AND validity [tiab]) OR “positive predictive value”[tiab] OR “negative predictive value”[tiab] OR kappa[tiab] OR “kappa coefficient”[tiab])))))**
