# Supplementary material for: Psychometric properties of measures of substance use: a systematic review and meta-analysis of reliability, validity and diagnostic test accuracy
Source: BMC Med Res Methodol. 2020 May 7;20:106. doi: 10.1186/s12874-020-00963-7 (PMC7203822; doi:10.1186/s12874-020-00963-7)
Supplement: Supplementary file 2 — Additional file 2: Table S1. Characteristics and Risk of Bias Studies Included in Meta-Analyses. Table S2. References of Studies Meta-Analyzed, by Scale. [file 12874_2020_963_MOESM2_ESM.docx]

| **Supplementary** **Table 1. Characteristics and Risk of Bias Studies Included in Meta-Analyses** | | | | | | | | | | | |
| --- | --- | --- | --- | --- | --- | --- | --- | --- | --- | --- | --- |
|  |  |  |  |  |  |  | **QUADAS 2 Risk of Bias** | | | | **Age** |
| **First Author** | **Year** | **Sample Size** | **Country where study was conducted** | **Sites** | **Language Scale was administered** | **Gender of Participants Included** | **Patient Selection** | **Index Test** | **Referent Test^1^** | **Flow and Timing** | **Mean (SD)**  **Median(IQR or Range)** |
| Watson, C. G | 1995 | 118 | USA | Single site | English | Women, Men | unclear | low | low | low | Mean: 46 |
| Velez M | 2015 | 109 | Spain | Single site | Spanish | Women, Men | high | low | low | low | Mean: 39.8 |
| Velez-Moreno A | 2015 | 211 | Spain | Single site | Spanish | Women, Men | low | high |  | low | Mean: 39.2 |
| Vazquez F | 2007 | 554 | Spain | Single site | Spanish | Women, Men | low | unclear | unclear | low | Mean: 22.2 |
| Stamm D | 1984 | 152 | Germany | Multi-site | Other | Men | unclear | low | low | low | Not Reported |
| Schellenberg F | 2001 | 320 | France | Single site | French | Women, Men | unclear | low | low | unclear | Not Reported |
| Ramchand R | 2009 | 677 | USA | Multi-site | English, Spanish | Women, Men | high | high | high | low | Not Reported |
| Quintana M | 2007 | 185 | Brazil | Multi-site | Portuguese | Women, Men | low | unclear | low | low | Mean: 37 |
| Pedrelli P | 2013 | 332 | USA | Single site | English | Women, Men | low | low | low | low | Mean: 19 |
| McDermott P | 1996 | 990 | USA | Multi-site | English | Women, Men | low | unclear |  | low | Mean: 38.9 |
| Legros F | 2003 | 614 | Belgium | Multi-site | French | Women, Men | low | low | unclear | low | Mean: 51 |
| Hurme, L | 1998 | 87 | Finland | Multi-site | Other | Women | high | high | high | low | Mean: 42 |
| Gryczynski J | 2015 | 525 | USA | Single site | English | Women, Men | high | low | low | high | Not Reported |
| Denis C | 2012 | 1848 | USA | Multi-site | English | Women, Men | low | unclear | unclear | high | Mean: 32 |
| Cummins, L. | 2003 | 70 | France | Multi-site | French | Women, Men | low | low | low | low | Mean: 16 |
| Cherpitel C | 2005 | 1492 | USA | Multi-site | English | Women, Men | high | low | low | unclear | Not Reported |
| Cherpitel C | 1995 | 1330 | Poland | Multi-site | Other | Women, Men | low | low | high | high | Not Reported |
| Cherpitel C | 1999 | 2443 | USA | Single site | English | Women, Men | low | low | unclear | high | Not Reported |
| Cherpitel C | 1998 | 1429 | USA | Multi-site | English | Women, Men | low | low | unclear | unclear | Not Reported |
| Borges G | 2001 | 869 | USA | Single site | English | Women, Men | low | low |  | high | Not Reported |
| Alte D | 2004 | 4310 | Mexico, USA | Multi-site | English, Spanish | Women, Men | low | low | high | low | Mean: 50.3 |
| Bernard M | 2005 | 163 | Germany | Multi-site | Other | Women, Men | low | low | high | low | Mean: 18.1 |
| El-Bassel N | 1997 | 176 | Switzerland | Single site | French | Women, Men | unclear | high | unclear | unclear | Mean: 38.7 |
| Gerevich, J | 2005 | 266 | USA | Single site | English | Women, Men | high | unclear |  | low | Mean: 26.7 |
| Skogen J | 2013 | 9680 | Hungary | Single site | Other | Women, Men | high | unclear |  | unclear | Mean: 17.8 |
| Saunders J | 1993 | 1888 | Norway | Multi-site | Unclear | Women, Men | low | high | high | high | Not Reported |
| Watson, C. G | 1995 | 118 | Australia, Bulgaria, Kenya, Mexico, Norway, USA | Multi-site | English, Spanish, Other | Women, Men | unclear | unclear |  | low | Mean: 46 |
| Aalto A | 2011 | 517 | Finland | Multi-site | Other | Women, Men | low | low | low | low | Mean: 69 (2.8) Range: 25-74 |
| Aalto M | 2006 | 894 | Finland | Multi-site | English | Women | low | low | low | low | Not Reported |
| Aalto M | 2009 | 1851 | Finland | Multi-site | Other | Women, Men | low | low | low | low | Range: 25-64 |
| Adewuya A | 2005 | 810 | Nigeria | Single site | English | Women, Men | low | low | low | low | Mean: 22.5 (5.2) |
| Aertgeert B | 2000 | 501 | Belgium | Single site | English, Other | Women, Men | low | low | low | low | Mean: 18 |
| Aertgreerts B. | 2001 | 1992 | United Kingdom | Single site | English | Women, Men | low | low | low | low | Male Mean: 54 Range: 18+  Female Mean: 48 Range: 18+ |
| Agabio R | 2007 | 56 | Italy | Single site | English, Other | Women, Men | low | low | low | low | Male Mean: 48.9 (2.8)  Female Mean: 50.8 (2.8) |
| Agelink M.W | 1995 | 70 | Germany | Single site | Unclear | Women, Men | unclear | high | low | unclear | Mean: 36.8 (11.6) Range: 21-68 |
| Aithal, G. P | 1998 | 81 | United Kingdom | Single site | English | Women, Men | low | low |  | unclear | Median: 49 Range: 20-85 |
| Allen J | 1999 | 1726 | USA | Multi-site | English | Women, Men | low | low | low | low | Male Mean: 40.6 (10.9)  Female Mean: 40.2 (11.8) |
| Almarri T | 2015 | 106 | United Arab Emirates | Single site | Arabic | Men | low | low |  | low | Mean: 34.2 (8.5) Range: 19-62 |
| Alterman A | 1998 | 1008 | USA | Multi-site | English | Women, Men | high | unclear |  | unclear | Mean: 38 |
| Amaral R | 2004 | 192 | Brazil | Single site | English | Women, Men | low | low | low | low | Not Reported |
| Amoureus M | 1994 | 128 | Netherlands | Single site | English | Men | low | unclear |  | unclear | Mean: 27.6 (5.8) |
| Anton R | 2001 | 648 | USA | Single site | English | Women, Men | high | unclear |  | low | Not Reported |
| Anton, R. F | 2002 | 648 | USA | Multi-site | English | Women, Men | high | low | high | low | Mean: 44 (10) Range: 21+ |
| Anton, RF | 1994 | 60 | Finland, France, USA | Multi-site | English | Women, Men | unclear | low | low | low | Cases Mean: 40 (10)  Controls Mean: 39 (10) |
| Anttila P | 2005 | 136 | Finland | Single site | Other | Women, Men | unclear | low | low | unclear | Cases Mean: 46 (10)  Controls Mean: 55 (16) |
| Anttila P | 2003 | 444 | Finland | Single site | English | Women, Men | unclear | low | low | low | Mean: 48.3 |
| Appleby L | 1997 | 100 | USA | Single site | English | Women, Men | high | low | low | low | Mean: 34 |
| Aradottir S | 2006 | 144 | Sweden | Single site | English | Women, Men | high | low |  | unclear | Outpatients Mean: 49.1 (9.9)  Inpatients Mean: 52.9 (8.5) |
| Arumalla V | 2012 | 100 | India | Single site | English | Men | high | low |  | low | Range: 20-60 |
| Ashman T | 2004 | 223 | USA | Single site | English | Women, Men | high | high | low | low | Mean: 42 (15)  Range: 18-87 |
| Barry A | 2015 | 347 | USA | Multi-site | English | Women, Men | high | low | low | low | Mean: 22 (3.63) |
| Bell H | 1994 | 502 | Norway | Single site | English | Women, Men | low | low | low | high | Not Reported |
| Bell H | 1993 | 420 | Norway | Single site | English | Women, Men | unclear | low | low | high | Not Reported |
| Berg JE | 2011 | 74 | Norway | Single site | Unclear | Other | unclear | unclear | unclear | low | Range: 18-50 |
| Bergman H | 2002 | 997 | Sweden | Multi-site | Unclear | Women, Men | low | low |  | high | Range: 17-71 |
| Berman A | 2005 | 154 | Sweden | Multi-site | Unclear | Women, Men | unclear | high | unclear | unclear | Mean: 34 Range: 18-74 |
| Bernadt, M. W. | 1983 | 112 | United Kingdom | Single site | English | Women, Men | high | low |  | high | Range: 16-65 |
| Bertholet, N. | 2017 | 402 | Switzerland | Single site | English, Spanish | Women, Men | high | low | low | low | Mean: 40 (9.8) |
| Bischof, G | 2007 | 2077 | Germany | Multi-site | Other | Women, Men | high | low | high | low | Range: 18-64 |
| Blank M | 2015 | 5082 | New Zealand | Multi-site | English | Women, Men | low | high | unclear | low | Mean: 22.2 (1.4) |
| Boschloo L | 2010 | 2404 | Netherlands | Multi-site | Other | Women, Men | low | low | low | low | Mean: 41.3 (13) |
| Bradley, K. A | 1998 | 9513 | USA | Multi-site | English | Women, Men | low | low | low | low | Range: 50-70 |
| Bradley, K. A.; | 2003 | 393 | USA | Multi-site | English | Women | high | low | low | low | Range: 50+ |
| Brathen, G. | 2001 | 484 | Norway | Single site | Unclear | Women, Men | high | low | low | low | Not Reported |
| Brochu S | 1995 | 304 | Canada | Single site | French | Women, Men | low | unclear |  | low | Mean: 30.5 |
| Bryce S | 2015 | 113 | Australia | Single site | English | Women, Men | low | high | low | low | Not Reported |
| Bush K | 2015 | 393 | USA | Single site | English | Women | low | low | low | low | Mean: 46 (13.9) |
| Bush B | 1987 | 518 | USA | Single site | English | Women, Men | low | low | high | low | CAGE Positive Mean: 48.9  CAGE Negative Mean: 50.26 |
| Bush K | 1998 | 243 | USA | Multi-site | English | Men | low | high | low | low | Mean: 67 |
| Carey K | 1997 | 97 | USA | Single site | English | Women, Men | high | unclear |  | unclear | Mean: 39.5 (9.1) Range: 22-62 |
| Carey, K | 2003 | 1349 | India | Single site | Unclear | Women, Men | high | unclear |  | unclear | Mean: 33 (10) Range: 18+ |
| Carey, K. B. | 1997 | 79 | USA | Single site | English | Women, Men | high | low |  | low | Mean: 39.9 (8.97) Range: 23-62 |
| Cassidy C | 2008 | 128 | Canada | Single site | English, French | Women, Men | high | low | low | low | Mean: 22.7 Range: 14-30 |
| Castells M | 2005 | 747 | Brazil | Single site | English, Portuguese | Women, Men | low | low | low | low | Mean: 50.4 (17) Range: 18-96 |
| Caviness C | 2009 | 1751 | USA | Single site | English | Women | high | low | low | low | Mean: 33.7 (9.5) Range: 18-62 |
| Chan A | 1994 | 1635 | USA | Single site | English | Women, Men | low | low | low | high | Range: 18-65 |
| Chantarujikapong S | 1997 | 143 | USA | Single site | English | Women | low | high | low | low | Mean: 29.8 (5) Range: 20.4-46.2 |
| Chen V | 2008 | 522 | China | Multi-site | Chinese | Women, Men | unclear | unclear |  | high | Not Reported |
| Chen Y | 2016 | 1009 | China | Multi-site | Chinese | Men | low | high | high | low | Mean: 28.7 (8.8) |
| Chen, Chun-Hsin | 2005 | 422 | China | Single site | Chinese | Women, Men | low | low | low | low | Male Mean: 41.9 (12.9) Range: 18-65  Female Mean: 43.7 (13.9) Range: 18-65 |
| Chermack S | 2000 | 175 | USA | Single site | English | Men | low | unclear | low | low | Mean: 50.4 (6.8) |
| Cherpitel C | 2001 | 3598 | Mexico, USA | Multi-site | English, Spanish | Women, Men | low | high | high | low | Not Reported |
| Cherpitel, C. J | 2000 | 2097 | Mexico, USA | Multi-site | English, Spanish | Women, Men | low | low | low | low | Not Reported |
| Cherpitel, C. J. | 2001 | 1802 | USA | Multi-site | English | Women, Men | low | low | low | low | Not Reported |
| Cherpitel, C. J. | 1997 | 2046 | USA | Multi-site | English | Women, Men | low | low | low | low | Not Reported |
| Cherpitel, C. J. | 1998 | 2870 | USA | Multi-site | English | Women, Men | low | low | low | low | Range: 18-50 |
| Cherpitel, C. J. | 1995 | 1330 | USA | Single site | English | Women, Men | low | low | low | low | Not Reported |
| Cherpitel, C. J. | 1995 | 1330 | USA | Single site | English | Women, Men | low | low | low | low | Not Reported |
| Cherpitel, C. J. | 2002 | 7611 | USA | Multi-site | English, Spanish | Women, Men | low | high |  | high | Not Reported |
| Christensen K | 2005 | 1785 | Denmark | Multi-site | Other | Women, Men | unclear | unclear | low | low | Mean: 38.8 Range: 18-65 |
| Chrostek, L | 2006 | 105 | Poland | Single site | Unclear | Men | high | high |  | low | Mean: 44 Range: 20-65 |
| Clements R | 2002 | 339 | USA | Single site | English | Women, Men | low | high | low | low | Mean: 23.4 (7.7) Median: 20 Range: 18-62 |
| Clements, R. | 1998 | 306 | USA | Single site | English | Women, Men | high | low | low | low | Mean: 25.8 (9.2) Range: 18-55 |
| Cocco K | 1998 | 97 | USA | Single site | English | Women, Men | low | high | low | low | Mean: 37 (12.8) Range: 22-62 |
| Conigrave K | 2002 | 1863 | Australia, Brazil, Canada, Finland, Japan | Multi-site | English, French, Portuguese, Other | Women, Men | low | low | high | low | -- |
| Conley T | 2001 | 126 | USA | Single site | English | Women, Men | low | unclear |  | low | Mean: 38 (10.72) Range: 20-76 |
| Conley T | 2006 | 263 | USA | Multi-site | English | Women, Men | low | unclear |  | high | Mean: 39 (10) Median: 39 Range: 20-78 |
| Conley, Timothy B. | 2005 | 129 | USA | Single site | English | Women, Men | low | low | low | low | Mean: 39 |
| Cook, R. L | 2005 | 358 | USA | Single site | English | Women, Men | high | low | low | low | Mean: 20.6 (2.1) Ranged: 15-24 |
| Cornel M | 1994 | 1405 | Netherlands | Multi-site | Unclear | Women, Men | low | unclear |  | low | Not Reported |
| Corradi-Webster C | 2005 | 127 | Brazil | Single site | Other , Unclear | Women, Men | low | high | high | low | Mean: 42.87 (13.2) Range: 18-77 |
| CortÃ©s-TomÃ¡s M | 2016 | 1001 | Spain | Multi-site | Spanish | Women, Men | low | high | high | low | Range: 15-17 |
| Coulton S | 2006 | 194 | United Kingdom | Multi-site | English, Unclear | Men | low | unclear | low | low | Mean: 46.2 Range: 18.3-80.9 |
| Crawford, E | 2013 | 1775 | USA | Multi-site | English | Women, Men | unclear | high | low | low | Mean: 37 (10) Range: 20-66 |
| Cremonte M | 2010 | 3624 | Argentina, Mexico, USA | Multi-site | English, Spanish | Women, Men | low | high | unclear | high | Not Reported |
| Cremonte M | 2008 | 643 | Argentina | Single site | Spanish | Women, Men | low | low | low | high | Mean: 36 Median: 31 |
| Crowe R | 1997 | 4230 | USA | Multi-site | English | Women, Men | low | high | low | low | Mean: 40 (15) |
| Currie, S | 2004 | 1802 | Canada | Single site | English | Women, Men | high | unclear |  | unclear | Mean: 38.6 (11.2) Range: 18-87 |
| Dawe S | 2000 | 71 | Australia | Single site | English | Women, Men | low | unclear | low | low | Mean: 38 Range: 18-64 |
| Dawson D | 2005 | 43093 | USA | Multi-site | English | Women, Men | low | high | low | low | Not Reported |
| De Feo T | 1999 | 133 | Italy | Single site | English | Women, Men | high | high | high | low | Not Reported |
| De Silva P | 2008 | 150 | Sri Lanka | Multi-site | Other | Men | high | unclear | unclear | low | Mean: 41 (13) Range: 19-70 |
| de Torres, L | 2009 | 614 | Spain | Multi-site | Spanish | Women, Men | low | low | low | low | Mean: 43 (1.43) Range: 18-80 |
| DeJong C | 1995 | 144 | Netherlands | Single site | English | Women, Men | high | unclear |  | unclear | Not Reported |
| DemÄ°rbaÅŸ, H. | 2014 | 115 | Turkey | Single site | Other | Men | low | unclear |  | unclear | Mean: 43 (8.1) Range: 18-65 |
| DeMartini K | 2012 | 401 | USA | Single site | English | Women, Men | high | low | low | low | Mean: 19.04 (1.13) Range: 18-25 |
| do Amaral R | 2008 | 169 | Brazil | Single site | Portuguese | Men | low | unclear | unclear | low | Not Reported |
| Doub T | 2001 | 131 | USA | Single site | English | Women, Men | low | unclear |  | unclear | Mean: 36.4 |
| Doyle S | 2009 | 1726 | USA | Multi-site | English | Women, Men | low | unclear |  | unclear | Mean: 40.3 (11) |
| Drake R | 1990 | 75 | USA | Single site | English | Women, Men | high | low | unclear | low | Mean: 43.6 (14.3) |
| Durbeej N | 2010 | 181 | Sweden | Multi-site | Other | Women, Men | low | high | unclear | unclear | Mean: 33 (10.88) Range: 17-60 |
| Dyson V | 1998 | 100 | USA | Single site | English | Women, Men | low | low | low | high | Mean: 33.6 |
| Evren C | 2014 | 202 | Turkey | Single site | Other | Women, Men | high | low |  | low | No Drug Use Disorder Mean: 38.14 (9.44)  Drug Use Disorder Mean: 34.34 (9.28) |
| Evren C | 2013 | 258 | Turkey | Single site | Other | Women, Men | low | high | low | unclear | Not Reported |
| Fabbri, C. E | 2007 | 450 | Brazil | Single site | Portuguese | Women | high | low | high | low | Range: 20-29 |
| Fagan K | 2014 | 52 | Australia | Single site | English | Women, Men | low | low | low | low | Mean: 50.3 (11.8) |
| Fagerberg B | 1994 | 439 | Sweden | Single site | Other | Men | low | low | unclear | high | Range: 54-77 |
| Fals-Stewart W | 2000 | 113 | USA | Single site | English | Women, Men | low | unclear | high | low | Mean: 27.4 (6.3) |
| Fiellin D | 2013 | 3131 | USA | Multi-site | English | Women, Men | low | low | unclear | low | Mean: 49 |
| Fillmore M | 2011 | 251 | USA | Single site | English | Women, Men | low | low | low | low | Mean: 26.5 (4.6) Range: 19-34 |
| Fleming M | 1991 | 989 | USA | Single site | English | Women, Men | low | low | low | low | Mean: 20.5 |
| Fleming M | 2004 | 799 | USA | Multi-site | English | Women, Men | low | low | low | low | Mean: 49.7 |
| Forsberg L | 2002 | 149 | Sweden | Single site | Other | Women, Men | high | high | high | low | Range: 16-73 |
| Foxcroft, David R | 2015 | 420 | United Kingdom | Multi-site | English | Women, Men | high | low | low | low | Range: 18-35 |
| Frank D | 2008 | 1292 | USA | Single site | English, Spanish | Women, Men | low | low | low | low | Mean: 43 |
| Fulbrook P | 2015 | 637 | Australia | Single site | English | Women, Men | low | low | low | low | Mean: 49.9 (20) Range: 18-92 |
| Fuller M | 1994 | 10 | USA | Single site | English | Women, Men | low | unclear | low | low | Cases Mean: 36.8 (4.09)  Controls Mean: 36.2 (8.64) |
| GÃ?mez A | 2006 | 602 | Spain | Multi-site | Spanish | Women, Men | low | unclear | unclear | low | Older Group Mean: 72 (6.1) Range: 65+  Younger Group Mean: 38 (18) |
| Gache P | 2005 | 1207 | France, Switzerland | Multi-site | French | Women, Men | low | high | unclear | low | Mean: 43.2 (17.18) |
| Gammeter R | 2006 | 219 | Switzerland | Single site | French | Women, Men | high | low | low | low | Mean: 37.8 (12.4) Range: 18-64 |
| Garcia Carretero M | 2016 | 1309 | Spain | Single site | Spanish | Women, Men | low | unclear | high | low | Mean: 21.78 (4.45) Range: 18-65 |
| Garzotto N | 1988 | 150 | Italy | Multi-site | Other | Women, Men | low | high | unclear | low | Not Reported |
| Gates, T | 2007 | 411 | USA | Single site | English | Men | unclear | low | low | low | Mean: 27 Range: 20-34 |
| Gavin, D. R | 1989 | 501 | Canada | Single site | English | Women, Men | low | low | low | low | Mean: 34.7 (10.9) |
| Geneste J | 2012 | 164 | France | Single site | French | Women, Men | low | high | unclear | low | Mean: 46 (11.6) |
| Giang K | 2005 | 485 | Viet Nam | Single site | Other | Women, Men | low | high | high | low | Male Mean: 41.9 (10.9)  Female Mean: 40.4 (10.9) |
| Gijsbers A | 1991 | 86 | Australia | Single site | English | Men | low | low | unclear | low | Mean: 23.1 Median: 21 (21-25) Range: 19-31 |
| Girela E | 1994 | 121 | Spain | Multi-site | Spanish | Women, Men | unclear | unclear | unclear | low | Range: 17-81 |
| Godart B | 2005 | 633 | France | Single site | English | Women, Men | high | low | high | low | Male Mean: 42 (10)  Female Mean: 45 (10) |
| Gomez A | 2001 | 179 | Spain | Single site | Spanish | Women, Men | low | unclear | unclear | low | Cases Mean: 49 (12)  Controls Mean: 49 (15) |
| GonzÃ¡lez-SÃ¡iz | 2009 | 135 | Spain | Multi-site | Spanish | Women, Men | high | low | low | low | Range: 18-30 |
| Gossop, M. | 2002 | 735 | United Kingdom | Single site | English | Women, Men | low | low |  | low | Mean: 29.4 |
| Gough G | 2015 | 482 | USA | Single site | English | Women, Men | high | low | low | low | Cases Mean: 39.7 (11.8)  Controls Mean: 32.5 (8.6) |
| Grekin E | 2010 | 300 | USA | Single site | English | Women | low | low | low | low | Not Reported |
| Gual A | 2002 | 255 | Spain | Multi-site | Spanish | Women, Men | high | low | low | low | Male Mean: 43.6 (13.1) Range: 17-82  Female Mean: 44.4 (14.4) Range: 18-81 |
| Gul S | 2005 | 85 | Turkey | Single site | Other | Men | low | unclear | unclear | low | Not Reported |
| Gundersen O | 2013 | 256 | Norway | Single site | English | Women, Men | high | low | low | high | Not Reported |
| Guo W | 2008 | 3171 | Country is not listed | Multi-site | Other | Women, Men | low | low | low | low | Mean: 43.8 (15.6) Range: 15-89 |
| Gureje O | 1992 | 214 | Nigeria | Single site | Other | Women, Men | high | low | low | low | Range: 18-50 |
| Hahn J | 2012 | 77 | Uganda | Single site | English, Other | Women, Men | high | low | low | low | Median: 32 (26-38) |
| Haller D | 2010 | 42 | USA | Multi-site | English | Women, Men | high | low | low | high | Mean: 47.9 (9.7) |
| Hallinan P | 2011 | 2005 | Ireland | Multi-site | English | Women, Men | low | low |  | low | Mean: 31 (9.7) Range: 13-70 |
| Halm U | 1999 | 72 | Germany | Single site | English | Women, Men | high | unclear |  | unclear | Median: 54 Range: 25-77 |
| Hannuksela, M | 1992 | 90 | Finland | Single site | English | Men | high | low |  | low | Cases Mean: 39.8 (9)  Controls  Mean: 36.9 (11) |
| Hastedt M | 2013 | 169 | Germany | Single site | English | Women, Men | high | low | low | low | Not Reported |
| Hays R | 1995 | 832 | USA | Multi-site | English | Women, Men | high | unclear |  | low | Mean: 33 Range: 18-71 |
| Hays, R. D | 2001 | 103 | USA | Single site | English | Women, Men | unclear | low | low | low | Not Reported |
| Hazelett S | 1998 | 182 | USA | Single site | English | Women, Men | low | low | low | low | Not Reported |
| Heck E | 1995 | 511 | USA | Single site | English | Women, Men | low | low | low | low | 1988 Study Mean: 21.6 Range: 17-50  1992 Study Mean: 23 Range: 17-50 |
| Hendriks V | 1989 | 142 | Netherlands | Single site | Other | Women, Men | low | high |  | low | Mean: 27.8 (5.5) |
| Hides L | 2009 | 214 | Australia | Single site | English | Women, Men | high | low | low | low | Mean: 19.81 (2.85) Range: 15-25 |
| Hildebrand M | 2015 | 371 | Netherlands | Single site | Other | Women, Men | high | high |  | unclear | Mean: 34.3 (12.2) Range: 18-69 |
| Hill, Kevin P | 2007 | 50 | USA | Single site | English | Women, Men | high | low | low | low | Mean: 40.2 |
| Hinkin C | 2001 | 976 | USA | Single site | English | Women, Men | high | low | low | low | Mean: 70.6 (7.7) Range: 60-96 |
| Hirata E | 2001 | 122 | Brazil | Single site | Portuguese | Men | low | high | unclear | low | Cases Mean: 70.69  Controls Mean: 72.61 |
| Hock B | 2005 | 364 | Germany | Single site | English | Women, Men | high | high | high | low | Not Reported |
| Hodgins D | 2002 | 152 | Canada | Single site | English | Women, Men | high | high | low | low | Mean: 36.7 (9.4) |
| Hodgson, R. J. | 2003 | 2185 | United Kingdom | Multi-site | English | Women, Men | low | high | low | low | Range: 16-75 |
| Humeniuk R | 2014 | 1047 | Spain | Multi-site | Spanish | Women, Men | low | unclear |  | unclear | Mean: 30.4 (8.2) |
| Huseby N | 1997 | 57 | Germany | Single site | English | Women, Men | low | low | low | low | Male Median: 45.5 Range: 20-91  Female Median: 48.5 Range: 20-91 |
| Huseby N | 1997 | 339 | Germany | Single site | Other | Women, Men | unclear | low | unclear | high | Range: 20-91 |
| Indran S | 1995 | 621 | Malaysia | Single site | Chinese, Other | Women, Men | low | unclear | low | high | Not Reported |
| Iraurgi C | 2010 | 315 | Spain | Multi-site | Spanish | Women, Men | high | low | low | high | Mean: 34.1 (5.2) Range: 19-57 |
| Ireland J | 2011 | 300 | USA | Multi-site | English | Women, Men | unclear | low | low | low | Mean: 44 (8) |
| JÃ?zsef G | 2005 | 266 | Hungary | Single site | Other | Women, Men | high | unclear |  | unclear | Mean: 26.7 (9.1) Range: 16-65 |
| Jain J | 2014 | 137 | USA | Single site | English | Women, Men | low | low | low | low | Median: 25.6 (22.8-27.7) |
| Jayasekera H | 2011 | 329 | Australia | Single site | English | Women, Men | low | unclear | low | low | Mean: 36 (0.75) |
| Johnson T | 2005 | 120 | USA | Multi-site | English | Women | low | unclear |  | high | Not Reported |
| Johnson, J. | 2013 | 625 | USA | Single site | English | Women, Men | high | low | low | low | Male Mean: 42 (13.7)  Female Mean: 40 (12.8) |
| Joseph C | 1995 | 117 | USA | Single site | English | Women, Men | high | low | low | low | Mean: 69 (8.6) Range: 51-98 |
| Kaarne, Tiina | 2010 | 759 | Finland | Single site | Unclear | Women, Men | low | high | high | low | Male Mean: 45.7  Female Mean: 46.3 |
| Kader R | 2012 | 43 | South Africa | Single site | English, Other | Women, Men | low | unclear | low | high | Mean: 34 Range: 23-59 |
| Källmén H | 2015 | 849 | Sweden | Multi-site | English | Women, Men | low | unclear |  | high | Range: 17-71 |
| Kanitz R | 1994 | 162 | Germany | Single site | Other | Women, Men | low | low | high | low | Not Reported |
| Kavanagh, D. J | 2011 | 118 | Australia | Single site | English | Women, Men | low | low | low | low | Early Episodic Sample Mean: 24.8 (5.3) Median: 25 Range: 17-39  Forensic Sample Mean: 31.2 (10.1) Range: 17-60 |
| Kawada T | 2011 | 113 | Japan | Single site | Japanese | Men | unclear | unclear |  | unclear | Mean: 38.2 (9.9) Range: 20-67 |
| Kechagias S | 2015 | 44 | Sweden | Single site | Other | Women, Men | low | high | unclear | low | Alcohol Drinkers Mean: 33 (9)  Teetotalers Mean: 34 (9) |
| Kelly T | 2002 | 103 | USA | Multi-site | English | Women, Men | low | unclear |  | low | Mean: 17.5 (2.1) Range: 12.2-20.9 |
| Kelly T | 2004 | 93 | USA | Multi-site | English | Women, Men | low | high | low | low | Mean: 19 (0.9) Range: 18-20 |
| Kelly T | 2009 | 181 | USA | Multi-site | English | Women, Men | low | high | low | high | Range: 18-20.9 |
| Kessler R | 2009 | 694 | USA | Multi-site | English | Women, Men | low | unclear | unclear | low | Not Reported |
| Khan R | 2012 | 100 | Switzerland | Multi-site | French | Women, Men | low | unclear |  | unclear | Mean: 77.8 (7.5) |
| Khan R | 2011 | 150 | Switzerland | Multi-site | French | Women, Men | low | unclear | unclear | low | Mean: 41 (11.5) |
| Kim J | 2013 | 497 | South Korea (ROK) | Single site | Other | Men | low | high |  | low | Range: 20-64 |
| Kim S | 2008 | 118 | USA | Multi-site | Other | Men | low | low | unclear | high | Mean: 42.11 (10.42) Range: 20-63 |
| Knibbe R | 2006 | 9 | Czech Republic, Finland, Hungary, Iceland, Netherlands, Spain, Sweden, Switzerland, United Kingdom | Multi-site | English | Women, Men | low | unclear |  | low | Range: 15-64 |
| Knight J | 2002 | 538 | USA | Single site | English | Women, Men | high | low | low | low | Range: 14-18 |
| Knight J | 2003 | 538 | USA | Single site | English | Women, Men | high | low | low | low | Range: 14-18 |
| Kokotailo P | 2004 | 302 | USA | Single site | English | Women, Men | high | low | low | low | Mean: 20.3 Range: 18-23 |
| Koppes L | 2004 | 331 | Netherlands | Single site | English | Women, Men | high | low | low | low | Mean: 36.1 (0.7) |
| Korzec A | 2005 | 114 | Netherlands | Single site | English | Women, Men | high | unclear | unclear | high | Range: 24-80 |
| Korzec S | 2009 | 1250 | Australia, Brazil, Canada, Finland, Japan | Multi-site | English | Men | high | low | low | low | Not Reported |
| Kowalyszyn M | 2003 | 99 | Australia | Multi-site | English | Women, Men | high | low |  | unclear | Mean: 32.9 Range: 16-60 |
| Krenz S | 2004 | 54 | Switzerland | Single site | French | Women, Men | high | unclear |  | unclear | Mean: 29.7 (6.21) |
| Kuo C | 1999 | 198 | China | Single site | Chinese | Women, Men | low | high | unclear | high | Not Reported |
| Kwak H | 2014 | 305 | Country is not listed | Single site | English | Women | high | high | high | low | Not Reported |
| Kwoh-Gain I | 1990 | 63 | Australia | Multi-site | English | Women, Men | low | high | unclear | low | Not Reported |
| La Grange L | 1994 | 148 | USA | Single site | Unclear | Women, Men | high | low | low | high | Male Mean: 25.5 Range: 18-45  Female Mean: 23.7 Range: 18-49 |
| Lam L | 2015 | 1214 | China | Multi-site | Chinese | Women | high | high | high | high | Mean: 31 |
| Lapham G | 2014 | 18403 | USA | Multi-site | English | Women, Men | low | low | low | low | Not Reported |
| Latimer W | 1997 | 342 | USA | Multi-site | English | Women, Men | high | low | low | low | Range: 12-19 |
| Laux J | 2015 | 230 | USA | Single site | English | Women, Men | high | unclear |  | low | Mean: 28.1 (10.4) Median: 26 Range: 18-59 |
| Lawrinson, P | 2007 | 90 | Australia | Single site | English | Women, Men | high | low | low | low | Mean: 28.5 (10.3) Range: 15-56 |
| Lees R | 2012 | 100 | USA | Single site | English | Women, Men | low | low | high | low | Not Reported |
| Lehman, A. F | 1996 | 435 | USA | Multi-site | English | Women, Men | high | low | low | low | Mean: 31.5 Median: 33.2 Range: 18-64 |
| Lesch O | 1996 | 101 | Austria | Single site | English | Women, Men | unclear | low | low | low | Mean: 59.8 (14.4) Median: 61 |
| Levola J | 2015 | 390 | Finland | Multi-site | Other | Women, Men | low | low | low | low | Not Reported |
| Liangpunsakul S | 2010 | 8708 | USA | Multi-site | English | Women, Men | low | unclear |  | unclear | Not Reported |
| Lima C | 2015 | 166 | Brazil | Multi-site | Portuguese | Women, Men | low | low | low | low | Range: 18-60 |
| Liskow B | 1995 | 662 | USA | Single site | English | Men | high | low | low | low | Mean: 42.6 (11.7) |
| Lopes Cde S | 1994 | 30 | Brazil | Single site | Portuguese | Women, Men | high | low | low | low | Range: 18-40 |
| Lundin A | 2015 | 1086 | Sweden | Multi-site | English | Women, Men | unclear | low | low | low | Range: 20-64 |
| Luo W | 2011 | 526 | China | Multi-site | Chinese | Women, Men | low | unclear |  | unclear | Mean: 37.8 (6) Range: 21-53 |
| Luttrell S | 1997 | 162 | United Kingdom | Multi-site | English | Women, Men | low | low | low | low | Mean: 78 Range: 65-99 |
| MacKenzie, D | 1996 | 240 | United Kingdom | Single site | English | Women, Men | high | low | low | low | Male Mean: 54 (18.8) Range: 17-91  Female Mean: 60.1 (19.7) |
| Madhubala, V. | 2013 | 50 | India | Single site | Unclear | Men | high | low | high | low | Cases Mean: 38.66 (10.2) Range: 20-60  Controls Mean: 38.12 (9.8) |
| Magruder H | 1993 | 915 | USA | Single site | English | Men | high | low | low | low | Mean: 54.4 |
| Maisto, S | 2000 | 7035 | USA | Multi-site | English | Women, Men | high | unclear |  | unclear | Range: 41-50 |
| Maisto, S. A | 2000 | 162 | USA | Single site | English | Women, Men | low | low | low | low | Mean: 38 (9) Range: 18-66 |
| Malet L | 2005 | 5452 | France | Multi-site | French | Women, Men | high | high | high | low | Male Mean: 59 (17)  Female Mean: 63 (21) |
| Masur, J. | 1983 | 114 | Brazil | Single site | Portuguese | Men | high | low | low | low | Problem Drinkers Median: 42 Range: 22-69  Psychiatric Patients Median: 31 Range: 18-78 |
| Matano, R. A | 2003 | 228 | USA | Single site | English | Women, Men | high | low | high | low | Mean: 40.5 (11.2) |
| Matthews, J. C | 2010 | 254 | Russia | Single site | Unclear | Women, Men | low | unclear | unclear | low | Mean: 40.2 (11.4) |
| Matuszka B | 2014 | 368 | Hungary | Multi-site | Other | Women, Men | low | unclear |  | low | Not Reported |
| McCambridge J | 2009 | 167 | United Kingdom | Multi-site | English | Women, Men | low | high | low | low | Mean: 20.3 (2.2) |
| McCann, B. S | 2000 | 139 | USA | Single site | English | Women, Men | high | low | low | low | Mean: 36.4 (10.5) Range: 18-64 |
| McDonald H | 2013 | 1023 | Russia | Multi-site | English | Men | low | unclear |  | unclear | Range: 27-59 |
| McGinnis, K. A | 2013 | 837 | USA | Multi-site | English | Men | low | low | low | low | Mean: 52 |
| McNeely J | 2014 | 101 | USA | Single site | English, Spanish | Women, Men | high | unclear |  | unclear | Mean: 46 (10) Median: 47 (14) Range: 19-64 |
| McQuade, W. H | 2000 | 300 | USA | Single site | English | Women, Men | low | low | low | low | Range: 18-45 |
| Meerkerk G | 1999 | 524 | Netherlands | Multi-site | Other | Men | low | low | unclear | high | Mean: 55 |
| Meneses-Gaya | 2010 | 999 | Brazil | Multi-site | Portuguese | Women, Men | high | low | low | low | Mean: 23 (6) |
| Meneses-Gaya C | 2010 | 530 | Brazil | Single site | Portuguese | Women, Men | low | low | unclear | low | Mean: 36 (13) |
| Meregalli M | 1995 | 191 | Italy | Single site | Other | Women, Men | low | low | unclear | low | Not Reported |
| Merikallio-Pajunen, A | 2004 | 259 | Finland | Single site | Other | Women, Men | high | low | low | low | Not Reported |
| Midanik, L. T | 1998 | 1147 | USA | Multi-site | English | Women | high | low | low | low | Range: 15-19 |
| Mikkelsen I | 1998 | 161 | Germany | Single site | Other | Men | unclear | low | unclear | unclear | Alcohol Dependent Median: 45  Non-Dependent Median: 43.5 |
| Mischke, H. D | 1987 | 718 | USA | Single site | English | Women, Men | high | high | high | low | Not Reported |
| Mitchell S | 2014 | 525 | USA | Single site | English | Women, Men | low | high | unclear | low | Range: 12-17 |
| Moore, Alison A | 2002 | 166 | USA | Single site | English | Women, Men | low | low | low | low | Mean: 74.4 Range: 60-97 |
| Moraes, C. L | 2005 | 786 | Brazil | Multi-site | Portuguese | Women | high | high |  | unclear | Mean: 23.6 (6.3) |
| Morini L | 2011 | 76 | Italy | Multi-site | Other | Women, Men | low | low | unclear | high | Cases Mean: 42.4 (12)  Controls Mean: 42.8 (10.8) |
| Morini L | 2009 | 98 | Italy | Multi-site | English | Women, Men | high | low | low | low | EDI over 60 g/day Median: 45 (40-54)  EDI under 60 g/day Median: 41 (31-52) |
| Morini L | 2009 | 86 | Italy | Single site | Other | Women, Men | low | low | unclear | high | Not Reported |
| Morojele N | 2015 | 406 | South Africa | Multi-site | English | Women, Men | high | unclear |  | unclear | Mean: 30 (8.45) |
| Morton, J | 1996 | 120 | USA | Multi-site | English | Men | high | low | low | low | Mean: 71.54 (4.76) |
| Mundle G | 2000 | 126 | Germany | Single site | Other | Women, Men | low | low | unclear | unclear | Male Mean: 40.8 (9.4) Range: 24-62  Female Mean: 44.7 (9.5) Range: 29-67 |
| Mundle G | 1999 | 144 | Germany | Single site | Other | Men | low | low | unclear | unclear | Cases Mean: 41.3 Range: 26-62  Controls Mean: 42.4 Range: 25-70 |
| Myer, L | 2008 | 465 | South Africa | Multi-site | Other | Women, Men | high | low | low | low | Mean: 33 |
| Myerholtz L | 1997 | 46 | USA | Multi-site | English | Women, Men | high | unclear |  | high | Mean: 31 (9.71) Range: 19-67 |
| Naik P | 1995 | 58 | United Kingdom | Single site | English | Women, Men | low | low | high | low | Mean: 77.1 Range: 66-94 |
| Nalpas B | 1989 | 303 | France | Multi-site | English | Women, Men | low | low | low | low | Mean: 55 (17) |
| Nesvag R | 2010 | 205 | Norway | Multi-site | English | Women, Men | high | low |  | low | Male Mean: 27.5 (7.9) Range: 18-65 |
| Neumann T | 2008 | 81 | Germany | Single site | Other | Men | low | low | unclear | low | Median: 35 |
| Neumann T | 2012 | 1556 | Germany | Multi-site | Other | Women, Men | low | high |  | low | Male Median: 50 (35-63)  Female Median: 45 (33-60) |
| Neumann T | 2004 | 1927 | Germany | Single site | Other | Women, Men | low | high | unclear | low | Male Median: 32 (25-40)  Female Median: 32 (24-44) |
| Neumann, T | 2009 | 1233 | Germany | Single site | Other | Women, Men | low | low | low | low | Not Reported |
| Niemela O | 1995 | 373 | Canada | Single site | English | Women, Men | high | unclear |  | low | Not Reported |
| Nystrom M | 1993 | 2370 | Finland | Multi-site | Other | Women, Men | low | unclear | unclear | low | Mean: 22.2 |
| Nystrom M | 1992 | 289 | Finland | Single site | Other | Women, Men | low | low | unclear | low | Mean: 22.1 |
| O'Hare T | 1997 | 394 | USA | Single site | English | Women, Men | low | low |  | low | Mean: 20.4 (2.5) |
| O'Hare T | 1999 | 312 | USA | Single site | English | Women, Men | low | high |  | low | Mean: 18.6 (0.87) Range: 18-23 |
| O'Hare T | 2010 | 149 | USA | Single site | English | Women, Men | low | high | unclear | low | Mean: 42.7 (10.2) Median: 43 |
| Oslin D | 1998 | 88 | USA | Single site | English | Women, Men | low | unclear | low | low | Male Mean: 42.9 (9.9)  Female Mean: 46.5 (10.6) |
| Pal H | 2004 | 297 | India | Multi-site | Unclear | Women, Men | low | high | unclear | low | Mean: 38.1 (9.6) |
| Philpot M | 2003 | 128 | United Kingdom | Single site | English | Women, Men | low | low | low | low | Mean: 77.1 (6.7) |
| Piano, S | 2014 | 121 | USA | Single site | English | Women, Men | high | low | low | low | Mean: 56.5 (9.2) |
| Piccinelli, M | 1997 | 482 | Italy | Single site | English, Other | Women, Men | high | low | low | low | Mean: 42.2 (14.4) |
| Pierucci L | 2005 | 293 | USA | Multi-site | English | Women, Men | high | unclear |  | high | Mean: 37.8 |
| Pirro V | 2011 | 175 | Italy | Multi-site | English | Women, Men | high | low | low | low | Mean: 44.6 Range: 22-74 |
| Plackett T | 2015 | 222 | USA | Single site | English | Women, Men | low | low | low | low | Mean: 40.1 (16.7) |
| Pradhan B | 2012 | 944 | Nepal | Single site | Other | Women, Men | low | high | unclear | high | Mean: 47.7 (11.9) |
| Radosavljevic M | 1995 | 196 | Austria | Single site | Other | Women, Men | low | unclear | high | low | Male Mean: 55 (2) Range: 15-79  Female Mean: 55 (2) Range: 15-85 |
| Reid M | 2005 | 587 | USA | Multi-site | English | Men | high | low | low | low | Mean: 74.5 (5.3) |
| Reynaud M | 2000 | 268 | France | Multi-site | French | Women, Men | low | low | low | low | Not Reported |
| Reynaud M | 1998 | 70 | France | Single site | French | Women, Men | low | low | low | low | Cases Range: 22-58  Controls Range: 24-60 |
| Richoux C | 2011 | 1079 | France | Multi-site | English | Women, Men | low | low | low | low | Mean: 46.6 (20) Range: 18-94 |
| Rikoon S | 2006 | 2824 | USA | Single site | English | Women, Men | low | high | high | low | Mean: 35 (10) |
| Rinck D | 2007 | 358 | Germany | Multi-site | Other | Women, Men | unclear | low | unclear | low | Cases Mean: 44 Range: 22-67  Controls Mean: 30 Range: 19-87 |
| Rodriguez-Martos A | 2007 | 120 | Spain | Single site | Spanish | Women, Men | low | unclear | unclear | low | Mean: 31 Median: 27 (22-34) |
| Rosenberg, S. D | 1998 | 350 | USA | Single site | English | Women, Men | low | low | low | low | Mean: 38.03 (8.82) |
| Ross H | 1994 | 110 | Canada | Single site | English | Women, Men | low | high | low | low | Median: 23 Range: 18-48 |
| Rubio V | 2014 | 485 | Spain | Multi-site | Spanish | Women, Men | low | low | low | low | Mean: 49.9 (18.85) |
| Rublo M | 1997 | 179 | Spain | Multi-site | Spanish | Men | low | low | unclear | low | Not Reported |
| Rumpf H | 2013 | 225 | Germany | Multi-site | Other | Women, Men | low | high | low | low | Mean: 15.4 (0.81) Range: 14-18 |
| Rumpf H | 2002 | 3551 | Germany | Multi-site | Other | Women, Men | low | unclear | low | low | Mean: 41.2 (12.8) |
| Rumpf H | 1998 | 1379 | Germany | Single site | Other | Women, Men | high | high | high | low | Not Reported |
| Russell M | 1994 | 4743 | USA | Single site | English | Women | high | low | low | low | Mean: 27.9 (5.6) |
| Russell M | 1996 | 2717 | USA | Single site | English | Women | high | low | low | low | Risk Drinkers Mean: 28 (5.4)  Controls Mean: 24.2 (6) |
| Ryb G | 1999 | 684 | USA | Single site | English | Men | low | low | low | low | Mean: 35.2 Range: 18+ |
| Ryou Y | 2012 | 242 | Country is not listed | Single site | Other | Men | high | high | high | low | Mean: 71.8 (5.5) Range: 65+ |
| Sacks J | 2003 | 1026 | USA | Multi-site | English | Women, Men | low | unclear |  | high | Mean: 40.5 (9.7) |
| Saitz R | 2014 | 286 | USA | Single site | English | Women, Men | high | low | low | low | Mean: 49 (12) Range: 21-86 |
| Saitz, R | 1999 | 210 | USA | Single site | English, Spanish | Women, Men | high | low | low | low | Mean: 44 (13) |
| Saitz, R.; Cheng, | 2012 | 286 | USA | Single site | English | Women, Men | high | high | high | low | Mean: 49 (12) Range: 21-86 |
| Santis R | 2009 | 95 | Chile | Single site | Spanish | Women, Men | low | high | low | high | Mean: 15.9 (1.2) |
| Sarkar M | 2010 | 175 | Canada | Single site | English | Women | low | low | high | low | Problem Drinkers Mean: 27.5 (6.3)  Non-Problem Drinkers Mean: 29.1 (5.8) |
| Savola O | 2004 | 349 | Finland | Single site | Other | Women, Men | low | high | unclear | high | Mean: 31 (10) |
| Schellenberg F | 1989 | 210 | France | Single site | English | Women, Men | high | unclear |  | unclear | Cases Range: 18-64  Controls Range: 22-67 |
| Schmidt L | 1997 | 101 | Germany | Single site | Other | Men | low | low | high | low | Mean: 44.5 (8.4) |
| Schmitt U | 1998 | 405 | Germany | Multi-site | Other | Women, Men | low | unclear | high | unclear | Not Reported |
| Schoniger H | 2006 | 110 | Austria | Single site | English | Women, Men | high | unclear |  | unclear | Median: 59 Range: 23-87 |
| Schwan R | 2004 | 362 | France | Multi-site | French | Women, Men | low | unclear | low | low | Mean: 44.4 |
| Seale J | 2006 | 625 | USA | Multi-site | English | Women, Men | unclear | high | low | high | Male Mean: 42.1 (13.7) Median: 41  Female Mean: 40.4 (12.8) Median: 40 |
| Searle A | 2015 | 50049 | Australia | Single site | English | Women, Men | high | low | low | low | Mean: 33.2 (9.2) |
| Selin K | 2003 | 457 | Sweden | Multi-site | Other | Women, Men | low | low |  | high | Not Reported |
| Seth P | 2015 | 639 | Namibia | Multi-site | English, Other | Women, Men | low | unclear | unclear | high | Mean: 30.1 Range: 18-80 |
| Sharpe P | 1996 | 85 | United Kingdom | Multi-site | English | Women, Men | low | high | unclear | low | Not Reported |
| Sheridan D | 1995 | 278 | USA | Single site | English | Women, Men | high | unclear | high | low | Not Reported |
| Siegfried N | 2001 | 395 | South Africa | Multi-site | Other | Women, Men | high | high | high | high | Range: 16+ |
| Sillanaukee P | 1999 | 115 | Finland | Multi-site | Other | Women, Men | low | low | unclear | low | Not Reported |
| Sillanaukee P | 1998 | 465 | Finland | Multi-site | Other | Women, Men | low | low | unclear | unclear | Male Mean: 42 Range: 20-60  Female Mean: 40 Range: 20-60 |
| Sillanaukee, P | 2000 | 6962 | Finland | Multi-site | English | Women, Men | low | unclear |  | unclear | Range: 25-74 |
| Sinadinovic K | 2010 | 2361 | Sweden | Multi-site | Other | Women, Men | unclear | unclear |  | high | Male Mean: 23 (10) Range: 15-86  Female Mean: 23 (10) Range: 15-88 |
| Sinadinovic K | 2011 | 1861 | Sweden | Multi-site | Other | Women, Men | low | unclear |  | low | Male Mean: 46 (18) Range: 16-80  Female Mean: 45 (17) Range: 16-80 |
| Skinner H | 1982 | 223 | USA | Single site | English | Women, Men | low | low |  | low | Mean: 32.47 (11.17) |
| Skinner H | 1984 | 197 | Canada | Multi-site | English | Women, Men | low | low | high | unclear | Not Reported |
| Skipsey K | 1997 | 82 | USA | Single site | English | Women, Men | low | low | low | low | Range: 18-52 |
| Smith D | 1987 | 845 | USA | Single site | English | Women, Men | low | low | high | unclear | Not Reported |
| Smith P | 2009 | 286 | USA | Single site | English | Women, Men | low | low | high | low | Mean: 49 (12.3) Median: 49 Range: 21-86 |
| Smith, P. C | 2010 | 286 | USA | Single site | English | Women, Men | low | unclear | low | low | Mean: 49 (12.3) Median: 49 Range: 21-86 |
| Soderstrom C | 1997 | 1118 | USA | Single site | English | Women, Men | low | high | high | high | Male Mean: 35.5  Female Mean: 42.3 |
| Soderstrom C | 1998 | 1216 | USA | Single site | English | Women, Men | low | low | high | high | Not Reported |
| Sokol R | 1989 | 971 | USA | Single site | English | Women | low | high | unclear | low | Mean: 23.9 (6) |
| Sorvajarvi K | 1996 | 172 | Finland | Single site | English | Women, Men | high | unclear |  | unclear | Not Reported |
| Spies C | 1995 | 105 | Germany | Multi-site | Other | Men | unclear | low | low | high | Alcoholics Mean: 44 (2)  Non-alcoholics Mean: 41 (3) |
| Staines G | 2001 | 248 | USA | Single site | English | Women, Men | low | low | low | high | Mean: 40 Range: 19-72 |
| Staley D | 1990 | 250 | Canada | Single site | English | Women, Men | low | high | low | low | Mean: 37.9 (12.06) |
| Stauber R | 1995 | 199 | Austria | Single site | Other | Women, Men | low | low | low | low | Not Reported |
| Steinbauer, J. R | 1998 | 1333 | USA | Single site | English, Spanish | Women, Men | low | low | low | low | Mean: 43.2 (15.7) Range: 18-86 |
| Stewart S | 2014 | 222 | USA | Single site | English | Women, Men | high | low | low | low | Not Reported |
| Stowell L | 1997 | 57 | New Zealand | Single site | English | Men | low | low | unclear | unclear | Not Reported |
| Strauss S | 2009 | 400 | USA | Single site | English | Women, Men | high | low | low | low | Not Reported |
| Subramaniam, M. | 2010 | 23248 | Singapore | Single site | English | Women, Men | low | low | low | low | Mean: 19 (1.2) Range: 16-26 |
| Svanum S | 1995 | 495 | USA | Single site | English | Women, Men | unclear | unclear | low | low | Mean: 24 (7) Median: 21 Range: 17-70 |
| Svikis, Dace S | 1996 | 80 | USA | Single site | English | Women | low | low | low | low | Mean: 31.8 |
| Taracha E | 2002 | 45 | Poland | Single site | Other | Men | unclear | high | unclear | unclear | Cases Mean: 38.4 (5.1) Range: 23-45  Controls Mean: 36.7 (7) Range: 23-49 |
| Taylor P | 2016 | 219 | USA | Single site | English | Women, Men | low | low | low | low | Mean: 20 Range: 18-35 |
| Teitelbaum, L. M.; | 2000 | 135 | USA | Single site | English | Women, Men | high | low |  | low | Not Reported |
| Thiesen | 2010 | 111 | Denmark | Single site | Other | Women, Men | low | low | unclear | low | Mean: 43.4 Range: 23-74 |
| Thomas E | 2014 | 1325 | Australia | Multi-site | English | Women, Men | low | low | high | high | Not Reported |
| Thomas, Bonnita A.; | 2008 | 167 | United Kingdom | Single site | English | Women, Men | high | unclear |  | unclear | Mean: 20.3 Range: 16-24 |
| Tiburcio Sainz M | 2016 | 1176 | Mexico | Single site | Spanish | Women, Men | low | high | unclear | low | Not Reported |
| Tsai M | 2005 | 112 | China | Multi-site | Chinese | Women, Men | low | high | unclear | low | Mean: 49.9 (15.9) Range: 20-86 |
| Vickers-Douglas K | 2005 | 155 | USA | Multi-site | English | Women, Men | low | unclear |  | low | Mean: 43.3 (11.5) Median: 43 Range: 20-75.4 |
| Villalobos-Gallegos L | 2015 | 565 | Mexico | Multi-site | Spanish | Women, Men | low | unclear | unclear | low | Mean: 30.3 (10.9) |
| Vinson D | 2004 | 2800 | USA | Multi-site | English | Women, Men | high | low | low | high | Missouri Network Mean: 43 (18) IQR: 27-53  AAFP National Network Mean: 52 (17) IQR: 39-64 |
| Vitesnikova J | 2014 | 146 | Australia | Single site | English | Women, Men | low | high | unclear | low | Median: 42 (28, 75) |
| Volk, R. J. | 1997 | 1333 | USA | Single site | Spanish, Other | Women, Men | high | low | low | low | Range: 39-47 |
| Voluse, A. C. | 2012 | 153 | USA | Single site | English | Women, Men | high | low | high | low | Outpatient drug abusers Mean: 30.66 (9.34)  Residential drug abusers Mean: 41.51 (9.59)  Alcohol abusers  Mean: 43.21 (10.33) |
| Walther L | 2015 | 115 | Sweden | Multi-site | English | Women, Men | low | low | high | low | Mean: 55.6 |
| Werle E | 1997 | 101 | Germany | Single site | English | Women, Men | high | low | low | low | Range: 18-90 |
| Werner M | 1996 | 184 | USA | Single site | English | Women, Men | high | low | high | low | Mean: 17.9 (0.5) Range: 16-20 |
| Werner, M. J | 1994 | 248 | USA | Single site | English | Women | high | low | low | low | Mean: 17.9 (0.5) Range: 16-20 |
| West, Steven | 2001 | 150 | USA | Single site | English | Women, Men | low | low | low | low | Mean: 19 Range: 18-25 |
| Wetterling, T | 1998 | 204 | USA | Single site | English | Women, Men | unclear | low | low | low | Male Mean: 43.1 (15.1)  Female Mean: 43.7 (15.1) |
| Wu, S. I. | 2008 | 404 | Country is not listed | Single site | Chinese, Other | Women, Men | high | low | low | low | Mean: 42.9 (13.6) |
| Wurst F | 2010 | 57 | Austria, Germany, Switzerland | Multi-site | English | Women, Men | high | low | high | high | Mean: 43.56 Range: 24-66 |
| Wurst, Friedrich M.; | 2013 | 456 | Austria | Single site | English | Women, Men | high | low | low | low | Mean: 42.02 (10.5) |
| Yeastedt, J. | 1998 | 46 | USA | Single site | English | Women | high | low | high | low | Cases Mean: 33 Range: 21-50  Controls Mean: 26 Range: 18-62 |
| Yersin, B | 1989 | 301 | Switzerland | Multi-site | French | Women, Men | low | low | low | high | Range: 20-75 |
| Young C | 2010 | 318 | South Africa | Single site | Unclear | Women, Men | low | high |  | low | Mean: 19.5 (1.33) Range: 17-24 |
| Zanis D | 1997 | 62 | USA | Single site | English | Women, Men | low | unclear |  | high | Mean: 36.8 (6.95) |
| Zanis D | 1994 | 98 | USA | Multi-site | English | Men | low | unclear |  | high | Mean: 39 |
| Zavar A | 2015 | 140 | Iran | Multi-site | Other | Women, Men | unclear | unclear | unclear | low | Cases Mean: 32.7 (9.1)  Controls Mean: 30.1 (7.2) |
| Zierau F | 2005 | 130 | Denmark | Single site | Other | Women, Men | low | unclear | unclear | low | Median: 50 Range: 18-82 |

| **Supplementary Table 2. References of Studies Meta-Analyzed, by Scale** | |
| --- | --- |
| Scale | Citation |
| 2  %CDT | Anttila 2003. A new modified gamma-%CDT method improves the detection of problem drinking: studies in alcoholics with or without liver disease. Clin Chim Acta. 338-1-2:45-51 Anttila 2005. Biomarkers of alcohol consumption in patients classified according to the degree of liver disease severity. Scand J Clin Lab Invest. 65-2:141-51 Aradottir 2006. PHosphatidylethanol (PEth) concentrations in blood are correlated to reported alcohol intake in alcohol-dependent patients. Alcohol. 41-4:431-7 Brathen 2001. Detection of alcohol abuse in neurological patients: variables of clinical relevance to the accuracy of the %CDT-TIA and CDTect methods. Alcohol Clin Exp Res. 25-1:46-53 Chrostek 2006. The diagnostic accuracy of carbohydrate-deficient transferrin, sialic acid and commonly used markers of alcohol abuse during abstinence. Clin Chim Acta. 364-1-2:167-71 Fagan 2014. Diagnostic sensitivity of carbohydrate deficient transferrin in heavy drinkers. BMC Gastroenterol. 14-:97 Fleming 2004. Carbohydrate-deficient transferrin: validity of a new alcohol biomarker in a sample of patients with diabetes and hypertension. J Am Board Fam Pract. 17-4:247-55 Forsberg 2002. Screening of binge drinking among patients on an emergency surgical ward. Alcohol. 27-2:77-82 Gomez 2001. Diagnostic usefulness of carbohydrate-deficient transferrin for detecting alcohol-related problems in hospitalized patients. Alcohol. 36-3:266-70 Gough 2015. The Utility of Commonly Used Laboratory Tests to Screen for Excessive Alcohol Use in Clinical Practice. Alcoholism: Clinical and Experimental Research. 39-8:1493-1500 Hastedt 2013. Detecting alcohol abuse: traditional blood alcohol markers compared to ethyl glucuronide (EtG) and fatty acid ethyl esters (FAEEs) measurement in hair. Forensic Sci Med Pathol. 9-4:471-7 Ireland 2011. Operating characteristics of carbohydrate-deficient transferrin (CDT) for identifying unhealthy alcohol use in adults with HIV infection. AIDS Care. 23-11:1483-91 Kechagias 2015. Phosphatidylethanol Compared with Other Blood Tests as a Biomarker of Moderate Alcohol Consumption in Healthy Volunteers: A Prospective Randomized Study. Alcohol. 50-4:399-406 Lesch 1996. Carbohydrate-deficient transferrin as a screening marker for drinking in a general hospital population. Alcohol. 31-3:249-56 Levola 2015. Screening for atâ€_risk drinking in a population reporting symptoms of depression: A validation of the AUDIT, AUDIT-C, and AUDIT-3. Alcoholism: Clinical and Experimental Research. 39-7:1186-1192 Madhubala 2013. Serum carbohydrate deficient transferrin as a sensitive marker in diagnosing alcohol abuse: A case - Control study. Journal of Clinical and Diagnostic Research. 7-2:197-200 McDonald 2013. Comparative performance of biomarkers of alcohol consumption in a population sample of working-aged men in Russia: the Izhevsk Family Study. Addiction. 108-9:1579-89 Mikkelsen 1998. Carbohydrate-deficient transferrin: marker of actual alcohol consumption or chronic alcohol misuse?. Alcohol Alcohol. 33-6:646-50 Morini 2011. Chronic excessive alcohol consumption diagnosis: comparison between traditional biomarkers and ethyl glucuronide in hair, a study on a real population. 33-5:654-7 Mundle 1999. Biological markers as indicators for relapse in alcohol-dependent patients. Addiction Biology. 4-2:209-214 Mundle 2000. Sex differences of carbohydrate-deficient transferrin, gamma-glutamyltransferase, and mean corpuscular volume in alcohol-dependent patients. Alcohol Clin Exp Res. 24-9:1400-5 Neumann 2008. Value of ethyl glucuronide in plasma as a biomarker for recent alcohol consumption in the emergency room. Alcohol Alcohol. 43-4:431-5 Neumann 2009. Screening trauma patients with the alcohol use disorders identification test and biomarkers of alcohol use. Alcohol Clin Exp Res. 33-6:970-6 Radosavljevic 1995. Elevated levels of serum carbohydrate deficient transferrin are not specific for alcohol abuse in patients with liver disease. J Hepatol. 23-6:706-11 Rinck 2007. Combinations of carbohydrate-deficient transferrin, mean corpuscular erythrocyte volume, gamma-glutamyltransferase, homocysteine and folate increase the significance of biological markers in alcohol dependent patients. 89-1:60-5 Song 2014. Determination of carbohydrate-deficient transferrin in a Han Chinese population. BMC Biochem. 15-:5 Staufer 2011. Urinary ethyl glucuronide as a novel screening tool in patients pre- and post-liver transplantation improves detection of alcohol consumption. Hepatology. 54-5:1640-9 Taracha 2002. The activity of beta-hexosaminidase (uHex) and gamma-glutamyltransferase (uGGT) in urine as non-invasive markers of chronic alcohol abuse: II. Opiate-dependent subjects receiving methadone substitution. World J Biol Psychiatry. 3-1:44-9 Thiesen 2010. Biological markers of problem drinking in homeless patients. 35-3:260-2 Walther 2015. Phosphatidylethanol is Superior to Carbohydrate-Deficient Transferrin and Î³-Glutamyltransferase as an Alcohol Marker and is a Reliable Estimate of Alcohol Consumption Level. Alcoholism: Clinical and Experimental Research. 39-11:2200-2208 |
| 59  ADS | Chantarujikapong 1997. Comparison of the Alcohol Dependence Scale and diagnostic interview schedule in homeless women. Alcohol Clin Exp Res. 21-4:586-95 Doyle 2009. A validation study of the Alcohol Dependence Scale. Journal of Studies on Alcohol and Drugs. 70-5:689-699 Drake 1990. Diagnosis of Alcohol Use Disorders in Schizophrenia. Schizophrenia Bulletin. 16-1:57-67 |
| 69  ALT | Arumalla 2012. Sensitivity, specificity and diagnostic efficiency of serum sialic acid as a biochemical marker in alcohol abuse. British Journal of Medical Practitioners. 5-2: Bell 1993. Serum carbohydrate-deficient transferrin as a marker of alcohol consumption in patients with chronic liver diseases. Alcohol Clin Exp Res. 17-2:246-52 Bell 1994. Carbohydrate-deficient transferrin and other markers of high alcohol consumption: a study of 502 patients admitted consecutively to a medical department. Alcohol Clin Exp Res. 18-5:1103-8 Chrostek 2006. The diagnostic accuracy of carbohydrate-deficient transferrin, sialic acid and commonly used markers of alcohol abuse during abstinence. Clin Chim Acta. 364-1-2:167-71 Gomez 2001. Diagnostic usefulness of carbohydrate-deficient transferrin for detecting alcohol-related problems in hospitalized patients. Alcohol Alcohol. 36-3:266-70 Gough 2015. The Utility of Commonly Used Laboratory Tests to Screen for Excessive Alcohol Use in Clinical Practice. Alcoholism: Clinical and Experimental Research. 39-8:1493-1500 GÃ³mez 2006. The diagnostic usefulness of AUDIT and AUDIT-C for detecting hazardous drinkers in the elderly. Aging & Mental Health. 10-5:558-561 Hannuksela 1992. Evaluation of plasma cholesteryl ester transfer protein (CETP) activity as a marker of alcoholism. Alcohol Alcohol. 27-5:557-62 Hastedt 2013. Detecting alcohol abuse: traditional blood alcohol markers compared to ethyl glucuronide (EtG) and fatty acid ethyl esters (FAEEs) measurement in hair. Forensic Sci Med Pathol. 9-4:471-7 Hazelett 1998. Evaluation of acetaldehyde-modified hemoglobin and other markers of chronic heavy alcohol use: effects of gender and hemoglobin concentration. Alcohol Clin Exp Res. 22-8:1813-9 Kwoh-Gain 1990. Desialylated transferrin and mitochondrial aspartate aminotransferase compared as laboratory markers of excessive alcohol consumption. Clin Chem. 36-6:841-5 Madhubala 2013. Serum carbohydrate deficient transferrin as a sensitive marker in diagnosing alcohol abuse: A case - Control study. Journal of Clinical and Diagnostic Research. 7-2:197-200 McDonald 2013. Comparative performance of biomarkers of alcohol consumption in a population sample of working-aged men in Russia: the Izhevsk Family Study. Addiction. 108-9:1579-89 Morini 2011. Chronic excessive alcohol consumption diagnosis: comparison between traditional biomarkers and ethyl glucuronide in hair, a study on a real population. 33-5:654-7 Pirro 2011. Chemometric evaluation of nine alcohol biomarkers in a large population of clinically-classified subjects: pre-eminence of ethyl glucuronide concentration in hair for confirmatory classification. Anal Bioanal Chem. 401-7:2153-64 Reid 2005. Are Commonly Ordered Lab Tests Useful Screens for Alcohol Disorders in Older Male Veterans Receiving Primary Care?. Substance Abuse. 26-2:25-32 Reynaud 1998. Usefulness of carbohydrate-deficient transferrin in alcoholic patients with normal gamma-glutamyltranspeptidase. Alcohol Clin Exp Res. 22-3:615-8 Rublo 1997. Carbohydrate-deficient transferrin as a marker of alcohol consumption in male patients with liver disease. Alcohol Clin Exp Res. 21-5:923-7 Sillanaukee 1998. Carbohydrate-deficient transferrin and conventional alcohol markers as indicators for brief intervention among heavy drinkers in primary health care. Alcohol Clin Exp Res. 22-4:892-6 Sillanaukee 1999. Sialic acid: new potential marker of alcohol abuse. Alcohol Clin Exp Res. 23-6:1039-43 Spies 1995. Relevance of carbohydrate-deficient transferrin as a predictor of alcoholism in intensive care patients following trauma. J Trauma. 39-4:742-8 Staufer 2011. Urinary ethyl glucuronide as a novel screening tool in patients pre- and post-liver transplantation improves detection of alcohol consumption. Hepatology. 54-5:1640-9 Stowell 1997. Comparison of two commercial test kits for quantification of serum carbohydrate-deficient transferrin. Alcohol Alcohol. 32-4:507-16 Thiesen 2010. Biological markers of problem drinking in homeless patients. 35-3:260-2 Wetterling 1998. Comparison of cage and mast with the alcohol markers CDT, gamma-GT, ALAT, ASAT and MCV. Alcohol Alcohol. 33-4:424-30 |
| 79  ASI | Alterman 1998. New scales to assess change in the addiction severity index for the opioid, cocaine, and alcohol dependent. Psychology of Addictive Behaviors. 12-4:233-246 Chermack 2000. Comparison of patient self-reports and urinalysis results obtained under naturalistic methadone treatment conditions. 59-1:43-9 DemÄ°rbaÅŸ 2014. Reliability and validity of the Turkish version of the addiction severity index in male alcohol dependents. Noropsikiyatri Arsivi. 51-3:216-221 Hendriks 1989. The Addiction Severity Index: Reliability and validity in a Dutch addict population. Journal of Substance Abuse Treatment. 6-2:133-141 |
| 83  ASI-A | Alterman 1998. New scales to assess change in the addiction severity index for the opioid, cocaine, and alcohol dependent. Psychology of Addictive Behaviors. 12-4:233-246 Amoureus 1994. The Addiction Severity Index in penitentiaries. International Journal of Offender Therapy and Comparative Criminology. 38-4:309-318 Appleby 1997. Assessing substance use in multiproblem patients: reliability and validity of the Addiction Severity Index in a mental hospital population. J Nerv Ment Dis. 185-3:159-65 Brochu 1995. An Addiction Severity Index for inmates. International Medical Journal. 2-1:54-58 Carey 1997. Reliability and validity of the addiction severity index among outpatients with severe mental illness. Psychological Assessment. 9-4:422-428 Currie 2004. Factor validation of the addiction severity index scale structure in persons with concurrent disorders. 16-3:326-9 DeJong 1995. The Addiction Severity Index: Reliability and validity in a Dutch alcoholic population. International Journal of the Addictions. 30-5:605-616 DemÄ°rbaÅŸ 2014. Reliability and validity of the Turkish version of the addiction severity index in male alcohol dependents. Noropsikiyatri Arsivi. 51-3:216-221 Doub 2001. Psychometric properties of the Addiction Severity Index in clients with co-occurring substance-related and mental health disorders. -:1074 Haller 2010. Hair analysis versus conventional methods of drug testing in substance abusers seeking organ transplantation. Am J Transplant. 10-5:1305-11 Hendriks 1989. The Addiction Severity Index: Reliability and validity in a Dutch addict population. Journal of Substance Abuse Treatment. 6-2:133-141 Krenz 2004. French Version of the Addiction Severity Index (5th Edition): Validity and Reliability among Swiss Opiate-Dependent Patients. European Addiction Research. 10-4:173-179 Rikoon 2006. Predicting DSM-IV dependence diagnoses from Addiction Severity Index composite scores. 31-1:17-24 Zanis 1994. Reliability and validity of the Addiction Severity Index with a homeless sample. 11-6:541-8 Zanis 1997. Is the Addiction Severity Index a reliable and valid assessment instrument among clients with severe and persistent mental illness and substance abuse disorders?. Community Ment Health J. 33-3:213-27 |
| 84  ASI-D | Alterman 1998. New scales to assess change in the addiction severity index for the opioid, cocaine, and alcohol dependent. Psychology of Addictive Behaviors. 12-4:233-246 Amoureus 1994. The Addiction Severity Index in penitentiaries. International Journal of Offender Therapy and Comparative Criminology. 38-4:309-318 Appleby 1997. Assessing substance use in multiproblem patients: reliability and validity of the Addiction Severity Index in a mental hospital population. J Nerv Ment Dis. 185-3:159-65 Brochu 1995. An Addiction Severity Index for inmates. International Medical Journal. 2-1:54-58 Carey 1997. Reliability and validity of the addiction severity index among outpatients with severe mental illness. Psychological Assessment. 9-4:422-428 Currie 2004. Factor validation of the addiction severity index scale structure in persons with concurrent disorders. 16-3:326-9 Doub 2001. Psychometric properties of the Addiction Severity Index in clients with co-occurring substance-related and mental health disorders. -:1074 Haller 2010. Hair analysis versus conventional methods of drug testing in substance abusers seeking organ transplantation. Am J Transplant. 10-5:1305-11 Hendriks 1989. The Addiction Severity Index: Reliability and validity in a Dutch addict population. Journal of Substance Abuse Treatment. 6-2:133-141 Krenz 2004. French Version of the Addiction Severity Index (5th Edition): Validity and Reliability among Swiss Opiate-Dependent Patients. European Addiction Research. 10-4:173-179 Rikoon 2006. Predicting DSM-IV dependence diagnoses from Addiction Severity Index composite scores. 31-1:17-24 Zanis 1994. Reliability and validity of the Addiction Severity Index with a homeless sample. 11-6:541-8 Zanis 1997. Is the Addiction Severity Index a reliable and valid assessment instrument among clients with severe and persistent mental illness and substance abuse disorders?. Community Ment Health J. 33-3:213-27 |
| 85  ASSIST | Hides 2009. The reliability and validity of the Alcohol, Smoking and Substance Involvement Screening Test (ASSIST) in first-episode psychosis. 34-10:821-5 Johnson 2015. Validation of the ASSIST for Detecting Unhealthy Alcohol Use and Alcohol Use Disorders in Urgent Care Patients. Alcoholism: Clinical and Experimental Research. 39-6:1093-1099 Khan 2011. Validation of the French version of the alcohol, smoking and substance involvement screening test (ASSIST). 17-4:190-7 Khan 2012. Validation of the French version of the alcohol, smoking and substance involvement screening test (ASSIST) in the elderly. Subst Abuse Treat Prev Policy. 7-:14 McNeely 2014. Testâ€“retest reliability of a self-administered Alcohol, Smoking and Substance Involvement Screening Test (ASSIST) in primary care patients. Journal of Substance Abuse Treatment. 47-1:93-101 |
| 86  AST | Anttila 2003. A new modified gamma-%CDT method improves the detection of problem drinking: studies in alcoholics with or without liver disease. Clin Chim Acta. 338-1-2:45-51 Anttila 2005. Biomarkers of alcohol consumption in patients classified according to the degree of liver disease severity. Scand J Clin Lab Invest. 65-2:141-51 Arumalla 2012. Sensitivity, specificity and diagnostic efficiency of serum sialic acid as a biochemical marker in alcohol abuse. British Journal of Medical Practitioners. 5-2: Bell 1993. Serum carbohydrate-deficient transferrin as a marker of alcohol consumption in patients with chronic liver diseases. Alcohol Clin Exp Res. 17-2:246-52 Bell 1994. Carbohydrate-deficient transferrin and other markers of high alcohol consumption: a study of 502 patients admitted consecutively to a medical department. Alcohol Clin Exp Res. 18-5:1103-8 Chrostek 2006. The diagnostic accuracy of carbohydrate-deficient transferrin, sialic acid and commonly used markers of alcohol abuse during abstinence. Clin Chim Acta. 364-1-2:167-71 Conigrave 2002. CDT, GGT, and AST as markers of alcohol use: the WHO/ISBRA collaborative project. Alcohol Clin Exp Res. 26-3:332-9 Coulton 2006. Opportunistic screening for alcohol use disorders in primary care: comparative study. Bmj. 332-7540:511-7 Gomez 2001. Diagnostic usefulness of carbohydrate-deficient transferrin for detecting alcohol-related problems in hospitalized patients. Alcohol Alcohol. 36-3:266-70 Gough 2015. The Utility of Commonly Used Laboratory Tests to Screen for Excessive Alcohol Use in Clinical Practice. Alcoholism: Clinical and Experimental Research. 39-8:1493-1500 Gronbaek 1995. Carbohydrate-deficient transferrin--a valid marker of alcoholism in population studies? Results from the Copenhagen City Heart Study. Alcohol Clin Exp Res. 19-2:457-61 GÃ³mez 2006. The diagnostic usefulness of AUDIT and AUDIT-C for detecting hazardous drinkers in the elderly. Aging & Mental Health. 10-5:558-561 Hannuksela 1992. Evaluation of plasma cholesteryl ester transfer protein (CETP) activity as a marker of alcoholism. Alcohol Alcohol. 27-5:557-62 Hastedt 2013. Detecting alcohol abuse: traditional blood alcohol markers compared to ethyl glucuronide (EtG) and fatty acid ethyl esters (FAEEs) measurement in hair. Forensic Sci Med Pathol. 9-4:471-7 Hazelett 1998. Evaluation of acetaldehyde-modified hemoglobin and other markers of chronic heavy alcohol use: effects of gender and hemoglobin concentration. Alcohol Clin Exp Res. 22-8:1813-9 Korzec 2009. Validation of the Bayesian Alcoholism Test compared to single biomarkers in detecting harmful drinking. Alcohol and Alcoholism. 44-4:398-402 Kwoh-Gain 1990. Desialylated transferrin and mitochondrial aspartate aminotransferase compared as laboratory markers of excessive alcohol consumption. Clin Chem. 36-6:841-5 Madhubala 2013. Serum carbohydrate deficient transferrin as a sensitive marker in diagnosing alcohol abuse: A case - Control study. Journal of Clinical and Diagnostic Research. 7-2:197-200 McDonald 2013. Comparative performance of biomarkers of alcohol consumption in a population sample of working-aged men in Russia: the Izhevsk Family Study. Addiction. 108-9:1579-89 Morini 2011. Chronic excessive alcohol consumption diagnosis: comparison between traditional biomarkers and ethyl glucuronide in hair, a study on a real population. 33-5:654-7 Pirro 2011. Chemometric evaluation of nine alcohol biomarkers in a large population of clinically-classified subjects: pre-eminence of ethyl glucuronide concentration in hair for confirmatory classification. Anal Bioanal Chem. 401-7:2153-64 Reid 2005. Are Commonly Ordered Lab Tests Useful Screens for Alcohol Disorders in Older Male Veterans Receiving Primary Care?. Substance Abuse. 26-2:25-32 Reynaud 1998. Usefulness of carbohydrate-deficient transferrin in alcoholic patients with normal gamma-glutamyltranspeptidase. Alcohol Clin Exp Res. 22-3:615-8 Rublo 1997. Carbohydrate-deficient transferrin as a marker of alcohol consumption in male patients with liver disease. Alcohol Clin Exp Res. 21-5:923-7 Ryb 1999. Use of blood alcohol concentration and laboratory tests to detect current alcohol dependence in trauma center patients. J Trauma. 47-5:874-9; discussion 879-80 Savola 2004. Blood alcohol is the best indicator of hazardous alcohol drinking in young adults and working-age patients with trauma. Alcohol Alcohol. 39-4:340-5 Sillanaukee 1998. Carbohydrate-deficient transferrin and conventional alcohol markers as indicators for brief intervention among heavy drinkers in primary health care. Alcohol Clin Exp Res. 22-4:892-6 Sillanaukee 1999. Sialic acid: new potential marker of alcohol abuse. Alcohol Clin Exp Res. 23-6:1039-43 Spies 1995. Relevance of carbohydrate-deficient transferrin as a predictor of alcoholism in intensive care patients following trauma. J Trauma. 39-4:742-8 Staufer 2011. Urinary ethyl glucuronide as a novel screening tool in patients pre- and post-liver transplantation improves detection of alcohol consumption. Hepatology. 54-5:1640-9 Stowell 1997. Comparison of two commercial test kits for quantification of serum carbohydrate-deficient transferrin. Alcohol Alcohol. 32-4:507-16 Wetterling 1998. Comparison of cage and mast with the alcohol markers CDT, gamma-GT, ALAT, ASAT and MCV. Alcohol Alcohol. 33-4:424-30 Zierau 2005. Validation of a self-administered modified CAGE test (CAGE-C) in a somatic hospital ward: comparison with biochemical markers. Scand J Clin Lab Invest. 65-7:615-22 |
| 89  AST/ALT | Kwoh-Gain 1990. Desialylated transferrin and mitochondrial aspartate aminotransferase compared as laboratory markers of excessive alcohol consumption. Clin Chem. 36-6:841-5 Reid 2005. Are Commonly Ordered Lab Tests Useful Screens for Alcohol Disorders in Older Male Veterans Receiving Primary Care?. Substance Abuse. 26-2:25-32 Sharpe 1996. Biochemical markers of alcohol abuse. Qjm. 89-2:137-44 |
| 94  AUDIT | Aalto 2006. Effectiveness of structured questionnaires for screening heavy drinking in middle-aged women. Alcohol Clin Exp Res. 30-11:1884-8 Aalto 2009. AUDIT and its abbreviated versions in detecting heavy and binge drinking in a general population survey. 103-1-2:25-9 Aalto 2011. The Alcohol Use Disorders Identification Test (AUDIT) and its derivatives in screening for heavy drinking among the elderly. 26-9:881-5 Adewuya 2005. Validation of the Alcohol Use Disorders Identification Test (AUDIT) as a screening tool for alcohol-related problems among Nigerian university students. Alcohol and Alcoholism. 40-6:575-577 Agabio 2007. Alcohol use disorders, and at-risk drinking in patients affected by a mood disorder, in Cagliari, Italy: sensitivity and specificity of different questionnaires. Alcohol Alcohol. 42-6:575-81 Berg 2011. Diagnosis after an acute psychiatric inpatient stay: How do psychotic and non-psychotic diagnoses relate to the results of psychometric tests of substance abuse?. Journal of Psychiatric Intensive Care. 7-1:11-16 Bergman 2002. Alcohol use among Swedes and a psychometric evaluation of the Alcohol Use Disorders Identification Test. Alcohol and Alcoholism. 37-3:245-251 Bischof 2007. Development and evaluation of a screening instrument for alcohol-use disorders and at-risk drinking: the brief alcohol screening instrument for medical care (BASIC). 68-4:607-14 Boschloo 2010. The performance of the Alcohol Use Disorder Identification Test (AUDIT) in detecting alcohol abuse and dependence in a population of depressed or anxious persons. 126-3:441-6 Bradley 1998. Screening for problem drinking: comparison of CAGE and AUDIT. Ambulatory Care Quality Improvement Project (ACQUIP). Alcohol Use Disorders Identification Test. 13-6:379-88 Bradley 2003. Two brief alcohol-screening tests From the Alcohol Use Disorders Identification Test (AUDIT): validation in a female Veterans Affairs patient population. Arch Intern Med. 163-7:821-9 Bryce 2015. Screening for Substance Use Disorders Following Traumatic Brain Injury: Examining the Validity of the AUDIT and the DAST. Journal of Head Trauma Rehabilitation. 30-5:E40-E48 Bush 1998. The AUDIT alcohol consumption questions (AUDIT-C): an effective brief screening test for problem drinking. Ambulatory Care Quality Improvement Project (ACQUIP). Alcohol Use Disorders Identification Test. Arch Intern Med. 158-16:1789-95 Carey 2003. Psychometric evaluation of the alcohol use disorders identification test and short drug abuse screening test with psychiatric patients in India. J Clin Psychiatry. 64-7:767-74 Cassidy 2008. Validation of the alcohol use disorders identification test and the drug abuse screening test in first episode psychosis. Can J Psychiatry. 53-1:26-33 Chang 2006. Identifying Risk Drinking in Expectant Fathers. Birth: Issues in Perinatal Care. 33-2:110-116 Chen 2004. Prevalence and identification of alcohol use disorders among nonpsychiatric inpatients in one general hospital. 26-3:219-25 Cherpitel 1995. Ethnic differences in performance of screening instruments for identifying harmful drinking and alcohol dependence in the emergency room. Alcohol Clin Exp Res. 19-3:628-34 Cherpitel 1997. Comparison of screening instruments for alcohol problems between black and white emergency room patients from two regions of the country. Alcohol Clin Exp Res. 21-8:1391-7 Cherpitel 2000. Screening instruments for alcohol problems: a comparison of cut points between Mexican American and Mexican patients in the emergency room. Subst Use Misuse. 35-10:1419-30 Cherpitel 2001. Screening for alcohol problems: A comparison of instrument performance among black emergency department and primary care patients. Journal of Substance Use. 5-4:290-297 Clements 1998. A critical evaluation of several alcohol screening instruments using the CIDI-SAM as a criterion measure. Alcohol Clin Exp Res. 22-5:985-93 Conley 2001. Construct validity of the MAST and AUDIT with multiple offender drunk drivers. Journal of Substance Abuse Treatment. 20-4:287-295 Conley 2006. Court Ordered Multiple Offender Drunk Drivers: Validity and Reliability of Rapid Assessment. Journal of Social Work Practice in the Addictions. 6-3:37-51 Cook 2005. Alcohol screening in young persons attending a sexually transmitted disease clinic. Comparison of AUDIT, CRAFFT, and CAGE instruments. 20-1:1-6 CortÃ©s-TomÃ¡s 2016. Different versions of the Alcohol Use Disorders Identification Test (AUDIT) as screening instruments for underage binge drinking. Drug and Alcohol Dependence. 158-:52-59 Coulton 2006. Opportunistic screening for alcohol use disorders in primary care: comparative study. Bmj. 332-7540:511-7 Crawford 2013. Diagnostic efficiency of the AUDIT-C in U.S. veterans with military service since September 11, 2001. 132-1-2:101-6 Cremonte 2008. Performance of screening instruments for alcohol use disorders in emergency departments patients in Argentina. Substance Use & Misuse. 43-1:125-138 Cremonte 2010. Psychometric properties of alcohol screening tests in the emergency department in Argentina, Mexico and the United States. 35-9:818-25 Dawe 2000. An examination of the utility of the AUDIT in people with schizophrenia. J Stud Alcohol. 61-5:744-50 DeMartini 2012. Optimizing the use of the AUDIT for alcohol screening in college students. Psychological Assessment. 24-4:954-963 Dewost 2006. Fast alcohol consumption evaluation: a screening instrument adapted for French general practitioners. Alcohol Clin Exp Res. 30-11:1889-95 Dolman 2005. Combining the audit questionnaire and biochemical markers to assess alcohol use and risk of alcohol withdrawal in medical inpatients. Alcohol Alcohol. 40-6:515-9 Durbeej 2010. Validation of the Alcohol Use Disorders Identification Test and the Drug Use Disorders Identification Test in a Swedish sample of suspected offenders with signs of mental health problems: Results from the Mental Disorder, Substance Abuse and Crime study. Journal of Substance Abuse Treatment. 39-4:364-377 Fillmore 2011. Defining "binge" drinking as five drinks per occasion or drinking to a .08% BAC: which is more sensitive to risk?. Am J Addict. 20-5:468-75 Fleming 1991. The alcohol use disorders identification test (AUDIT) in a college sample. Int J Addict. 26-11:1173-85 Fulbrook 2015. Validity of the Paddington Alcohol Test in an Australian emergency department. Alcohol and Alcoholism. 50-4:407-412 Gache 2005. The Alcohol Use Disorders Identification Test (AUDIT) as a screening tool for excessive drinking in primary care: reliability and validity of a French version. Alcohol Clin Exp Res. 29-11:2001-7 Gammeter 2006. Frequency of alcohol use disorders in patients admitted in a psychiatric hospital according to admission diagnosis. Schweizer Archiv fur Neurologie und Psychiatrie. 157-6:290-296 Gates 2007. Alcohol screening instruments and psychiatric evaluation outcomes in military aviation personnel. Aviat Space Environ Med. 78-1:48-51 Geneste 2012. CAGE, RAPS4, RAPS4-QF and AUDIT screening tests for men and women admitted for acute alcohol intoxication to an emergency department: are standard thresholds appropriate?. Alcohol Alcohol. 47-3:273-81 Giang 2005. The use of audit to assess level of alcohol problems in rural Vietnam. Alcohol Alcohol. 40-6:578-83 Gordon 2001. Three questions can detect hazardous drinkers. The Journal of Family Practice. 50-4:313-320 Gual 2002. Audit-3 and audit-4: effectiveness of two short forms of the alcohol use disorders identification test. Alcohol Alcohol. 37-6:591-6 Gundersen 2013. Evaluation of the Alcohol Use Disorders Identification Test and the Drug Use Disorders Identification Test among patients at a Norwegian psychiatric emergency ward. European Addiction Research. 19-5:252-260 Guo 2008. An epidemiological survey of alcohol use disorders in a Tibetan population. 159-1-2:56-66 Gureje 1992. Alcohol abuse and dependence in an urban primary care clinic in Nigeria. 30-2:163-7 GÃ³mez 2006. The diagnostic usefulness of AUDIT and AUDIT-C for detecting hazardous drinkers in the elderly. Aging & Mental Health. 10-5:558-561 Hallinan 2011. Factor structure and validity of the alcohol use disorders identification test (AUDIT) in a sample of mentally disordered offenders. Journal of Forensic Psychiatry and Psychology. 22-4:586-602 Hays 1995. Response burden, reliability, and validity of the CAGE, Short MAST, and AUDIT alcohol screening measures. Behavior Research Methods, Instruments & Computers. 27-2:277-280 Hearon 2015. Improving the efficiency of drug use disorder screening in psychiatric settings: validation of a single-item screen. Am J Drug Alcohol Abuse. 41-2:173-6 Hildebrand 2015. Exploration of the (Interrater) Reliability and Latent Factor Structure of the Alcohol Use Disorders Identification Test (AUDIT) and the Drug Use Disorders Identification Test (DUDIT) in a Sample of Dutch Probationers. Subst Use Misuse. 50-10:1294-306 Isaacson 1994. Screening with the Alcohol use Disorders Identification Test (AUDIT) in an inner-city population. 9-10:550-3 Johnson 2013. Use of AUDITâ€_based measures to identify unhealthy alcohol use and alcohol dependence in primary care: A validation study. Alcoholism: Clinical and Experimental Research. 37-Suppl 1:E253-E259 Kader 2012. A preliminary investigation of the AUDIT and DUDIT in comparison to biomarkers for alcohol and drug use among HIV-infected clinic attendees in Cape Town, South Africa. Afr J Psychiatry (Johannesbg). 15-5:346-51 Kavanagh 2011. The DrugCheck Problem List: a new screen for substance use disorders in people with psychosis. 36-9:927-32 Kawada 2011. The alcohol use disorders identification test: reliability study of the Japanese version. Alcohol. 45-3:205-7 Kelly 2002. A comparison of alcohol screening instruments among under-aged drinkers treated in emergency departments. Alcohol Alcohol. 37-5:444-50 Kelly 2004. Alcohol use disorders among emergency department-treated older adolescents: a new brief screen (RUFT-Cut) using the AUDIT, CAGE, CRAFFT, and RAPS-QF. Alcohol Clin Exp Res. 28-5:746-53 Kim 2008. Psychometric properties of the alcohol use disorders identification test: A Korean version. Archives of Psychiatric Nursing. 22-4:190-199 Kim 2013. The 5-item Alcohol Use Disorders Identification Test (AUDIT-5): An effective brief screening test for problem drinking, alcohol use disorders and alcohol dependence. Alcohol and Alcoholism. 48-1:68-73 Knibbe 2006. A comparison of the Alcohol Use Disorder Identification Test (AUDIT) in general population surveys in nine European countries. Alcohol and Alcoholism. 41-Suppl1:19-25 Knight 2003. Validity of brief alcohol screening tests among adolescents: A comparison of the AUDIT, POSIT, CAGE, and CRAFFT. Alcoholism: Clinical and Experimental Research. 27-1:67-73 Kokotailo 2004. Validity of the alcohol use disorders identification test in college students. Alcohol Clin Exp Res. 28-6:914-20 Kowalyszyn 2003. Family functioning, alcohol expectancies and alcohol-related problems in a remote Aboriginal Australian community: A preliminary psychometric validation study. Drug and Alcohol Review. 22-1:53-59 Levola 2015. Screening for atâ€_risk drinking in a population reporting symptoms of depression: A validation of the AUDIT, AUDIT-C, and AUDIT-3. Alcoholism: Clinical and Experimental Research. 39-7:1186-1192 Lundin 2015. The Use of Alcohol Use Disorders Identification Test (AUDIT) in Detecting Alcohol Use Disorder and Risk Drinking in the General Population: Validation of AUDIT Using Schedules for Clinical Assessment in Neuropsychiatry. Alcoholism: Clinical and Experimental Research. 39-1:158-165 MacKenzie 1996. Identifying hazardous or harmful alcohol use in medical admissions: a comparison of audit, cage and brief mast. Alcohol Alcohol. 31-6:591-9 Maisto 2000. Use of the AUDIT and the DAST-10 to identify alcohol and drug use disorders among adults with a severe and persistent mental illness. 12-2:186-92 Matano 2003. Assessment of binge drinking of alcohol in highly educated employees. 28-7:1299-310 Matthews 2010. Potential risk of patient misclassification using a point-of-care testing kit for urine drugs of abuse. Br J Biomed Sci. 67-4:218-20 McCambridge 2009. Short forms of the AUDIT in a Web-based study of young drinkers. Drug and Alcohol Review. 28-1:18-24 McCann 2000. Reliability and validity of screening instruments for drug and alcohol abuse in adults seeking evaluation for attention-deficit/hyperactivity disorder. Am J Addict. 9-1:1-9 McQuade 2000. Detecting symptoms of alcohol abuse in primary care settings. Arch Fam Med. 9-9:814-21 Meneses-Gaya 2010. Is the full version of the AUDIT really necessary? Study of the validity and internal construct of its abbreviated versions. Alcohol Clin Exp Res. 34-8:1417-24 Merikallio-Pajunen 2004. Comparison of new alcohol use test, the Helsinki Alcohol Use Test questionnaire, and Alcohol Use Disorders Identification Test and laboratory markers serum gamma-glutamyl transferase and carbohydrate-deficient transferrin. 29-4:717-22 Morojele 2015. Reliability and factor structure of the audit among male and female bar patrons in a rural area of South Africa. African Journal of Drug and Alcohol Studies. 14-1:23-35 Morton 1996. Performance of alcoholism screening questionnaires in elderly veterans. Am J Med. 101-2:153-9 Moussas 2009. The Alcohol Use Disorders Identification Test (AUDIT): Reliability and validity of the Greek version. Annals of General Psychiatry. 8-: Myer 2008. Common mental disorders among HIV-infected individuals in South Africa: prevalence, predictors, and validation of brief psychiatric rating scales. AIDS Patient Care STDS. 22-2:147-58 Nesvag 2010. The use of screening instruments for detecting alcohol and other drug use disorders in first-episode psychosis. 177-1-2:228-34 Neumann 2004. Gender Differences in the Performance of a Computerized Version of the Alcohol Use Disorders Identification Test in Subcritically Injured Patients Who Are Admitted to the Emergency Department. Alcoholism: Clinical and Experimental Research. 28-11:1693-1701 Neumann 2012. Does the Alcohol Use Disorders Identification Test â€“ Consumption identify the same patient population as the full 10-item Alcohol Use Disorders Identification Test?. Journal of Substance Abuse Treatment. 43-1:80-85 O'Hare 1999. Validating the alcohol use disorder identification test with college first-offenders. Journal of Substance Abuse Treatment. 17-1-2:113-119 O'Hare 2010. Validating the Alcohol Use Disorders Identification Test with persons who have a serious mental illness. -:299-66 Pal 2004. Validation of the Alcohol Use Disorders Identification Test (AUDIT) in urban community outreach and de-addiction center samples in North India. Journal of Studies on Alcohol. 65-6:794-800 Philpot 2003. Screening for problem drinking in older people referred to a mental health service: a comparison of CAGE and AUDIT. Aging Ment Health. 7-3:171-5 Piccinelli 1997. Efficacy of the alcohol use disorders identification test as a screening tool for hazardous alcohol intake and related disorders in primary care: a validity study. BMJ. 314-7078:420-4 Plackett 2015. Screening for at-risk drinking behavior in trauma patients. J Am Osteopath Assoc. 115-6:376-82 Pradhan 2012. The alcohol use disorders identification test (AUDIT): validation of a Nepali version for the detection of alcohol use disorders and hazardous drinking in medical settings. Subst Abuse Treat Prev Policy. 7-:42 Richoux 2011. Alcohol use disorders in the emergency ward: Choice of the best mode of assessment and identification of at-risk situations. International Journal of Emergency Medicine. 4-1: Rumpf 2002. Screening for alcohol use disorders and at-risk drinking in the general population: psychometric performance of three questionnaires. Alcohol Alcohol. 37-3:261-8 Rumpf 2013. Screening questionnaires for problem drinking in adolescents: performance of AUDIT, AUDIT-C, CRAFFT and POSIT. 19-3:121-7 Ryou 2012. Usefulness of Alcohol-screening Instruments in Detecting Problem Drinking among Elderly Male Drinkers. Korean J Fam Med. 33-3:126-33 Saitz 1999. Alcohol abuse and dependence in Latinos living in the United States: validation of the CAGE (4M) questions. Arch Intern Med. 159-7:718-24 Santis 2009. The Alcohol Use Disorders Identification Test (AUDIT) as a screening instrument for adolescents. 103-3:155-8 Seale 2006. Primary Care Validation of a Single Screening Question for Drinkers. Journal of Studies on Alcohol. 67-5:778-784 Searle 2015. The validity of military screening for mental health problems: Diagnostic accuracy of the PCL, K10 and AUDIT scales in an entire military population. International Journal of Methods in Psychiatric Research. 24-1:32-45 Selin 2003. Test-Retest Reliability of the Alcohol Use Disorder Identification Test in a General Population Sample. Alcoholism: Clinical and Experimental Research. 27-9:1428-1435 Seppa 1995. Effectiveness of the Alcohol Use Disorders Identification Test in occupational health screenings. Alcohol Clin Exp Res. 19-4:999-1003 Sinadinovic 2010. Internet-based assessment and self-monitoring of problematic alcohol and drug use. 35-5:464-70 Sinadinovic 2011. Population screening of risky alcohol and drug use via Internet and Interactive Voice response (IVR): A feasibility and psychometric study in a random sample. Drug and Alcohol Dependence. 114-1:55-60 Skipsey 1997. Utility of the AUDIT for identification of hazardous or harmful drinking in drug-dependent patients. Drug Alcohol Depend. 45-3:157-63 Soderstrom 1997. The accuracy of the CAGE, the Brief Michigan Alcoholism Screening Test, and the Alcohol Use Disorders Identification Test in screening trauma center patients for alcoholism. J Trauma. 43-6:962-9 Soderstrom 1998. Screening trauma patients for alcoholism according to NIAAA guidelines with alcohol use disorders identification test questions. Alcohol Clin Exp Res. 22-7:1470-5 Steinbauer 1998. Ethnic and sex bias in primary care screening tests for alcohol use disorders. Ann Intern Med. 129-5:353-62 Thomas 2014. Predictive validity of the AUDIT for hazardous alcohol consumption in recently released prisoners. Drug and Alcohol Dependence. 134-:322-329 Tsai 2005. Alcohol Use Disorders Identification Test (AUDIT): Establishment of Cut-off Scores in a Hospitalized Chinese Population. Alcoholism: Clinical and Experimental Research. 29-1:53-57 Vitesnikova 2014. Use of AUDIT-C as a tool to identify hazardous alcohol consumption in admitted trauma patients. Injury. 45-9:1440-4 Volk 1997. Item bias in the CAGE screening test for alcohol use disorders. 12-12:763-9 West 2001. Assessing the validity of College Alcohol Problem Scale with African American undergraduates: Results of preliminary investigation. Journal of Alcohol and Drug Education. 46-3:4-17 Wu 2008. Validation and comparison of alcohol-screening instruments for identifying hazardous drinking in hospitalized patients in Taiwan. Alcohol Alcohol. 43-5:577-82 Young 2010. The Alcohol Use Disorders Identification Scale (AUDIT) normative scores for a multiracial sample of Rhodes University residence students. Journal of Child and Adolescent Mental Health. 22-1:15-23 Zavar 2015. The alcohol use disorders identification test (AUDIT): Reliability and validity of the Persian version. Asia Pacific Journal of Medical Toxicology. 4-1:37-42 |
| 108  AUDIT-3 | Aalto 2006. Effectiveness of structured questionnaires for screening heavy drinking in middle-aged women. Alcohol Clin Exp Res. 30-11:1884-8 Aalto 2009. AUDIT and its abbreviated versions in detecting heavy and binge drinking in a general population survey. 103-1-2:25-9 Blank 2015. Screening for hazardous alcohol use among university students using individual questions from the Alcohol Use Disorders Identification Test-Consumption. Drug and Alcohol Review. 34-5:540-548 Bradley 2003. Two brief alcohol-screening tests From the Alcohol Use Disorders Identification Test (AUDIT): validation in a female Veterans Affairs patient population. Arch Intern Med. 163-7:821-9 Bush 1998. The AUDIT alcohol consumption questions (AUDIT-C): an effective brief screening test for problem drinking. Ambulatory Care Quality Improvement Project (ACQUIP). Alcohol Use Disorders Identification Test. Arch Intern Med. 158-16:1789-95 Caviness 2009. Three brief alcohol screens for detecting hazardous drinking in incarcerated women. 70-1:50-4 CortÃ©s-TomÃ¡s 2016. Different versions of the Alcohol Use Disorders Identification Test (AUDIT) as screening instruments for underage binge drinking. Drug and Alcohol Dependence. 158-:52-59 Gordon 2001. Three questions can detect hazardous drinkers. The Journal of Family Practice. 50-4:313-320 Gual 2002. Audit-3 and audit-4: effectiveness of two short forms of the alcohol use disorders identification test. Alcohol Alcohol. 37-6:591-6 Kim 2013. The 5-item Alcohol Use Disorders Identification Test (AUDIT-5): An effective brief screening test for problem drinking, alcohol use disorders and alcohol dependence. Alcohol and Alcoholism. 48-1:68-73 Levola 2015. Screening for atâ€_risk drinking in a population reporting symptoms of depression: A validation of the AUDIT, AUDIT-C, and AUDIT-3. Alcoholism: Clinical and Experimental Research. 39-7:1186-1192 Meneses-Gaya 2010. Is the full version of the AUDIT really necessary? Study of the validity and internal construct of its abbreviated versions. Alcohol Clin Exp Res. 34-8:1417-24 O'Hare 2005. Comparing the AUDIT and 3 drinking indices as predictors of personal and social drinking problems in freshman first offenders. Journal of Alcohol and Drug Education. 49-3:37-61 Seth 2015. AUDIT, AUDIT-C, and AUDIT-3: Drinking patterns and screening for harmful, hazardous and dependent drinking in Katutura, Namibia. PLoS ONE. 10-3: Wu 2008. Validation and comparison of alcohol-screening instruments for identifying hazardous drinking in hospitalized patients in Taiwan. Alcohol Alcohol. 43-5:577-82 |
| 119  AUDIT-C | Aalto 2006. Effectiveness of structured questionnaires for screening heavy drinking in middle-aged women. Alcohol Clin Exp Res. 30-11:1884-8 Aalto 2009. AUDIT and its abbreviated versions in detecting heavy and binge drinking in a general population survey. 103-1-2:25-9 Aalto 2011. The Alcohol Use Disorders Identification Test (AUDIT) and its derivatives in screening for heavy drinking among the elderly. 26-9:881-5 Barry 2015. Evaluating the psychometric properties of the AUDIT-C among college students. Journal of Substance Use. 20-1:1-5 Bradley 2003. Two brief alcohol-screening tests From the Alcohol Use Disorders Identification Test (AUDIT): validation in a female Veterans Affairs patient population. Arch Intern Med. 163-7:821-9 Bush 1998. The AUDIT alcohol consumption questions (AUDIT-C): an effective brief screening test for problem drinking. Ambulatory Care Quality Improvement Project (ACQUIP). Alcohol Use Disorders Identification Test. Arch Intern Med. 158-16:1789-95 Caviness 2009. Three brief alcohol screens for detecting hazardous drinking in incarcerated women. 70-1:50-4 Clark 2014. Brief versus full alcohol use disorders identification test for the identification of alcohol misuse in critical illness. American Journal of Respiratory and Critical Care Medicine. 189-: CortÃ©s-TomÃ¡s 2016. Different versions of the Alcohol Use Disorders Identification Test (AUDIT) as screening instruments for underage binge drinking. Drug and Alcohol Dependence. 158-:52-59 Crawford 2013. Diagnostic efficiency of the AUDIT-C in U.S. veterans with military service since September 11, 2001. 132-1-2:101-6 Dawson 2005. The AUDIT-C: screening for alcohol use disorders and risk drinking in the presence of other psychiatric disorders. Comprehensive Psychiatry. 46-6:405-416 Dawson 2012. Comparative performance of the AUDIT-C in screening for DSM-IV and DSM-5 alcohol use disorders. 126-3:384-8 DeMartini 2012. Optimizing the use of the AUDIT for alcohol screening in college students. Psychological Assessment. 24-4:954-963 Frank 2008. Effectiveness of the AUDIT-C as a screening test for alcohol misuse in three race/ethnic groups. 23-6:781-7 Gordon 2001. Three questions can detect hazardous drinkers. The Journal of Family Practice. 50-4:313-320 GÃ³mez 2006. The diagnostic usefulness of AUDIT and AUDIT-C for detecting hazardous drinkers in the elderly. Aging & Mental Health. 10-5:558-561 Johnson 2013. Use of AUDITâ€_based measures to identify unhealthy alcohol use and alcohol dependence in primary care: A validation study. Alcoholism: Clinical and Experimental Research. 37-Suppl 1:E253-E259 Kawada 2011. The alcohol use disorders identification test: reliability study of the Japanese version. Alcohol. 45-3:205-7 Kelly 2009. Brief screens for detecting alcohol use disorder among 18-20 year old young adults in emergency departments: Comparing AUDIT-C, CRAFFT, RAPS4-QF, FAST, RUFT-Cut, and DSM-IV 2-Item Scale. 34-8:668-74 Kim 2013. The 5-item Alcohol Use Disorders Identification Test (AUDIT-5): An effective brief screening test for problem drinking, alcohol use disorders and alcohol dependence. Alcohol and Alcoholism. 48-1:68-73 Lapham 2014. Decreasing sensitivity of clinical alcohol screening with the AUDIT-C after repeated negative screens in VA clinics. Drug and Alcohol Dependence. 142-:209-215 Levola 2015. Screening for atâ€_risk drinking in a population reporting symptoms of depression: A validation of the AUDIT, AUDIT-C, and AUDIT-3. Alcoholism: Clinical and Experimental Research. 39-7:1186-1192 Lundin 2015. The Use of Alcohol Use Disorders Identification Test (AUDIT) in Detecting Alcohol Use Disorder and Risk Drinking in the General Population: Validation of AUDIT Using Schedules for Clinical Assessment in Neuropsychiatry. Alcoholism: Clinical and Experimental Research. 39-1:158-165 McCambridge 2009. Short forms of the AUDIT in a Web-based study of young drinkers. Drug and Alcohol Review. 28-1:18-24 McGinnis 2013. Comparing alcohol screening measures among HIV-infected and -uninfected men. Alcohol Clin Exp Res. 37-3:435-42 Meneses-Gaya 2010. Is the full version of the AUDIT really necessary? Study of the validity and internal construct of its abbreviated versions. Alcohol Clin Exp Res. 34-8:1417-24 Morojele 2015. Reliability and factor structure of the audit among male and female bar patrons in a rural area of South Africa. African Journal of Drug and Alcohol Studies. 14-1:23-35 Neumann 2012. Does the Alcohol Use Disorders Identification Test â€“ Consumption identify the same patient population as the full 10-item Alcohol Use Disorders Identification Test?. Journal of Substance Abuse Treatment. 43-1:80-85 O'Hare 1999. Validating the alcohol use disorder identification test with college first-offenders. Journal of Substance Abuse Treatment. 17-1-2:113-119 Rodriguez-Martos 2007. Does the short form of the Alcohol Use Disorders Identification Test (AUDIT-C) work at a trauma emergency department?. Subst Use Misuse. 42-6:923-32 Rumpf 2002. Screening for alcohol use disorders and at-risk drinking in the general population: psychometric performance of three questionnaires. Alcohol Alcohol. 37-3:261-8 Rumpf 2013. Screening questionnaires for problem drinking in adolescents: performance of AUDIT, AUDIT-C, CRAFFT and POSIT. 19-3:121-7 Saitz 2012. The ability of single screening questions for unhealthy alcohol and other drug use to identify substance dependence in primary care. Journal of General Internal Medicine. 27-:S329-S330 Saitz 2014. The ability of single screening questions for unhealthy alcohol and other drug use to identify substance dependence in primary care. 75-1:153-7 Seale 2006. Primary Care Validation of a Single Screening Question for Drinkers. Journal of Studies on Alcohol. 67-5:778-784 Seth 2015. AUDIT, AUDIT-C, and AUDIT-3: Drinking patterns and screening for harmful, hazardous and dependent drinking in Katutura, Namibia. PLoS ONE. 10-3: Smith 2009. Primary care validation of a single-question alcohol screening test. 24-7:783-8 Strauss 2009. Screening patients in busy hospital-based HIV care centers for hazardous and harmful drinking patterns: the identification of an optimal screening tool. J Int Assoc Physicians AIDS Care (Chic). 8-6:347-53 Tsai 2005. Alcohol Use Disorders Identification Test (AUDIT): Establishment of Cut-off Scores in a Hospitalized Chinese Population. Alcoholism: Clinical and Experimental Research. 29-1:53-57 Vitesnikova 2014. Use of AUDIT-C as a tool to identify hazardous alcohol consumption in admitted trauma patients. Injury. 45-9:1440-4 Wu 2008. Validation and comparison of alcohol-screening instruments for identifying hazardous drinking in hospitalized patients in Taiwan. Alcohol Alcohol. 43-5:577-82 |
| 418  B-MAST | Cherpitel 1995. Ethnic differences in performance of screening instruments for identifying harmful drinking and alcohol dependence in the emergency room. Alcohol Clin Exp Res. 19-3:628-34 Cherpitel 1997. Comparison of screening instruments for alcohol problems between black and white emergency room patients from two regions of the country. Alcohol Clin Exp Res. 21-8:1391-7 Cherpitel 2000. Screening instruments for alcohol problems: a comparison of cut points between Mexican American and Mexican patients in the emergency room. Subst Use Misuse. 35-10:1419-30 Cherpitel 2001. Screening for alcohol problems: A comparison of instrument performance among black emergency department and primary care patients. Journal of Substance Use. 5-4:290-297 Cremonte 2008. Performance of screening instruments for alcohol use disorders in emergency departments patients in Argentina. Substance Use & Misuse. 43-1:125-138 Fuller 1994. Screening patients with traumatic brain injuries for substance abuse. J Neuropsychiatry Clin Neurosci. 6-2:143-6 Garzotto 1988. Validation of a screening questionnaire for alcoholism (MAST) in an Italian sample. Comprehensive Psychiatry. 29-3:323-329 Smith 1987. Screening for problem drinking in college freshmen. Journal of American College Health. 36-2:89-94 Soderstrom 1997. The accuracy of the CAGE, the Brief Michigan Alcoholism Screening Test, and the Alcohol Use Disorders Identification Test in screening trauma center patients for alcoholism. J Trauma. 43-6:962-9 |
| 419  BAC | Fillmore 2011. Defining "binge" drinking as five drinks per occasion or drinking to a .08% BAC: which is more sensitive to risk?. Am J Addict. 20-5:468-75 Gentilello 1999. Detection of acute alcohol intoxication and chronic alcohol dependence by trauma center staff. J Trauma. 47-6:1131-5; discussion 1135-9 Gijsbers 1991. Does a blood alcohol level of 0.15 or more identify accurately problem drinkers in a drink-driver population?. Med J Aust. 154-7:448-52 Plackett 2015. Screening for at-risk drinking behavior in trauma patients. J Am Osteopath Assoc. 115-6:376-82 Ryb 1999. Use of blood alcohol concentration and laboratory tests to detect current alcohol dependence in trauma center patients. J Trauma. 47-5:874-9; discussion 879-80 Savola 2004. Blood alcohol is the best indicator of hazardous alcohol drinking in young adults and working-age patients with trauma. Alcohol Alcohol. 39-4:340-5 Soderstrom 1997. The accuracy of the CAGE, the Brief Michigan Alcoholism Screening Test, and the Alcohol Use Disorders Identification Test in screening trauma center patients for alcoholism. J Trauma. 43-6:962-9 Soderstrom 1998. Screening trauma patients for alcoholism according to NIAAA guidelines with alcohol use disorders identification test questions. Alcohol Clin Exp Res. 22-7:1470-5 |
| 460  CAGE | Aalto 2006. Effectiveness of structured questionnaires for screening heavy drinking in middle-aged women. Alcohol Clin Exp Res. 30-11:1884-8 Agabio 2007. Alcohol use disorders, and at-risk drinking in patients affected by a mood disorder, in Cagliari, Italy: sensitivity and specificity of different questionnaires. Alcohol Alcohol. 42-6:575-81 Aithal 1998. Measurement of carbohydrate-deficient transferrin (CDT) in a general medical clinic: is this test useful in assessing alcohol consumption. Alcohol Alcohol. 33-3:304-9 Amaral 2004. [Evaluation of a screening test for alcohol-related problems (CAGE) among employees of the Campus of the University of Sao Paulo]. Rev Bras Psiquiatr. 26-3:156-63 Ashman 2004. Screening for substance abuse in individuals with traumatic brain injury. 18-2:191-202 Beresford 1990. Comparison of CAGE questionnaire and computer-assisted laboratory profiles in screening for covert alcoholism. Lancet. 336-8713:482-5 Bernadt 1983. Drinking histories: Are they accurate?. Neuropharmacology. 22-4:571-572 Bradley 1998. Screening for problem drinking: comparison of CAGE and AUDIT. Ambulatory Care Quality Improvement Project (ACQUIP). Alcohol Use Disorders Identification Test. 13-6:379-88 Bush 1987. Screening for alcohol abuse using the CAGE questionnaire. Am J Med. 82-2:231-5 Castells 2005. Validity of the CAGE questionnaire for screening alcohol-dependent inpatients on hospital wards. Rev Bras Psiquiatr. 27-1:54-7 Chan 1994. Detection by the CAGE of alcoholism or heavy drinking in primary care outpatients and the general population. J Subst Abuse. 6-2:123-35 Chen 2016. Validity of the cage questionnaire for men who have sex with men (msm) in china. Drug and Alcohol Dependence. -: Cherpitel 1995. Ethnic differences in performance of screening instruments for identifying harmful drinking and alcohol dependence in the emergency room. Alcohol Clin Exp Res. 19-3:628-34 Cherpitel 1997. Comparison of screening instruments for alcohol problems between black and white emergency room patients from two regions of the country. Alcohol Clin Exp Res. 21-8:1391-7 Cherpitel 1998. Differences in performance of screening instruments for problem drinking among blacks, whites and Hispanics in an emergency room population. J Stud Alcohol. 59-4:420-6 Cherpitel 2000. Screening instruments for alcohol problems: a comparison of cut points between Mexican American and Mexican patients in the emergency room. Subst Use Misuse. 35-10:1419-30 Cherpitel 2001. Screening for alcohol problems: A comparison of instrument performance among black emergency department and primary care patients. Journal of Substance Use. 5-4:290-297 Christensen 2005. A brief case-finding questionnaire for common mental disorders: the CMDQ. 22-4:448-57 Clements 1998. A critical evaluation of several alcohol screening instruments using the CIDI-SAM as a criterion measure. Alcohol Clin Exp Res. 22-5:985-93 Clements 2002. Diagnostic accuracy and factor structure of the AAS and APS scales of the MMPI-2. J Pers Assess. 79-3:564-82 Cook 2005. Alcohol screening in young persons attending a sexually transmitted disease clinic. Comparison of AUDIT, CRAFFT, and CAGE instruments. 20-1:1-6 Cornel 1994. Problem drinking in a general practice population: the construction of an interval scale for severity of problem drinking. J Stud Alcohol. 55-4:466-70 Corradi-Webster 2005. [Performance assessment of CAGE screening test among psychiatric outpatients]. Rev Lat Am Enfermagem. 13 Spec No-:1213-8 Cremonte 2008. Performance of screening instruments for alcohol use disorders in emergency departments patients in Argentina. Substance Use & Misuse. 43-1:125-138 Cremonte 2010. Psychometric properties of alcohol screening tests in the emergency department in Argentina, Mexico and the United States. 35-9:818-25 Crowe 1997. The utility of the Brief MAST' and the CAGE' in identifying alcohol problems: results from national high-risk and community samples. Arch Fam Med. 6-5:477-83 Dervaux 2006. Validity of the CAGE questionnaire in schizophrenic patients with alcohol abuse and dependence. Schizophr Res. 81-2-3:151-5 Drake 1990. Diagnosis of Alcohol Use Disorders in Schizophrenia. Schizophrenia Bulletin. 16-1:57-67 Escobar 1995. Diagnostic tests for alcoholism in primary health care: compared efficacy of different instruments. 40-2:151-8 Fertig 1993. CAGE as a predictor of hazardous alcohol consumption in U.S. Army personnel. Alcohol Clin Exp Res. 17-6:1184-7 Forsberg 2002. Screening of binge drinking among patients on an emergency surgical ward. Alcohol. 27-2:77-82 Frank 2008. Effectiveness of the AUDIT-C as a screening test for alcohol misuse in three race/ethnic groups. 23-6:781-7 Fuller 1994. Screening patients with traumatic brain injuries for substance abuse. J Neuropsychiatry Clin Neurosci. 6-2:143-6 Geneste 2012. CAGE, RAPS4, RAPS4-QF and AUDIT screening tests for men and women admitted for acute alcohol intoxication to an emergency department: are standard thresholds appropriate?. Alcohol Alcohol. 47-3:273-81 Gentilello 1999. Detection of acute alcohol intoxication and chronic alcohol dependence by trauma center staff. J Trauma. 47-6:1131-5; discussion 1135-9 Girela 1994. Comparison of the CAGE questionnaire versus some biochemical markers in the diagnosis of alcoholism. Alcohol Alcohol. 29-3:337-43 Gomez 2001. Diagnostic usefulness of carbohydrate-deficient transferrin for detecting alcohol-related problems in hospitalized patients. Alcohol Alcohol. 36-3:266-70 GÃ³mez 2006. The diagnostic usefulness of AUDIT and AUDIT-C for detecting hazardous drinkers in the elderly. Aging & Mental Health. 10-5:558-561 Hays 1995. Response burden, reliability, and validity of the CAGE, Short MAST, and AUDIT alcohol screening measures. Behavior Research Methods, Instruments & Computers. 27-2:277-280 Hays 2001. Comparison of the Rost and the CAGE alcohol screening instruments in young adults. Subst Use Misuse. 36-5:639-51 Heck 1995. Using the CAGE to screen for drinking-related problems in college students. J Stud Alcohol. 56-3:282-6 Hinkin 2001. Screening for drug and alcohol abuse among older adults using a modified version of the CAGE. Am J Addict. 10-4:319-26 Hodgson 2003. Fast screening for alcohol misuse. 28-8:1453-63 Indran 1995. Usefulness of the "CAGE" in Malaysia. Singapore Med J. 36-2:194-6 Johnson 2005. Reliability and concurrent validity of the CAGE screening questions: A comparison of lesbians and heterosexual women. Subst Use Misuse. 40-5:657-69 Joseph 1995. Screening for alcohol use disorders in the nursing home. 43-4:368-73 Kelly 2002. A comparison of alcohol screening instruments among under-aged drinkers treated in emergency departments. Alcohol Alcohol. 37-5:444-50 Kelly 2004. Alcohol use disorders among emergency department-treated older adolescents: a new brief screen (RUFT-Cut) using the AUDIT, CAGE, CRAFFT, and RAPS-QF. Alcohol Clin Exp Res. 28-5:746-53 Kim 2013. The 5-item Alcohol Use Disorders Identification Test (AUDIT-5): An effective brief screening test for problem drinking, alcohol use disorders and alcohol dependence. Alcohol and Alcoholism. 48-1:68-73 King 1986. At risk drinking among general practice attenders: Validation of the CAGE questionnaire. Psychological Medicine. 16-1:213-217 Knight 2003. Validity of brief alcohol screening tests among adolescents: A comparison of the AUDIT, POSIT, CAGE, and CRAFFT. Alcoholism: Clinical and Experimental Research. 27-1:67-73 Koppes 2004. Comparison of short questionnaires on alcohol drinking behavior in a nonclinical population of 36-year-old men and women. Subst Use Misuse. 39-7:1041-60 Kuo 1999. Validity of the CAGE questionnaire in a primary care setting in Taiwan: A cross-cultural examination. Chinese Journal of Public Health. 18-2:87-94 Liskow 1995. Validity of the CAGE questionnaire in screening for alcohol dependence in a walk-in (triage) clinic. J Stud Alcohol. 56-3:277-81 Luttrell 1997. Screening for alcohol misuse in older people. 12-12:1151-4 MacKenzie 1996. Identifying hazardous or harmful alcohol use in medical admissions: a comparison of audit, cage and brief mast. Alcohol Alcohol. 31-6:591-9 Malet 2005. Validity of the CAGE questionnaire in hospital. Eur Psychiatry. 20-7:484-9 Masur 1983. Validation of the "CAGE" alcoholism screening test in a Brazilian psychiatric inpatient hospital setting. Braz J Med Biol Res. 16-3:215-8 Matano 2003. Assessment of binge drinking of alcohol in highly educated employees. 28-7:1299-310 McQuade 2000. Detecting symptoms of alcohol abuse in primary care settings. Arch Fam Med. 9-9:814-21 Meneses-Gaya 2010. Is the full version of the AUDIT really necessary? Study of the validity and internal construct of its abbreviated versions. Alcohol Clin Exp Res. 34-8:1417-24 Midanik 1998. Alcohol and drug CAGE screeners for pregnant, low-income women: the California Perinatal Needs Assessment. Alcohol Clin Exp Res. 22-1:121-5 Mischke 1987. Reliability and validity of the MAST, Mortimer-Filkins Questionnaire and CAGE in DWI assessment. J Stud Alcohol. 48-5:492-501 Moraes 2005. Assessing alcohol misuse during pregnancy: evaluating psychometric properties of the CAGE, T-ACE and TWEAK in a Brazilian setting. J Stud Alcohol. 66-2:165-73 Morton 1996. Performance of alcoholism screening questionnaires in elderly veterans. Am J Med. 101-2:153-9 Myerholtz 1997. Screening DUI offenders for alcohol problems: Psychometric assessment of the substance abuse subtle screening inventory. Psychology of Addictive Behaviors. 11-3:155-165 Naik 1995. How can we detect sick elderly excessive drinkers?. International Journal of Geriatric Psychiatry. 10-12:1063-1066 Nystrom 1993. Screening for heavy drinking and alcohol-related problems in young university students: the CAGE, the Mm-MAST and the trauma score questionnaires. J Stud Alcohol. 54-5:528-33 O'Hare 1997. Predicting problem drinking in college students: gender differences and the CAGE questionnaire. 22-1:13-21 Oliveira 2011. Alcohol screening instruments in elderly male: a population-based survey in metropolitan Sao Paulo, Brazil. Rev Bras Psiquiatr. 33-4:347-52 Philpot 2003. Screening for problem drinking in older people referred to a mental health service: a comparison of CAGE and AUDIT. Aging Ment Health. 7-3:171-5 Richoux 2011. Alcohol use disorders in the emergency ward: Choice of the best mode of assessment and identification of at-risk situations. International Journal of Emergency Medicine. 4-1: Rosenberg 1998. Dartmouth Assessment of Lifestyle Instrument (DALI): a substance use disorder screen for people with severe mental illness. Am J Psychiatry. 155-2:232-8 Ross 1994. Identification of alcohol disorders at a university mental health centre, using the CAGE. Journal of Alcohol and Drug Education. 39-3:119-126 Rumpf 1998. Screening questionnaires in the detection of hazardous alcohol consumption in the general hospital: direct or disguised assessment?. J Stud Alcohol. 59-6:698-703 Russell 1994. Screening for pregnancy risk-drinking. Alcohol Clin Exp Res. 18-5:1156-61 Russell 1996. Detecting risk drinking during pregnancy: a comparison of four screening questionnaires. Am J Public Health. 86-10:1435-9 Ryou 2012. Usefulness of Alcohol-screening Instruments in Detecting Problem Drinking among Elderly Male Drinkers. Korean J Fam Med. 33-3:126-33 Saitz 1999. Alcohol abuse and dependence in Latinos living in the United States: validation of the CAGE (4M) questions. Arch Intern Med. 159-7:718-24 Saremi 2001. Validity of the CAGE questionnaire in an American Indian population. J Stud Alcohol. 62-3:294-300 Seppa 1995. Effectiveness of the Alcohol Use Disorders Identification Test in occupational health screenings. Alcohol Clin Exp Res. 19-4:999-1003 Seppa 1998. Five-shot questionnaire on heavy drinking. Alcohol Clin Exp Res. 22-8:1788-91 Smith 1987. Screening for problem drinking in college freshmen. Journal of American College Health. 36-2:89-94 Soderstrom 1997. The accuracy of the CAGE, the Brief Michigan Alcoholism Screening Test, and the Alcohol Use Disorders Identification Test in screening trauma center patients for alcoholism. J Trauma. 43-6:962-9 Sokol 1989. The T-ACE questions: practical prenatal detection of risk-drinking. 160-4:863-8; discussion 868-70 Steinbauer 1998. Ethnic and sex bias in primary care screening tests for alcohol use disorders. Ann Intern Med. 129-5:353-62 Taylor 2016. Improving Alcohol Screening for College Students: Screening for Alcohol Misuse amongst College Students with a Simple Modification to CAGE. J Am Coll Health. -:0 Thiesen 2010. Biological markers of problem drinking in homeless patients. 35-3:260-2 Vinson 2004. Comfortably engaging: which approach to alcohol screening should we use?. Ann Fam Med. 2-5:398-404 Werner 1994. Screening for problem drinking among college freshmen. J Adolesc Health. 15-4:303-10 Werner 1996. Concurrent and prospective screening for problem drinking among college students. J Adolesc Health. 18-4:276-85 West 2001. Assessing the validity of College Alcohol Problem Scale with African American undergraduates: Results of preliminary investigation. Journal of Alcohol and Drug Education. 46-3:4-17 Wetterling 1998. Comparison of cage and mast with the alcohol markers CDT, gamma-GT, ALAT, ASAT and MCV. Alcohol Alcohol. 33-4:424-30 Wu 2008. Validation and comparison of alcohol-screening instruments for identifying hazardous drinking in hospitalized patients in Taiwan. Alcohol Alcohol. 43-5:577-82 |
| 519  CDT | Godart 2005. Carbohydrate-deficient transferrin and gamma-glutamyl transpeptidase in the evaluation of alcohol consumption. A five-year retrospective study of 633 outpatients in a single center. Gastroenterol Clin Biol. 29-2:113-6 Kanitz 1994. New state markers for alcoholism. Comparison of carbohydrate deficient transferrin (CDT) and alcohol mediated (triantennary) transferrin (AMT). Prog Neuropsychopharmacol Biol Psychiatry. 18-3:431-46 Lesch 1996. Carbohydrate-deficient transferrin as a screening marker for drinking in a general hospital population. Alcohol Alcohol. 31-3:249-56 Piano 2014. Assessment of alcohol consumption in liver transplant candidates and recipients: the best combination of the tools available. Liver Transpl. 20-7:815-22 Reynaud 1998. Usefulness of carbohydrate-deficient transferrin in alcoholic patients with normal gamma-glutamyltranspeptidase. Alcohol Clin Exp Res. 22-3:615-8 Reynaud 2000. Objective diagnosis of alcohol abuse: compared values of carbohydrate-deficient transferrin (CDT), gamma-glutamyl transferase (GGT), and mean corpuscular volume (MCV). Alcohol Clin Exp Res. 24-9:1414-9 Schellenberg 1989. Evaluation of carbohydrate-deficient transferrin compared with Tf index and other markers of alcohol abuse. Alcohol Clin Exp Res. 13-5:605-10 Schmidt 1997. Superiority of carbohydrate-deficient transferrin to gamma-glutamyltransferase in detecting relapse in alcoholism. Am J Psychiatry. 154-1:75-80 Wetterling 1998. Comparison of cage and mast with the alcohol markers CDT, gamma-GT, ALAT, ASAT and MCV. Alcohol Alcohol. 33-4:424-30 |
| 525  CDtech | Aithal 1998. Measurement of carbohydrate-deficient transferrin (CDT) in a general medical clinic: is this test useful in assessing alcohol consumption. Alcohol Alcohol. 33-3:304-9 Allen 1999. Contribution of carbohydrate deficient transferrin to gamma glutamyl transpeptidase in evaluating progress of patients in treatment for alcoholism. Alcohol Clin Exp Res. 23-1:115-20 Anton 1994. Carbohydrate-deficient transferrin and !g-glutamyltransferase as markers of heavy alcohol consumption: Gender differences. Alcoholism: Clinical and Experimental Research. 18-3:747-754 Anton 2001. Comparison of Bio-Rad %CDT TIA and CDTect as laboratory markers of heavy alcohol use and their relationships with gamma-glutamyltransferase. Clin Chem. 47-10:1769-75 Anton 2002. Carbohydrate-deficient transferrin and gamma-glutamyltransferase for the detection and monitoring of alcohol use: results from a multisite study. Alcohol Clin Exp Res. 26-8:1215-22 Anttila 2003. A new modified gamma-%CDT method improves the detection of problem drinking: studies in alcoholics with or without liver disease. Clin Chim Acta. 338-1-2:45-51 Anttila 2005. Biomarkers of alcohol consumption in patients classified according to the degree of liver disease severity. Scand J Clin Lab Invest. 65-2:141-51 Bell 1993. Serum carbohydrate-deficient transferrin as a marker of alcohol consumption in patients with chronic liver diseases. Alcohol Clin Exp Res. 17-2:246-52 Bell 1994. Carbohydrate-deficient transferrin and other markers of high alcohol consumption: a study of 502 patients admitted consecutively to a medical department. Alcohol Clin Exp Res. 18-5:1103-8 Brathen 2001. Detection of alcohol abuse in neurological patients: variables of clinical relevance to the accuracy of the %CDT-TIA and CDTect methods. Alcohol Clin Exp Res. 25-1:46-53 Conigrave 2002. CDT, GGT, and AST as markers of alcohol use: the WHO/ISBRA collaborative project. Alcohol Clin Exp Res. 26-3:332-9 Fagerberg 1994. Is carbohydrate-deficient transferrin in serum useful for detecting excessive alcohol consumption in hypertensive patients?. Clin Chem. 40-11 Pt 1:2057-63 Gronbaek 1995. Carbohydrate-deficient transferrin--a valid marker of alcoholism in population studies? Results from the Copenhagen City Heart Study. Alcohol Clin Exp Res. 19-2:457-61 Halm 1999. Relative versus absolute carbohydrate-deficient transferrin as a marker of alcohol consumption in patients with acute alcoholic hepatitis. Alcohol Clin Exp Res. 23-10:1614-8 Hannuksela 1992. Evaluation of plasma cholesteryl ester transfer protein (CETP) activity as a marker of alcoholism. Alcohol Alcohol. 27-5:557-62 Korzec 2009. Validation of the Bayesian Alcoholism Test compared to single biomarkers in detecting harmful drinking. Alcohol and Alcoholism. 44-4:398-402 Lott 1998. Reported alcohol consumption and the serum carbohydrate-deficient transferrin test in third-year medical students. Clin Chim Acta. 276-2:129-41 Meerkerk 1999. Comparing the diagnostic accuracy of carbohydrate-deficient transferrin, Î³-Glutamyltransferase, and mean cell volume in a general practice population. Alcoholism: Clinical and Experimental Research. 23-6:1052-1059 Meregalli 1995. Carbohydrate-deficient transferrin in alcohol and nonalcohol abusers with liver disease. Alcohol Clin Exp Res. 19-6:1525-7 Mitchell 1997. Carbohydrate deficient transferrin in detecting relapse in alcohol dependence. 48-2:97-103 Niemela 1995. Carbohydrate-deficient transferrin as a marker of alcohol abuse: relationship to alcohol consumption, severity of liver disease, and fibrogenesis. Alcohol Clin Exp Res. 19-5:1203-8 Nilssen 1992. New alcohol markersâ€”how useful are they in population studies: The Svalbard study 1988â€“89. Alcoholism: Clinical and Experimental Research. 16-1:82-86 Nystrom 1992. Carbohydrate-deficient transferrin (CDT) in serum as a possible indicator of heavy drinking in young university students. Alcohol Clin Exp Res. 16-1:93-7 Oslin 1998. Clinical correlations with carbohydrate-deficient transferrin levels in women with alcoholism. Alcohol Clin Exp Res. 22-9:1981-5 Rublo 1997. Carbohydrate-deficient transferrin as a marker of alcohol consumption in male patients with liver disease. Alcohol Clin Exp Res. 21-5:923-7 Savola 2004. Blood alcohol is the best indicator of hazardous alcohol drinking in young adults and working-age patients with trauma. Alcohol Alcohol. 39-4:340-5 Schmitt 1998. Carbohydrate-deficient transferrin is not a useful marker for the detection of chronic alcohol abuse. Eur J Clin Invest. 28-8:615-21 Sharpe 1996. Biochemical markers of alcohol abuse. Qjm. 89-2:137-44 Siegfried 2001. Profile of drinking behaviour and comparison of self-report with the CAGE questionnaire and carbohydrate-deficient transferrin in a rural Lesotho community. Alcohol Alcohol. 36-3:243-8 Sillanaukee 1998. Carbohydrate-deficient transferrin and conventional alcohol markers as indicators for brief intervention among heavy drinkers in primary health care. Alcohol Clin Exp Res. 22-4:892-6 Sillanaukee 1999. Sialic acid: new potential marker of alcohol abuse. Alcohol Clin Exp Res. 23-6:1039-43 Sorvajarvi 1996. Sensitivity and specificity of carbohydrate-deficient transferrin as a marker of alcohol abuse are significantly influenced by alterations in serum transferrin: comparison of two methods. Alcohol Clin Exp Res. 20-3:449-54 Stauber 1995. Evaluation of carbohydrate-deficient transferrin for detection of alcohol abuse in patients with liver dysfunction. Alcohol Alcohol. 30-2:171-6 Stowell 1997. Comparison of two commercial test kits for quantification of serum carbohydrate-deficient transferrin. Alcohol Alcohol. 32-4:507-16 Tonnesen 1999. Is carbohydrate deficient transferrin a useful marker of harmful alcohol intake among surgical patients?. Eur J Surg. 165-6:522-7 Yeastedt 1998. Female alcoholic outpatients and female college students: a correlational study of self-reported alcohol consumption and carbohydrate-deficient transferrin levels. J Stud Alcohol. 59-5:555-9 Yersin 1995. Screening for excessive alcohol drinking. Comparative value of carbohydrate-deficient transferrin, gamma-glutamyltransferase, and mean corpuscular volume. Arch Intern Med. 155-17:1907-11 Zierau 2005. Validation of a self-administered modified CAGE test (CAGE-C) in a somatic hospital ward: comparison with biochemical markers. Scand J Clin Lab Invest. 65-7:615-22 |
| 540  CDT-MCV | Mundle 1999. Biological markers as indicators for relapse in alcohol-dependent patients. Addiction Biology. 4-2:209-214 Mundle 2000. Sex differences of carbohydrate-deficient transferrin, gamma-glutamyltransferase, and mean corpuscular volume in alcohol-dependent patients. Alcohol Clin Exp Res. 24-9:1400-5 Reynaud 2000. Objective diagnosis of alcohol abuse: compared values of carbohydrate-deficient transferrin (CDT), gamma-glutamyl transferase (GGT), and mean corpuscular volume (MCV). Alcohol Clin Exp Res. 24-9:1414-9 Rinck 2007. Combinations of carbohydrate-deficient transferrin, mean corpuscular erythrocyte volume, gamma-glutamyltransferase, homocysteine and folate increase the significance of biological markers in alcohol dependent patients. 89-1:60-5 Sillanaukee 1998. Carbohydrate-deficient transferrin and conventional alcohol markers as indicators for brief intervention among heavy drinkers in primary health care. Alcohol Clin Exp Res. 22-4:892-6 |
| 561  CIDI | Haro 2006. Concordance of the Composite International Diagnostic Interview Version 3.0 (CIDI 3.0) with standardized clinical assessments in the WHO World Mental Health surveys. Int J Methods Psychiatr Res. 15-4:167-80 Jayasekera 2011. Comparison of the Composite International Diagnostic interview (CIDI-Auto) with clinical diagnosis in a suicidal population. Arch Suicide Res. 15-1:43-55 – version 2.1 Kessler 2009. National comorbidity survey replication adolescent supplement (NCS-A): III. Concordance of DSM-IV/CIDI diagnoses with clinical reassessments. 48-4:386-99 – (Version 3)  Quintana 2007. Validity and limitations of the Brazilian version of the Composite International Diagnostic Interview (CIDI 2.1). Revista brasileira de psiquiatria (São Paulo, Brazil : 1999). 29. 18-22. 10.1590/S1516-44462007000100007. Ramchand 2009. Alcohol abuse and illegal drug use among Los Angeles County trauma patients: prevalence and evaluation of single item screener. J Trauma. 66-5:1461-7 |
| 566  CRAFFT | Cook 2005. Alcohol screening in young persons attending a sexually transmitted disease clinic. Comparison of AUDIT, CRAFFT, and CAGE instruments. 20-1:1-6 Kelly 2004. Alcohol use disorders among emergency department-treated older adolescents: a new brief screen (RUFT-Cut) using the AUDIT, CAGE, CRAFFT, and RAPS-QF. Alcohol Clin Exp Res. 28-5:746-53 Kelly 2009. Brief screens for detecting alcohol use disorder among 18-20 year old young adults in emergency departments: Comparing AUDIT-C, CRAFFT, RAPS4-QF, FAST, RUFT-Cut, and DSM-IV 2-Item Scale. 34-8:668-74 Knight 2003. Validity of brief alcohol screening tests among adolescents: A comparison of the AUDIT, POSIT, CAGE, and CRAFFT. Alcoholism: Clinical and Experimental Research. 27-1:67-73 Mitchell 2014. The CRAFFT cut-points and DSM-5 criteria for alcohol and other drugs: a reevaluation and reexamination. Subst Abus. 35-4:376-80 Rumpf 2013. Screening questionnaires for problem drinking in adolescents: performance of AUDIT, AUDIT-C, CRAFFT and POSIT. 19-3:121-7 Subramaniam 2010. Validity of a brief screening instrument-CRAFFT in a multiethnic Asian population. 35-12:1102-4 |
| 714  DAST | Bryce 2015. Screening for Substance Use Disorders Following Traumatic Brain Injury: Examining the Validity of the AUDIT and the DAST. Journal of Head Trauma Rehabilitation. 30-5:E40-E48 Carey 2003. Psychometric evaluation of the alcohol use disorders identification test and short drug abuse screening test with psychiatric patients in India. J Clin Psychiatry. 64-7:767-74 Dyson 1998. Efficiency and validity of commonly used substance abuse screening instruments in public psychiatric patients. Journal of Addictive Diseases. 17-2:57-76 Gavin 1989. Diagnostic validity of the drug abuse screening test in the assessment of DSM-III drug disorders. Br J Addict. 84-3:301-7 McCann 2000. Reliability and validity of screening instruments for drug and alcohol abuse in adults seeking evaluation for attention-deficit/hyperactivity disorder. Am J Addict. 9-1:1-9 Rosenberg 1998. Dartmouth Assessment of Lifestyle Instrument (DALI): a substance use disorder screen for people with severe mental illness. Am J Psychiatry. 155-2:232-8 Saitz 2014. The ability of single screening questions for unhealthy alcohol and other drug use to identify substance dependence in primary care. 75-1:153-7 Skinner 1982. The drug abuse screening test. 7-4:363-71 Staley 1990. Psychometric properties of the Drug Abuse Screening Test in a psychiatric patient population. 15-3:257-64 |
| 715  DAST-10 | Cocco 1998. Psychometric properties of the drug abuse screening test in psychiatric outpatients. Psychological Assessment. 10-4:408-414 Evren 2013. Psychometric properties of the drug abuse screening test (DAST-10) in heroin dependent adults and adolescents with drug use disorder. Dusunen Adam. 26-4:351-359 Evren 2014. Psychometric properties of the Turkish versions of the Drug Use Disorders Identification Test (DUDIT) and the Drug Abuse Screening Test (DAST-10) in the prison setting. J Psychoactive Drugs. 46-2:140-6 Grekin 2010. Drug use during pregnancy: validating the Drug Abuse Screening Test against physiological measures. Psychol Addict Behav. 24-4:719-23 Hearon 2015. Improving the efficiency of drug use disorder screening in psychiatric settings: validation of a single-item screen. Am J Drug Alcohol Abuse. 41-2:173-6 Lam 2015. Validation of the Drug Abuse Screening Test (DAST-10): A study on illicit drug use among Chinese pregnant women. Sci Rep. 5-:11420 Maisto 2000. Use of the AUDIT and the DAST-10 to identify alcohol and drug use disorders among adults with a severe and persistent mental illness. 12-2:186-92 Smith 2010. A single-question screening test for drug use in primary care. Arch Intern Med. 170-13:1155-60 Villalobos-Gallegos 2015. Psychometric and diagnostic properties of the Drug Abuse Screening Test (DAST): Comparing the DAST-20 vs. the DAST-10. Salud Mental. 38-2:89-94 |
| 739  DUDIT | Berg 2011. Diagnosis after an acute psychiatric inpatient stay: How do psychotic and non-psychotic diagnoses relate to the results of psychometric tests of substance abuse?. Journal of Psychiatric Intensive Care. 7-1:11-16 Berman 2005. Evaluation of the Drug Use Disorders Identification Test (DUDIT) in Criminal Justice and Detoxification Settings and in a Swedish Population Sample. European Addiction Research. 11-1:22-31 Durbeej 2010. Validation of the Alcohol Use Disorders Identification Test and the Drug Use Disorders Identification Test in a Swedish sample of suspected offenders with signs of mental health problems: Results from the Mental Disorder, Substance Abuse and Crime study. Journal of Substance Abuse Treatment. 39-4:364-377 Evren 2014. Psychometric properties of the Turkish versions of the Drug Use Disorders Identification Test (DUDIT) and the Drug Abuse Screening Test (DAST-10) in the prison setting. J Psychoactive Drugs. 46-2:140-6 Gundersen 2013. Evaluation of the Alcohol Use Disorders Identification Test and the Drug Use Disorders Identification Test among patients at a Norwegian psychiatric emergency ward. European Addiction Research. 19-5:252-260 Hildebrand 2015. Exploration of the (Interrater) Reliability and Latent Factor Structure of the Alcohol Use Disorders Identification Test (AUDIT) and the Drug Use Disorders Identification Test (DUDIT) in a Sample of Dutch Probationers. Subst Use Misuse. 50-10:1294-306 Kader 2012. A preliminary investigation of the AUDIT and DUDIT in comparison to biomarkers for alcohol and drug use among HIV-infected clinic attendees in Cape Town, South Africa. Afr J Psychiatry (Johannesbg). 15-5:346-51 Matuszka 2014. Psychometric characteristics of the Drug Use Disorders Identification Test (DUDIT) and the Drug Use Disorders Identification Test-Extended (DUDIT-E) among young drug users in Hungary. Int J Behav Med. 21-3:547-55 Nesvag 2010. The use of screening instruments for detecting alcohol and other drug use disorders in first-episode psychosis. 177-1-2:228-34 Sinadinovic 2010. Internet-based assessment and self-monitoring of problematic alcohol and drug use. 35-5:464-70 Sinadinovic 2011. Population screening of risky alcohol and drug use via Internet and Interactive Voice response (IVR): A feasibility and psychometric study in a random sample. Drug and Alcohol Dependence. 114-1:55-60 |
| 885  GGT | Aithal 1998. Measurement of carbohydrate-deficient transferrin (CDT) in a general medical clinic: is this test useful in assessing alcohol consumption. Alcohol Alcohol. 33-3:304-9 Allen 1999. Contribution of carbohydrate deficient transferrin to gamma glutamyl transpeptidase in evaluating progress of patients in treatment for alcoholism. Alcohol Clin Exp Res. 23-1:115-20 Anton 1994. Carbohydrate-deficient transferrin and !g-glutamyltransferase as markers of heavy alcohol consumption: Gender differences. Alcoholism: Clinical and Experimental Research. 18-3:747-754 Anton 2001. Comparison of Bio-Rad %CDT TIA and CDTect as laboratory markers of heavy alcohol use and their relationships with gamma-glutamyltransferase. Clin Chem. 47-10:1769-75 Anton 2002. Carbohydrate-deficient transferrin and gamma-glutamyltransferase for the detection and monitoring of alcohol use: results from a multisite study. Alcohol Clin Exp Res. 26-8:1215-22 Anttila 2003. A new modified gamma-%CDT method improves the detection of problem drinking: studies in alcoholics with or without liver disease. Clin Chim Acta. 338-1-2:45-51 Anttila 2005. Biomarkers of alcohol consumption in patients classified according to the degree of liver disease severity. Scand J Clin Lab Invest. 65-2:141-51 Aradottir 2006. PHosphatidylethanol (PEth) concentrations in blood are correlated to reported alcohol intake in alcohol-dependent patients. Alcohol Alcohol. 41-4:431-7 Arumalla 2012. Sensitivity, specificity and diagnostic efficiency of serum sialic acid as a biochemical marker in alcohol abuse. British Journal of Medical Practitioners. 5-2: Bell 1993. Serum carbohydrate-deficient transferrin as a marker of alcohol consumption in patients with chronic liver diseases. Alcohol Clin Exp Res. 17-2:246-52 Bell 1994. Carbohydrate-deficient transferrin and other markers of high alcohol consumption: a study of 502 patients admitted consecutively to a medical department. Alcohol Clin Exp Res. 18-5:1103-8 Brathen 2001. Detection of alcohol abuse in neurological patients: variables of clinical relevance to the accuracy of the %CDT-TIA and CDTect methods. Alcohol Clin Exp Res. 25-1:46-53 Bush 1987. Screening for alcohol abuse using the CAGE questionnaire. Am J Med. 82-2:231-5 Chrostek 2006. The diagnostic accuracy of carbohydrate-deficient transferrin, sialic acid and commonly used markers of alcohol abuse during abstinence. Clin Chim Acta. 364-1-2:167-71 Conigrave 2002. CDT, GGT, and AST as markers of alcohol use: the WHO/ISBRA collaborative project. Alcohol Clin Exp Res. 26-3:332-9 Coulton 2006. Opportunistic screening for alcohol use disorders in primary care: comparative study. Bmj. 332-7540:511-7 Dixit 2015. Usefulness of gamma glutamyl transferase as reliable biological marker in objective corroboration of relapse in alcohol dependent patients. Journal of Clinical and Diagnostic Research. 9-12:VC01-VC04 Escobar 1995. Diagnostic tests for alcoholism in primary health care: compared efficacy of different instruments. 40-2:151-8 Fagerberg 1994. Is carbohydrate-deficient transferrin in serum useful for detecting excessive alcohol consumption in hypertensive patients?. Clin Chem. 40-11 Pt 1:2057-63 Girela 1994. Comparison of the CAGE questionnaire versus some biochemical markers in the diagnosis of alcoholism. Alcohol Alcohol. 29-3:337-43 Godart 2005. Carbohydrate-deficient transferrin and gamma-glutamyl transpeptidase in the evaluation of alcohol consumption. A five-year retrospective study of 633 outpatients in a single center. Gastroenterol Clin Biol. 29-2:113-6 Gomez 2001. Diagnostic usefulness of carbohydrate-deficient transferrin for detecting alcohol-related problems in hospitalized patients. Alcohol Alcohol. 36-3:266-70 Gough 2015. The Utility of Commonly Used Laboratory Tests to Screen for Excessive Alcohol Use in Clinical Practice. Alcoholism: Clinical and Experimental Research. 39-8:1493-1500 GÃ³mez 2006. The diagnostic usefulness of AUDIT and AUDIT-C for detecting hazardous drinkers in the elderly. Aging & Mental Health. 10-5:558-561 Halm 1999. Relative versus absolute carbohydrate-deficient transferrin as a marker of alcohol consumption in patients with acute alcoholic hepatitis. Alcohol Clin Exp Res. 23-10:1614-8 Hannuksela 1992. Evaluation of plasma cholesteryl ester transfer protein (CETP) activity as a marker of alcoholism. Alcohol Alcohol. 27-5:557-62 Hastedt 2013. Detecting alcohol abuse: traditional blood alcohol markers compared to ethyl glucuronide (EtG) and fatty acid ethyl esters (FAEEs) measurement in hair. Forensic Sci Med Pathol. 9-4:471-7 Hazelett 1998. Evaluation of acetaldehyde-modified hemoglobin and other markers of chronic heavy alcohol use: effects of gender and hemoglobin concentration. Alcohol Clin Exp Res. 22-8:1813-9 Huseby 1997. Evaluation of two biological markers combined as a parameter of alcohol dependency. Alcohol Alcohol. 32-6:731-7 Korzec 2005. The Bayesian Alcoholism Test had better diagnostic properties for confirming diagnosis of hazardous and harmful alcohol use. 58-10:1024-32 Korzec 2009. Validation of the Bayesian Alcoholism Test compared to single biomarkers in detecting harmful drinking. Alcohol and Alcoholism. 44-4:398-402 Kwoh-Gain 1990. Desialylated transferrin and mitochondrial aspartate aminotransferase compared as laboratory markers of excessive alcohol consumption. Clin Chem. 36-6:841-5 Lesch 1996. Carbohydrate-deficient transferrin as a screening marker for drinking in a general hospital population. Alcohol Alcohol. 31-3:249-56 Levola 2015. Screening for atâ€_risk drinking in a population reporting symptoms of depression: A validation of the AUDIT, AUDIT-C, and AUDIT-3. Alcoholism: Clinical and Experimental Research. 39-7:1186-1192 Liangpunsakul 2010. Relationship between alcohol drinking and aspartate aminotransferase:alanine aminotransferase (AST:ALT) ratio, mean corpuscular volume (MCV), gamma-glutamyl transpeptidase (GGT), and apolipoprotein A1 and B in the U.S. population. 71-2:249-52 Madhubala 2013. Serum carbohydrate deficient transferrin as a sensitive marker in diagnosing alcohol abuse: A case - Control study. Journal of Clinical and Diagnostic Research. 7-2:197-200 McDonald 2013. Comparative performance of biomarkers of alcohol consumption in a population sample of working-aged men in Russia: the Izhevsk Family Study. Addiction. 108-9:1579-89 Meerkerk 1999. Comparing the diagnostic accuracy of carbohydrate-deficient transferrin, Î³-Glutamyltransferase, and mean cell volume in a general practice population. Alcoholism: Clinical and Experimental Research. 23-6:1052-1059 Meregalli 1995. Carbohydrate-deficient transferrin in alcohol and nonalcohol abusers with liver disease. Alcohol Clin Exp Res. 19-6:1525-7 Mitchell 1997. Carbohydrate deficient transferrin in detecting relapse in alcohol dependence. 48-2:97-103 Morini 2011. Chronic excessive alcohol consumption diagnosis: comparison between traditional biomarkers and ethyl glucuronide in hair, a study on a real population. 33-5:654-7 Mundle 1999. Biological markers as indicators for relapse in alcohol-dependent patients. Addiction Biology. 4-2:209-214 Mundle 2000. Sex differences of carbohydrate-deficient transferrin, gamma-glutamyltransferase, and mean corpuscular volume in alcohol-dependent patients. Alcohol Clin Exp Res. 24-9:1400-5 Naik 1995. How can we detect sick elderly excessive drinkers?. International Journal of Geriatric Psychiatry. 10-12:1063-1066 Nalpas 1989. Evaluation of mAST/tAST ratio as a marker of alcohol misuse in a non-selected population. Alcohol Alcohol. 24-5:415-9 Neumann 2008. Value of ethyl glucuronide in plasma as a biomarker for recent alcohol consumption in the emergency room. Alcohol Alcohol. 43-4:431-5 Neumann 2009. Screening trauma patients with the alcohol use disorders identification test and biomarkers of alcohol use. Alcohol Clin Exp Res. 33-6:970-6 Niemela 1995. Carbohydrate-deficient transferrin as a marker of alcohol abuse: relationship to alcohol consumption, severity of liver disease, and fibrogenesis. Alcohol Clin Exp Res. 19-5:1203-8 Nilssen 1992. New alcohol markersâ€”how useful are they in population studies: The Svalbard study 1988â€“89. Alcoholism: Clinical and Experimental Research. 16-1:82-86 Nystrom 1992. Carbohydrate-deficient transferrin (CDT) in serum as a possible indicator of heavy drinking in young university students. Alcohol Clin Exp Res. 16-1:93-7 Piano 2014. Assessment of alcohol consumption in liver transplant candidates and recipients: the best combination of the tools available. Liver Transpl. 20-7:815-22 Pirro 2011. Chemometric evaluation of nine alcohol biomarkers in a large population of clinically-classified subjects: pre-eminence of ethyl glucuronide concentration in hair for confirmatory classification. Anal Bioanal Chem. 401-7:2153-64 Radosavljevic 1995. Elevated levels of serum carbohydrate deficient transferrin are not specific for alcohol abuse in patients with liver disease. J Hepatol. 23-6:706-11 Reynaud 2000. Objective diagnosis of alcohol abuse: compared values of carbohydrate-deficient transferrin (CDT), gamma-glutamyl transferase (GGT), and mean corpuscular volume (MCV). Alcohol Clin Exp Res. 24-9:1414-9 Rinck 2007. Combinations of carbohydrate-deficient transferrin, mean corpuscular erythrocyte volume, gamma-glutamyltransferase, homocysteine and folate increase the significance of biological markers in alcohol dependent patients. 89-1:60-5 Rublo 1997. Carbohydrate-deficient transferrin as a marker of alcohol consumption in male patients with liver disease. Alcohol Clin Exp Res. 21-5:923-7 Ryb 1999. Use of blood alcohol concentration and laboratory tests to detect current alcohol dependence in trauma center patients. J Trauma. 47-5:874-9; discussion 879-80 Savola 2004. Blood alcohol is the best indicator of hazardous alcohol drinking in young adults and working-age patients with trauma. Alcohol Alcohol. 39-4:340-5 Schellenberg 1989. Evaluation of carbohydrate-deficient transferrin compared with Tf index and other markers of alcohol abuse. Alcohol Clin Exp Res. 13-5:605-10 Schmidt 1997. Superiority of carbohydrate-deficient transferrin to gamma-glutamyltransferase in detecting relapse in alcoholism. Am J Psychiatry. 154-1:75-80 Schmitt 1998. Carbohydrate-deficient transferrin is not a useful marker for the detection of chronic alcohol abuse. Eur J Clin Invest. 28-8:615-21 Schwan 2004. Multicenter validation study of the %CDT TIA kit in alcohol abuse and alcohol dependence. Alcohol Clin Exp Res. 28-9:1331-7 Sillanaukee 1998. Carbohydrate-deficient transferrin and conventional alcohol markers as indicators for brief intervention among heavy drinkers in primary health care. Alcohol Clin Exp Res. 22-4:892-6 Sillanaukee 1999. Sialic acid: new potential marker of alcohol abuse. Alcohol Clin Exp Res. 23-6:1039-43 Sillanaukee 2000. Enhanced clinical utility of gamma-CDT in a general population. Alcohol Clin Exp Res. 24-8:1202-6 Skinner 1984. Identification of alcohol abuse using laboratory tests and a history of trauma. Ann Intern Med. 101-6:847-51 Spies 1995. Relevance of carbohydrate-deficient transferrin as a predictor of alcoholism in intensive care patients following trauma. J Trauma. 39-4:742-8 Staufer 2011. Urinary ethyl glucuronide as a novel screening tool in patients pre- and post-liver transplantation improves detection of alcohol consumption. Hepatology. 54-5:1640-9 Stowell 1997. Comparison of two commercial test kits for quantification of serum carbohydrate-deficient transferrin. Alcohol Alcohol. 32-4:507-16 Taracha 2002. The activity of beta-hexosaminidase (uHex) and gamma-glutamyltransferase (uGGT) in urine as non-invasive markers of chronic alcohol abuse: II. Opiate-dependent subjects receiving methadone substitution. World J Biol Psychiatry. 3-1:44-9 Thiesen 2010. Biological markers of problem drinking in homeless patients. 35-3:260-2 Werle 1997. High-performance liquid chromatography improves diagnostic efficiency of carbohydrate-deficient transferrin. Alcohol Alcohol. 32-1:71-7 Wetterling 1998. Comparison of cage and mast with the alcohol markers CDT, gamma-GT, ALAT, ASAT and MCV. Alcohol Alcohol. 33-4:424-30 Whitfield 2008. Measuring carbohydrate-deficient transferrin by direct immunoassay: factors affecting diagnostic sensitivity for excessive alcohol intake. Clin Chem. 54-7:1158-65 Yeastedt 1998. Female alcoholic outpatients and female college students: a correlational study of self-reported alcohol consumption and carbohydrate-deficient transferrin levels. J Stud Alcohol. 59-5:555-9 Yersin 1995. Screening for excessive alcohol drinking. Comparative value of carbohydrate-deficient transferrin, gamma-glutamyltransferase, and mean corpuscular volume. Arch Intern Med. 155-17:1907-11 Zierau 2005. Validation of a self-administered modified CAGE test (CAGE-C) in a somatic hospital ward: comparison with biochemical markers. Scand J Clin Lab Invest. 65-7:615-22 |
| 892  GGT-MCV | Aradottir 2006. PHosphatidylethanol (PEth) concentrations in blood are correlated to reported alcohol intake in alcohol-dependent patients. Alcohol Alcohol. 41-4:431-7 Gul 2005. [The diagnostic validity of screening tests and laboratory markers in alcohol use disorders]. Turk Psikiyatri Derg. 16-1:3-12 Liangpunsakul 2010. Relationship between alcohol drinking and aspartate aminotransferase:alanine aminotransferase (AST:ALT) ratio, mean corpuscular volume (MCV), gamma-glutamyl transpeptidase (GGT), and apolipoprotein A1 and B in the U.S. population. 71-2:249-52 Mundle 1999. Biological markers as indicators for relapse in alcohol-dependent patients. Addiction Biology. 4-2:209-214 Mundle 2000. Sex differences of carbohydrate-deficient transferrin, gamma-glutamyltransferase, and mean corpuscular volume in alcohol-dependent patients. Alcohol Clin Exp Res. 24-9:1400-5 Radosavljevic 1995. Elevated levels of serum carbohydrate deficient transferrin are not specific for alcohol abuse in patients with liver disease. J Hepatol. 23-6:706-11 Reynaud 2000. Objective diagnosis of alcohol abuse: compared values of carbohydrate-deficient transferrin (CDT), gamma-glutamyl transferase (GGT), and mean corpuscular volume (MCV). Alcohol Clin Exp Res. 24-9:1414-9 Sillanaukee 1998. Carbohydrate-deficient transferrin and conventional alcohol markers as indicators for brief intervention among heavy drinkers in primary health care. Alcohol Clin Exp Res. 22-4:892-6 |
| 956  EtG | Hastedt 2013. Detecting alcohol abuse: traditional blood alcohol markers compared to ethyl glucuronide (EtG) and fatty acid ethyl esters (FAEEs) measurement in hair. Forensic Sci Med Pathol. 9-4:471-7 Lees 2012. Comparison of ethyl glucuronide in hair with self-reported alcohol consumption. Alcohol Alcohol. 47-3:267-72 Morini 2009. Ethyl glucuronide in hair. A sensitive and specific marker of chronic heavy drinking. Addiction. 104-6:915-20 Morini 2011. Chronic excessive alcohol consumption diagnosis: comparison between traditional biomarkers and ethyl glucuronide in hair, a study on a real population. 33-5:654-7 Wurst 2004. On sensitivity, specificity, and the influence of various parameters on ethyl glucuronide levels in urine--results from the WHO/ISBRA study. Alcohol Clin Exp Res. 28-8:1220-8 |
| 1016  MAST | Clements 1998. A critical evaluation of several alcohol screening instruments using the CIDI-SAM as a criterion measure. Alcohol Clin Exp Res. 22-5:985-93 Conley 2001. Construct validity of the MAST and AUDIT with multiple offender drunk drivers. Journal of Substance Abuse Treatment. 20-4:287-295 Conley 2006. Court Ordered Multiple Offender Drunk Drivers: Validity and Reliability of Rapid Assessment. Journal of Social Work Practice in the Addictions. 6-3:37-51 Drake 1990. Diagnosis of Alcohol Use Disorders in Schizophrenia. Schizophrenia Bulletin. 16-1:57-67 Garzotto 1988. Validation of a screening questionnaire for alcoholism (MAST) in an Italian sample. Comprehensive Psychiatry. 29-3:323-329 Hirata 2001. Validity of the Michigan Alcoholism Screening Test (MAST) for the detection of alcohol-related problems among male geriatric outpatients. Am J Geriatr Psychiatry. 9-1:30-4 Mischke 1987. Reliability and validity of the MAST, Mortimer-Filkins Questionnaire and CAGE in DWI assessment. J Stud Alcohol. 48-5:492-501 Murray 1994. The relative performance of diverse measures of alcohol abuse and dependence in a community sample. J Stud Alcohol. 55-1:72-80 Myerholtz 1997. Screening DUI offenders for alcohol problems: Psychometric assessment of the substance abuse subtle screening inventory. Psychology of Addictive Behaviors. 11-3:155-165 Rosenberg 1998. Dartmouth Assessment of Lifestyle Instrument (DALI): a substance use disorder screen for people with severe mental illness. Am J Psychiatry. 155-2:232-8 Rumpf 1998. Screening questionnaires in the detection of hazardous alcohol consumption in the general hospital: direct or disguised assessment?. J Stud Alcohol. 59-6:698-703 Russell 1994. Screening for pregnancy risk-drinking. Alcohol Clin Exp Res. 18-5:1156-61 Russell 1996. Detecting risk drinking during pregnancy: a comparison of four screening questionnaires. Am J Public Health. 86-10:1435-9 Sheridan 1995. Substance abuse in freshmen medical students over time. Substance Abuse. 16-2:93-98 Sokol 1989. The T-ACE questions: practical prenatal detection of risk-drinking. 160-4:863-8; discussion 868-70 Svanum 1995. Prospective screening of substance dependence: the advantages of directness. 20-2:205-13 Teitelbaum 2000. Temporal stability of alcohol screening measures in a psychiatric setting. Psychol Addict Behav. 14-4:401-4 Wetterling 1998. Comparison of cage and mast with the alcohol markers CDT, gamma-GT, ALAT, ASAT and MCV. Alcohol Alcohol. 33-4:424-30 Yersin 1989. Accuracy of the Michigan Alcoholism Screening Test for screening of alcoholism in patients of a medical department. Arch Intern Med. 149-9:2071-4 |
| 1020  MCV | Aithal 1998. Measurement of carbohydrate-deficient transferrin (CDT) in a general medical clinic: is this test useful in assessing alcohol consumption. Alcohol Alcohol. 33-3:304-9 Anttila 2003. A new modified gamma-%CDT method improves the detection of problem drinking: studies in alcoholics with or without liver disease. Clin Chim Acta. 338-1-2:45-51 Anttila 2005. Biomarkers of alcohol consumption in patients classified according to the degree of liver disease severity. Scand J Clin Lab Invest. 65-2:141-51 Aradottir 2006. PHosphatidylethanol (PEth) concentrations in blood are correlated to reported alcohol intake in alcohol-dependent patients. Alcohol Alcohol. 41-4:431-7 Bell 1994. Carbohydrate-deficient transferrin and other markers of high alcohol consumption: a study of 502 patients admitted consecutively to a medical department. Alcohol Clin Exp Res. 18-5:1103-8 Bush 1987. Screening for alcohol abuse using the CAGE questionnaire. Am J Med. 82-2:231-5 Chrostek 2006. The diagnostic accuracy of carbohydrate-deficient transferrin, sialic acid and commonly used markers of alcohol abuse during abstinence. Clin Chim Acta. 364-1-2:167-71 Coulton 2006. Opportunistic screening for alcohol use disorders in primary care: comparative study. BMJ. 332-7540:511-7 Escobar 1995. Diagnostic tests for alcoholism in primary health care: compared efficacy of different instruments. 40-2:151-8 Girela 1994. Comparison of the CAGE questionnaire versus some biochemical markers in the diagnosis of alcoholism. Alcohol. 29-3:337-43 Gomez 2001. Diagnostic usefulness of carbohydrate-deficient transferrin for detecting alcohol-related problems in hospitalized patients. Alcohol. 36-3:266-70 Gough 2015. The Utility of Commonly Used Laboratory Tests to Screen for Excessive Alcohol Use in Clinical Practice. Alcoholism: Clinical and Experimental Research. 39-8:1493-1500 GÃ³mez 2006. The diagnostic usefulness of AUDIT and AUDIT-C for detecting hazardous drinkers in the elderly. Aging & Mental Health. 10-5:558-561 Halm 1999. Relative versus absolute carbohydrate-deficient transferrin as a marker of alcohol consumption in patients with acute alcoholic hepatitis. Alcohol Clin Exp Res. 23-10:1614-8 Hannuksela 1992. Evaluation of plasma cholesteryl ester transfer protein (CETP) activity as a marker of alcoholism. Alcohol. 27-5:557-62 Hastedt 2013. Detecting alcohol abuse: traditional blood alcohol markers compared to ethyl glucuronide (EtG) and fatty acid ethyl esters (FAEEs) measurement in hair. Forensic Sci Med Pathol. 9-4:471-7 Hock 2005. Validity of carbohydrate-deficient transferrin (%CDT),Î³-glutamyltransferase (Î³-GT) and mean corpuscular erythrocyte volume (MCV) as biomarkers for chronic alcohol abuse: A study in patients with alcohol dependence and liver disorders of non-alcoholic and alcoholic origin. Addiction. 100-10:1477-1486 Kwoh-Gain 1990. Desialylated transferrin and mitochondrial aspartate aminotransferase compared as laboratory markers of excessive alcohol consumption. Clin Chem. 36-6:841-5 Liangpunsakul 2010. Relationship between alcohol drinking and aspartate aminotransferase:alanine aminotransferase (AST:ALT) ratio, mean corpuscular volume (MCV), gamma-glutamyl transpeptidase (GGT), and apolipoprotein A1 and B in the U.S. population. 71-2:249-52 Luttrell 1997. Screening for alcohol misuse in older people. 12-12:1151-4 Madhubala 2013. Serum carbohydrate deficient transferrin as a sensitive marker in diagnosing alcohol abuse: A case - Control study. Journal of Clinical and Diagnostic Research. 7-2:197-200 McDonald 2013. Comparative performance of biomarkers of alcohol consumption in a population sample of working-aged men in Russia: the Izhevsk Family Study. Addiction. 108-9:1579-89 Meerkerk 1999. Comparing the diagnostic accuracy of carbohydrate-deficient transferrin, Î³-Glutamyltransferase, and mean cell volume in a general practice population. Alcoholism: Clinical and Experimental Research. 23-6:1052-1059 Meregalli 1995. Carbohydrate-deficient transferrin in alcohol and nonalcohol abusers with liver disease. Alcohol Clin Exp Res. 19-6:1525-7 Morini 2011. Chronic excessive alcohol consumption diagnosis: comparison between traditional biomarkers and ethyl glucuronide in hair, a study on a real population. 33-5:654-7 Mundle 1999. Biological markers as indicators for relapse in alcohol-dependent patients. Addiction Biology. 4-2:209-214 Mundle 2000. Sex differences of carbohydrate-deficient transferrin, gamma-glutamyltransferase, and mean corpuscular volume in alcohol-dependent patients. Alcohol Clin Exp Res. 24-9:1400-5 Naik 1995. How can we detect sick elderly excessive drinkers?. International Journal of Geriatric Psychiatry. 10-12:1063-1066 Nalpas 1989. Evaluation of mAST/tAST ratio as a marker of alcohol misuse in a non-selected population. Alcohol Alcohol. 24-5:415-9 Neumann 2009. Screening trauma patients with the alcohol use disorders identification test and biomarkers of alcohol use. Alcohol Clin Exp Res. 33-6:970-6 Niemela 1995. Carbohydrate-deficient transferrin as a marker of alcohol abuse: relationship to alcohol consumption, severity of liver disease, and fibrogenesis. Alcohol Clin Exp Res. 19-5:1203-8 Piano 2014. Assessment of alcohol consumption in liver transplant candidates and recipients: the best combination of the tools available. Liver Transpl. 20-7:815-22 Pirro 2011. Chemometric evaluation of nine alcohol biomarkers in a large population of clinically-classified subjects: pre-eminence of ethyl glucuronide concentration in hair for confirmatory classification. Anal Bioanal Chem. 401-7:2153-64 Radosavljevic 1995. Elevated levels of serum carbohydrate deficient transferrin are not specific for alcohol abuse in patients with liver disease. J Hepatol. 23-6:706-11 Reid 2005. Are Commonly Ordered Lab Tests Useful Screens for Alcohol Disorders in Older Male Veterans Receiving Primary Care?. Substance Abuse. 26-2:25-32 Reynaud 1998. Usefulness of carbohydrate-deficient transferrin in alcoholic patients with normal gamma-glutamyltranspeptidase. Alcohol Clin Exp Res. 22-3:615-8 Reynaud 2000. Objective diagnosis of alcohol abuse: compared values of carbohydrate-deficient transferrin (CDT), gamma-glutamyl transferase (GGT), and mean corpuscular volume (MCV). Alcohol Clin Exp Res. 24-9:1414-9 Rinck 2007. Combinations of carbohydrate-deficient transferrin, mean corpuscular erythrocyte volume, gamma-glutamyltransferase, homocysteine and folate increase the significance of biological markers in alcohol dependent patients. 89-1:60-5 Rublo 1997. Carbohydrate-deficient transferrin as a marker of alcohol consumption in male patients with liver disease. Alcohol Clin Exp Res. 21-5:923-7 Ryb 1999. Use of blood alcohol concentration and laboratory tests to detect current alcohol dependence in trauma center patients. J Trauma. 47-5:874-9; discussion 879-80 Savola 2004. Blood alcohol is the best indicator of hazardous alcohol drinking in young adults and working-age patients with trauma. Alcohol Alcohol. 39-4:340-5 Schmitt 1998. Carbohydrate-deficient transferrin is not a useful marker for the detection of chronic alcohol abuse. Eur J Clin Invest. 28-8:615-21 Sillanaukee 1998. Carbohydrate-deficient transferrin and conventional alcohol markers as indicators for brief intervention among heavy drinkers in primary health care. Alcohol Clin Exp Res. 22-4:892-6 Sillanaukee 1999. Sialic acid: new potential marker of alcohol abuse. Alcohol Clin Exp Res. 23-6:1039-43 Skinner 1984. Identification of alcohol abuse using laboratory tests and a history of trauma. Ann Intern Med. 101-6:847-51 Spies 1995. Relevance of carbohydrate-deficient transferrin as a predictor of alcoholism in intensive care patients following trauma. J Trauma. 39-4:742-8 Staufer 2011. Urinary ethyl glucuronide as a novel screening tool in patients pre- and post-liver transplantation improves detection of alcohol consumption. Hepatology. 54-5:1640-9 Stowell 1997. Comparison of two commercial test kits for quantification of serum carbohydrate-deficient transferrin. Alcohol Alcohol. 32-4:507-16 Thiesen 2010. Biological markers of problem drinking in homeless patients. 35-3:260-2 Wetterling 1998. Comparison of cage and mast with the alcohol markers CDT, gamma-GT, ALAT, ASAT and MCV. Alcohol Alcohol. 33-4:424-30 Yersin 1995. Screening for excessive alcohol drinking. Comparative value of carbohydrate-deficient transferrin, gamma-glutamyltransferase, and mean corpuscular volume. Arch Intern Med. 155-17:1907-11 |
| 1130  PEth | Aradottir 2006. PHosphatidylethanol (PEth) concentrations in blood are correlated to reported alcohol intake in alcohol-dependent patients. Alcohol Alcohol. 41-4:431-7 Hahn 2012. Phosphatidylethanol (PEth) as a biomarker of alcohol consumption in HIV-positive patients in sub-Saharan Africa. Alcohol Clin Exp Res. 36-5:854-62 Jain 2014. Comparison of phosphatidylethanol results to self-reported alcohol consumption among young injection drug users. Alcohol Alcohol. 49-5:520-4 Kechagias 2015. Phosphatidylethanol Compared with Other Blood Tests as a Biomarker of Moderate Alcohol Consumption in Healthy Volunteers: A Prospective Randomized Study. Alcohol Alcohol. 50-4:399-406 Kwak 2014. Characterization of phosphatidylethanol blood concentrations for screening alcohol consumption in early pregnancy. Clin Toxicol (Phila). 52-1:25-31 Stewart 2014. Validation of blood phosphatidylethanol as an alcohol consumption biomarker in patients with chronic liver disease. Alcohol Clin Exp Res. 38-6:1706-11 Walther 2015. Phosphatidylethanol is Superior to Carbohydrate-Deficient Transferrin and Î³-Glutamyltransferase as an Alcohol Marker and is a Reliable Estimate of Alcohol Consumption Level. Alcoholism: Clinical and Experimental Research. 39-11:2200-2208 Wurst 2010. Phosphatidylethanol: normalization during detoxification, gender aspects and correlation with other biomarkers and self-reports. 15-1:88-95 |
| 1131  POSIT | Knight 2003. Validity of brief alcohol screening tests among adolescents: A comparison of the AUDIT, POSIT, CAGE, and CRAFFT. Alcoholism: Clinical and Experimental Research. 27-1:67-73 Rumpf 2013. Screening questionnaires for problem drinking in adolescents: performance of AUDIT, AUDIT-C, CRAFFT and POSIT. 19-3:121-7 |
| 1230  SAAST | Cornel 1994. Problem drinking in a general practice population: the construction of an interval scale for severity of problem drinking. J Stud Alcohol. 55-4:466-70 Gates 2007. Alcohol screening instruments and psychiatric evaluation outcomes in military aviation personnel. Aviat Space Environ Med. 78-1:48-51 Steinbauer 1998. Ethnic and sex bias in primary care screening tests for alcohol use disorders. Ann Intern Med. 129-5:353-62 Vickers-Douglas 2005. Revision of the Self-Administered Alcoholism Screening Test (SAAST-R): a pilot study. Subst Use Misuse. 40-6:789-812 |
| 1267  SSADDA | Pierucci L 2005. Diagnostic reliability of the Semi-structured Assessment for Drug Dependence and Alcoholism (SSADDA). Drug Alcohol Depend. 80-3:303-12 |
| 1319  SDS | Chen 2008. Severity of heroin dependence in Taiwan: reliability and validity of the Chinese version of the Severity of Dependence Scale (SDS[Ch]). 33-12:1590-3 Gossop 2002. Dual dependence: assessment of dependence upon alcohol and illicit drugs, and the relationship of alcohol dependence among drug misusers to patterns of drinking, illicit drug use and health problems. Addiction. 97-2:169-78 Kavanagh 2011. The DrugCheck Problem List: a new screen for substance use disorders in people with psychosis. 36-9:927-32 Lawrinson 2007. Determining a cut-off on the Severity of Dependence Scale (SDS) for alcohol dependence. 32-7:1474-9 |
| 1402  TACE | Chang 2006. Identifying Risk Drinking in Expectant Fathers. Birth: Issues in Perinatal Care. 33-2:110-116 Chang 2010. Identification of risk drinking women: T-ACE screening tool or the medical record. J Womens Health (Larchmt). 19-10:1933-9 McQuade 2000. Detecting symptoms of alcohol abuse in primary care settings. Arch Fam Med. 9-9:814-21 Moraes 2005. Assessing alcohol misuse during pregnancy: evaluating psychometric properties of the CAGE, T-ACE and TWEAK in a Brazilian setting. J Stud Alcohol. 66-2:165-73 Oliveira 2011. Alcohol screening instruments in elderly male: a population-based survey in metropolitan Sao Paulo, Brazil. Rev Bras Psiquiatr. 33-4:347-52 Rosenberg 1998. Dartmouth Assessment of Lifestyle Instrument (DALI): a substance use disorder screen for people with severe mental illness. Am J Psychiatry. 155-2:232-8 Russell 1994. Screening for pregnancy risk-drinking. Alcohol Clin Exp Res. 18-5:1156-61 Russell 1996. Detecting risk drinking during pregnancy: a comparison of four screening questionnaires. Am J Public Health. 86-10:1435-9 Sarkar 2010. Comparing the effectiveness of TWEAK and T-ACE in determining problem drinkers in pregnancy. Alcohol and Alcoholism. 45-4:356-360 Sokol 1989. The T-ACE questions: practical prenatal detection of risk-drinking. 160-4:863-8; discussion 868-70 |
| 1405  TWEAK | Fals-Stewart 2000. The timeline followback reports of psychoactive substance use by drug-abusing patients: psychometric properties. J Consult Clin Psychol. 68-1:134-44 Fiellin 2013. Measuring alcohol consumption using Timeline Followback in non-treatment-seeking medical clinic patients with and without HIV infection: 7-, 14-, or 30-day recall. 74-3:500-4 Sacks 2003. Utility of the Time-Line Followback to assess substance use among homeless adults. J Nerv Ment Dis. 191-3:145-53 Staines 2001. Polysubstance use among alcoholics. J Addict Dis. 20-4:53-69 |
| 1407 | Cherpitel 1995. Ethnic differences in performance of screening instruments for identifying harmful drinking and alcohol dependence in the emergency room. Alcohol Clin Exp Res. 19-3:628-34 Cherpitel 1997. Comparison of screening instruments for alcohol problems between black and white emergency room patients from two regions of the country. Alcohol Clin Exp Res. 21-8:1391-7 Cherpitel 1998. Differences in performance of screening instruments for problem drinking among blacks, whites and Hispanics in an emergency room population. J Stud Alcohol. 59-4:420-6 Cherpitel 2000. Screening instruments for alcohol problems: a comparison of cut points between Mexican American and Mexican patients in the emergency room. Subst Use Misuse. 35-10:1419-30 Cherpitel 2001. Screening for alcohol problems: A comparison of instrument performance among black emergency department and primary care patients. Journal of Substance Use. 5-4:290-297 Cremonte 2008. Performance of screening instruments for alcohol use disorders in emergency departments patients in Argentina. Substance Use & Misuse. 43-1:125-138 Cremonte 2010. Psychometric properties of alcohol screening tests in the emergency department in Argentina, Mexico and the United States. 35-9:818-25 Kelly 2002. A comparison of alcohol screening instruments among under-aged drinkers treated in emergency departments. Alcohol Alcohol. 37-5:444-50 Moraes 2005. Assessing alcohol misuse during pregnancy: evaluating psychometric properties of the CAGE, T-ACE and TWEAK in a Brazilian setting. J Stud Alcohol. 66-2:165-73 Oliveira 2011. Alcohol screening instruments in elderly male: a population-based survey in metropolitan Sao Paulo, Brazil. Rev Bras Psiquiatr. 33-4:347-52 Rosenberg 1998. Dartmouth Assessment of Lifestyle Instrument (DALI): a substance use disorder screen for people with severe mental illness. Am J Psychiatry. 155-2:232-8 Russell 1994. Screening for pregnancy risk-drinking. Alcohol Clin Exp Res. 18-5:1156-61 Russell 1996. Detecting risk drinking during pregnancy: a comparison of four screening questionnaires. Am J Public Health. 86-10:1435-9 Sarkar 2010. Comparing the effectiveness of TWEAK and T-ACE in determining problem drinkers in pregnancy. Alcohol and Alcoholism. 45-4:356-360 Wu 2008. Validation and comparison of alcohol-screening instruments for identifying hazardous drinking in hospitalized patients in Taiwan. Alcohol Alcohol. 43-5:577-82 |
| 1420  CUAD | Appleby 1996. Utility of the chemical use, abuse, and dependence scale in screening patients with severe mental illness. Psychiatr Serv. 47-6:647-9 |
